# Supplementary material for: High-resolution mapping of heteroduplex DNA formed during UV-induced and spontaneous mitotic recombination events in yeast
Source: eLife. 2017 Jul 17;6:e28069. doi: 10.7554/eLife.28069 (PMC5531827; doi:10.7554/eLife.28069)
Supplement: Supplementary file 2. — For all events, we show the initiating DSB on the blue chromatid. Dotted lines show sequences generated by replication or during mismatch repair. Black arrows indicate the position of the initiating DSB. The mechanisms are discussed in detail in the main text. Red and blue lines represent DNA strands of the W303-1A-derived chromatid and YJM789-derived chromatid, respectively. Arrows on these strands indicate the 3’ ends. A. Class 1. Class one events are NCOs formed by one-ended SDSA. B. Class 2. These NCO events could be generated by one-ended SDSA, followed by Mlh1p-independent MMR. C. Class 2. An alterative possibility is that Class two events reflect repair of a double-stranded DNA gap. D. Class 3. In this NCO class, the heteroduplex region is adjacent to a conversion tract. Such events could reflect a heteroduplex tract in which mismatches are repaired in one part of the tract and left unrepaired in the other. E. Class 3. An alternative model for this class is that the conversion tract is the result of repair of a double-stranded DNA gap with a heteroduplex region at one end. F. Class 4. For this CO class, a heteroduplex is observed on one chromatid but not the other. This class could be explained by the DSBR pathway in which heteroduplex region is short relative to the other; if the short heteroduplex does not contain a mismatch, it would be undetectable. Supplementary file 2 - S2. Mechanisms that generate Classes 6–8 recombination events. Events are depicted as in Supplementary file 2 - S1. A. Class 5. In this NCO class, the heteroduplex region is located on the opposite of the DSB site from the conversion region. This pattern is consistent with the repair of a double-stranded DNA gap that was restricted to one of the broken ends. B. Class 6. This CO class is identical to the pattern expected for the DSBR model. C. Class 7. In this CO class, no heteroduplexes or conversion tracts are observed adjacent to the crossover, consistent with the formation of a dHJ wi [file elife-28069-supp2.pdf]

S1

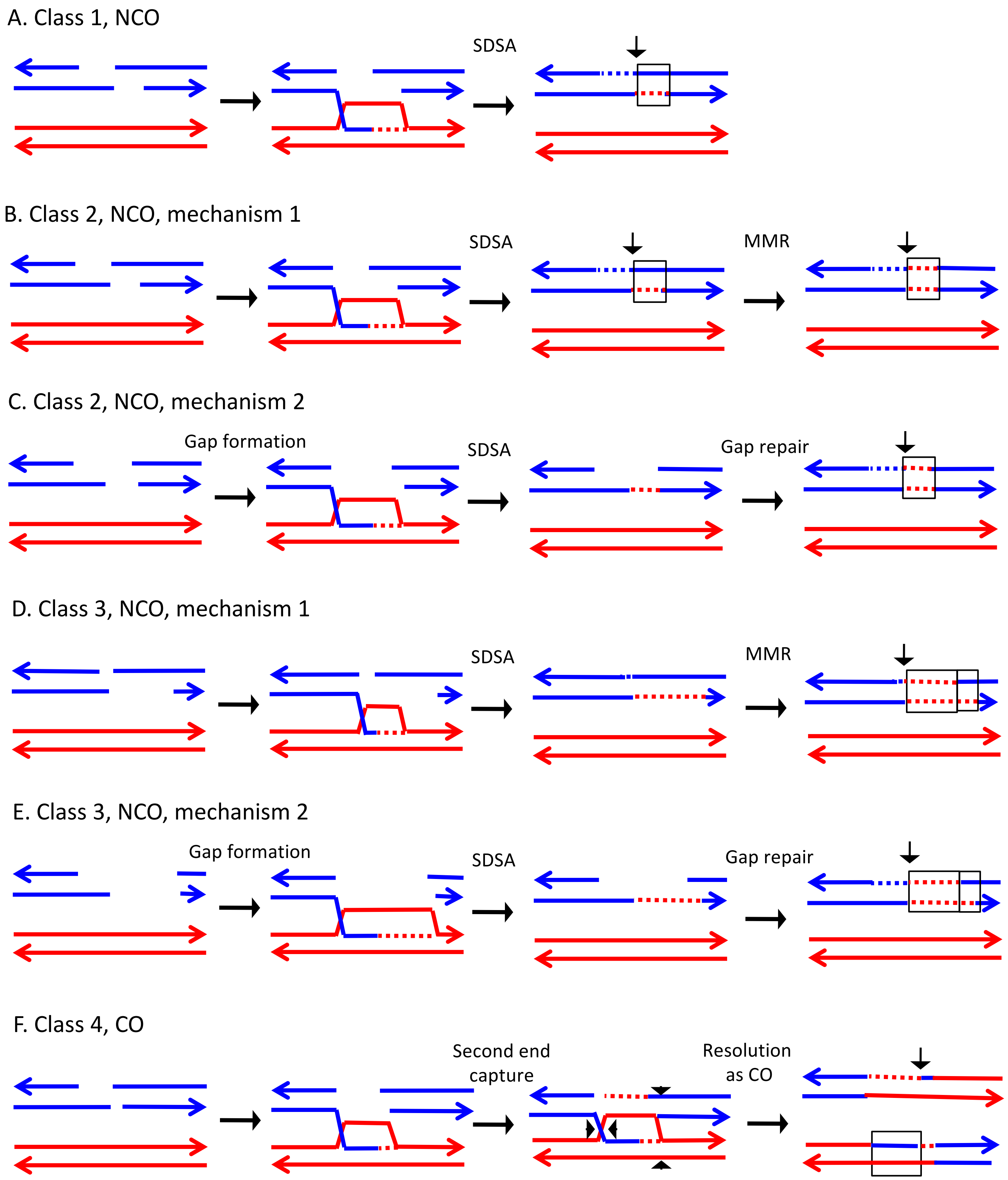
A. Class 1, NCO
SDSA
B. Class 2, NCO, mechanism 1
SDSA
MMR
C. Class 2, NCO, mechanism 2
Gap formation
SDSA
Gap repair
D. Class 3, NCO, mechanism 1
SDSA
MMR
E. Class 3, NCO, mechanism 2
Gap formation
SDSA
Gap repair
F. Class 4, CO
Second end
capture
Resolution
as CO

S2

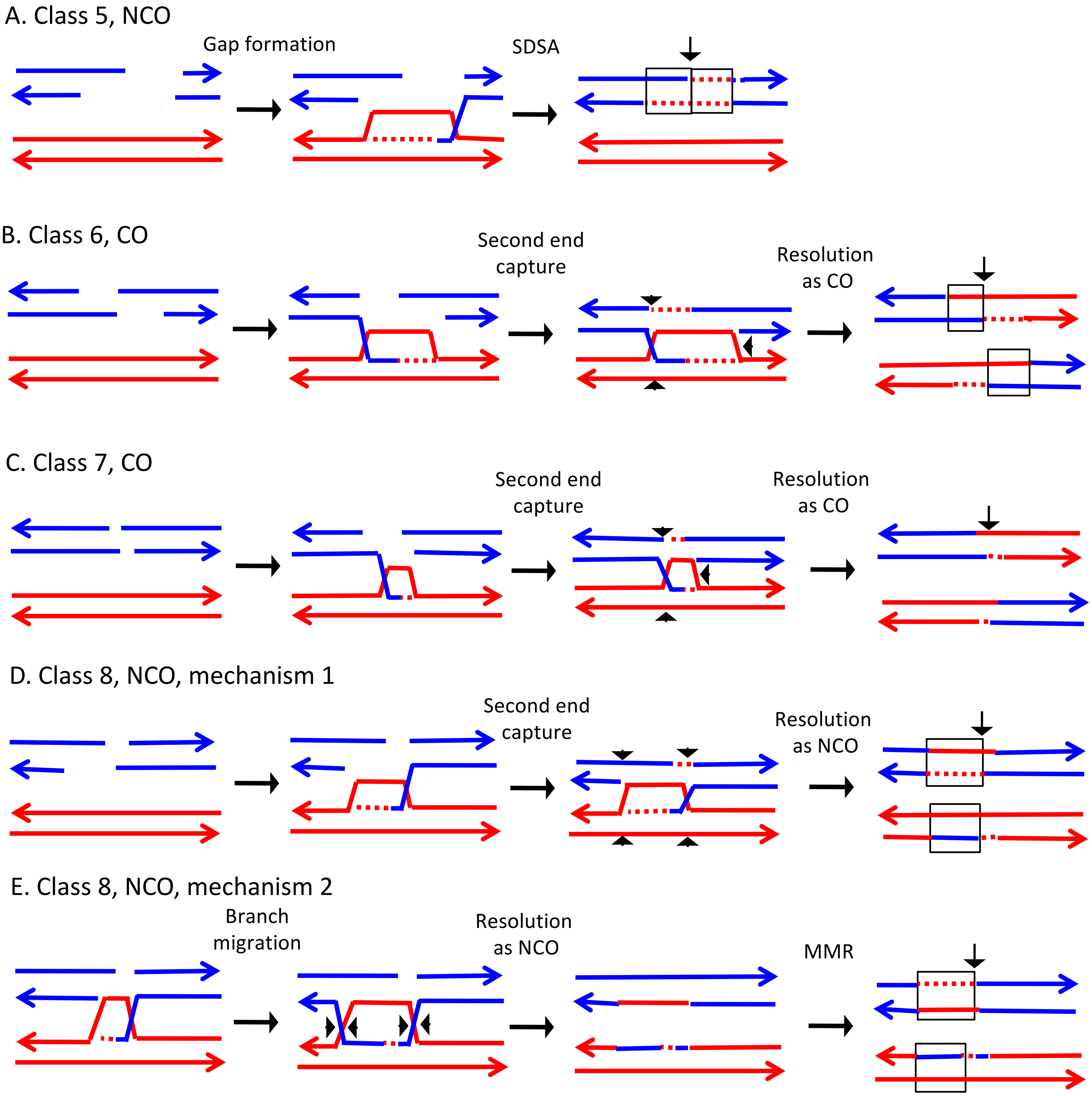
A. Class 5, NCO
Gap formation
SDSA
B. Class 6, CO
Second end capture
Resolution as CO
C. Class 7, CO
Second end capture
Resolution as CO
D. Class 8, NCO, mechanism 1
Second end capture
Resolution as NCO
E. Class 8, NCO, mechanism 2
Branch migration
Resolution as NCO
MMR

S3

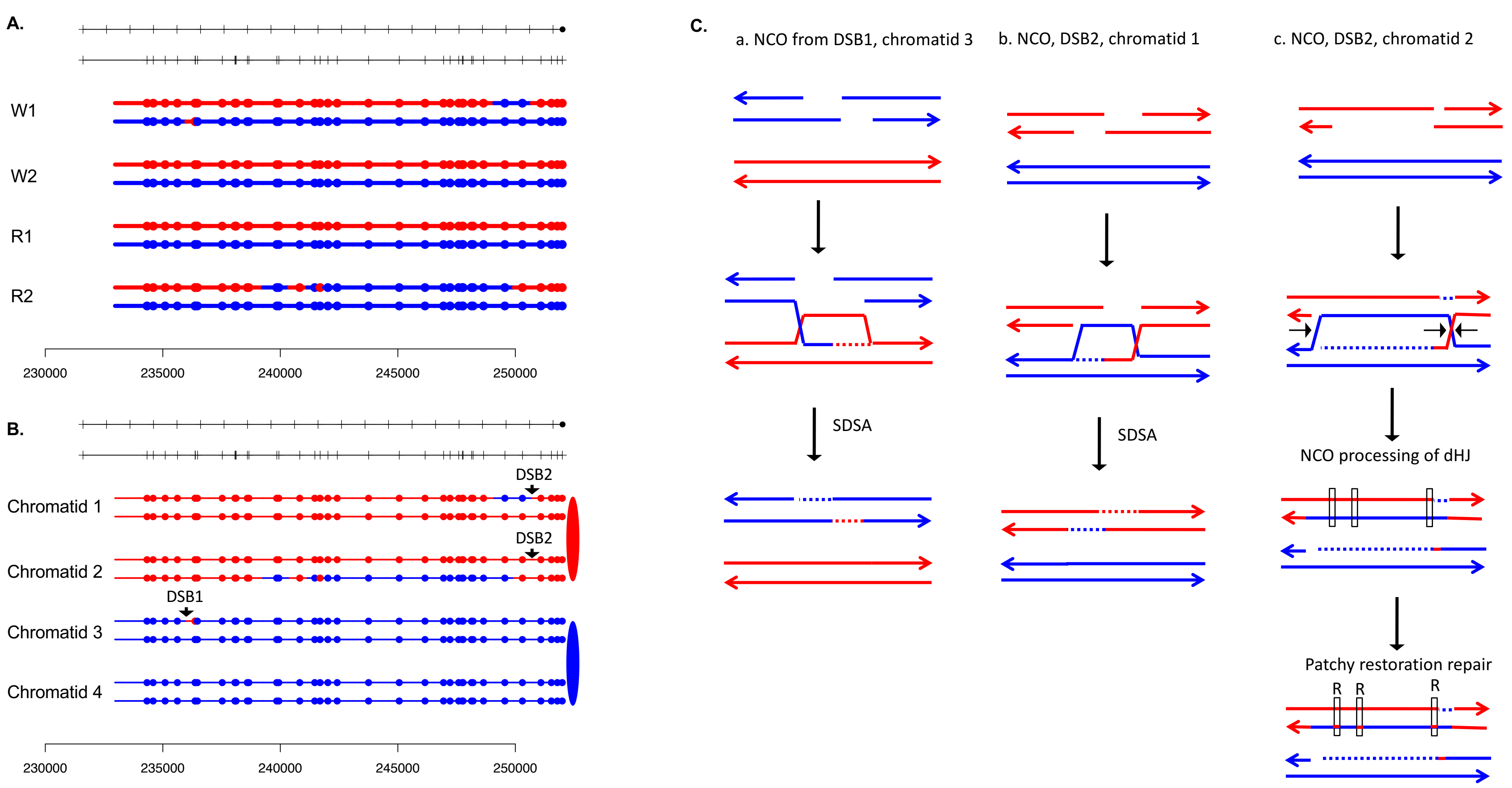

S3	NCO1	Simple heteroduplex. DSB on blue chromatid.	SDSA
	NCO2	Simple heteroduplex. DSB on red chromatid.	SDSA
	NCO3	Long heteroduplex with regions of homoduplex. DSB on red chromatid.	Standard DSBR event except Mlh1-independent MMR. Two independent DSBs.

S4

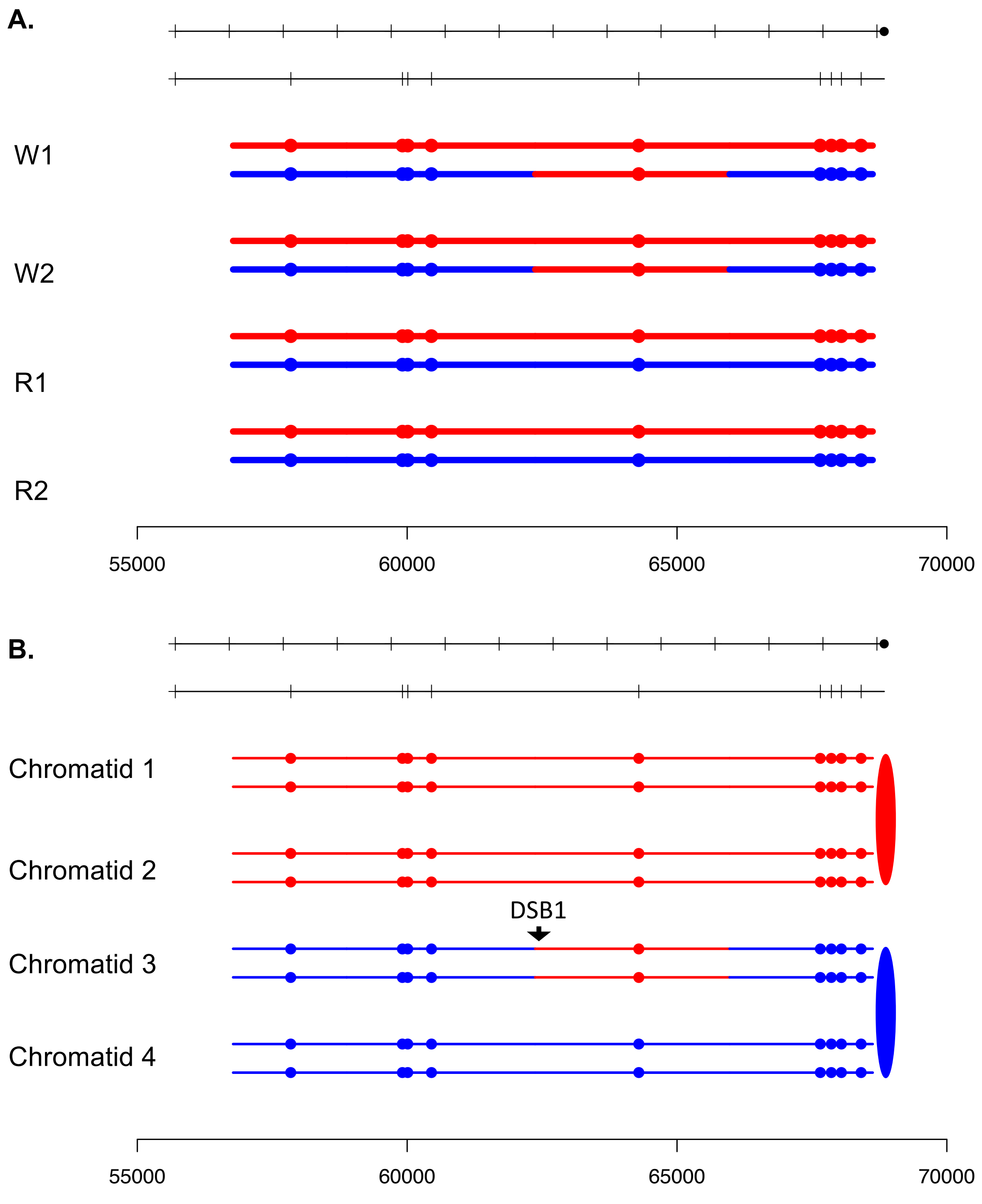
A.
W1
W2
R1
R2
55000
60000
65000
70000
B.
Chromatid 1
Chromatid 2
DSB1
Chromatid 3
Chromatid 4
55000
60000
65000
70000

S5

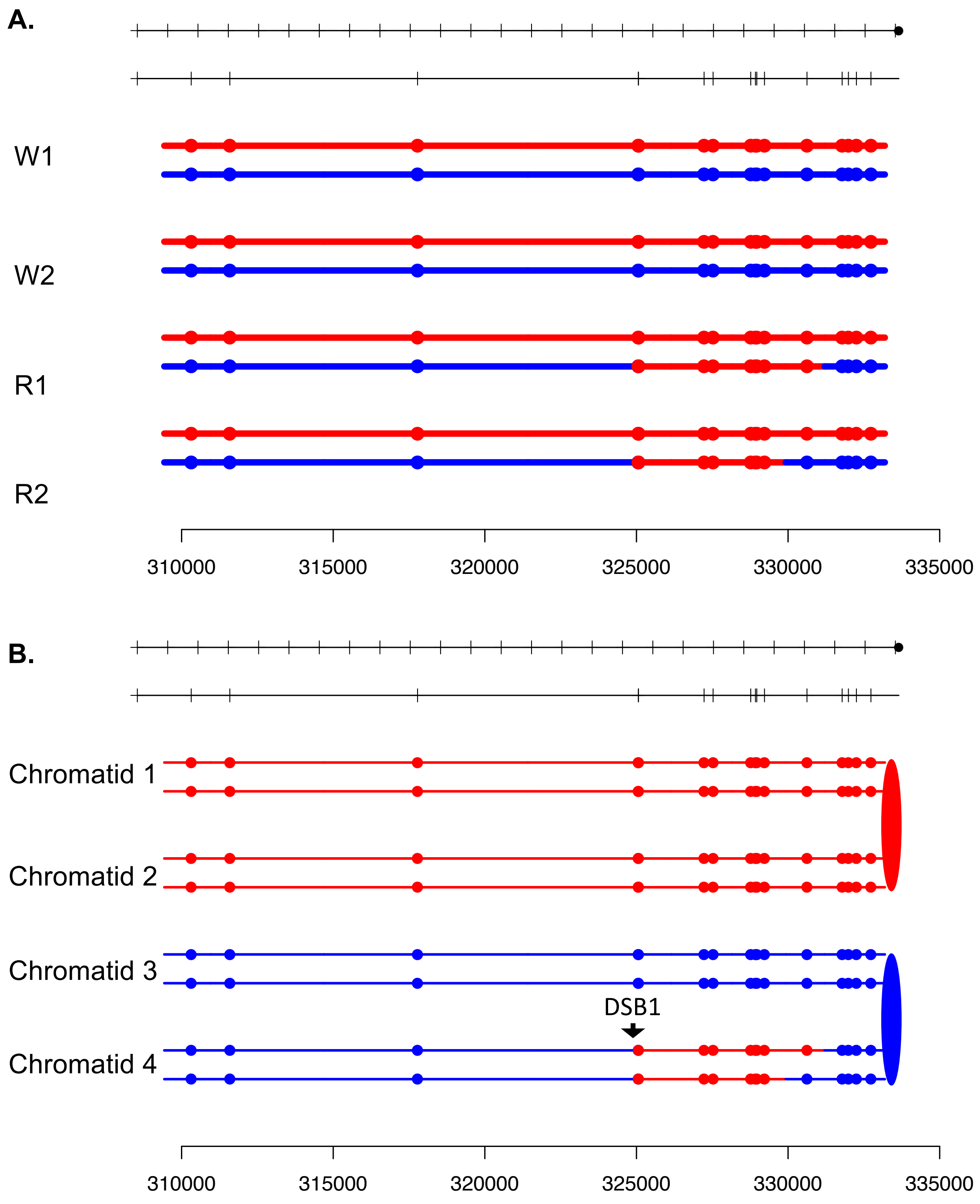
A.
W1
W2
R1
R2
310000
315000
320000
325000
330000
335000
B.
Chromatid 1
Chromatid 2
Chromatid 3
Chromatid 4
DSB1
310000
315000
320000
325000
330000
335000

S6

A.

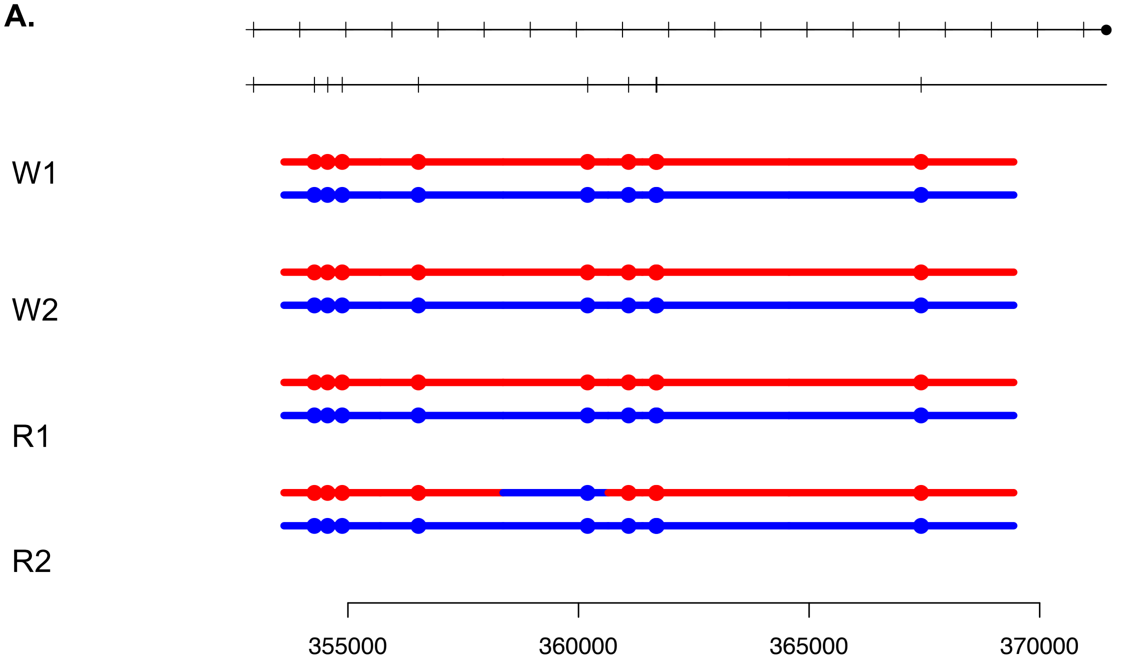
W1
W2
R1
R2
355000
360000
365000
370000

B.

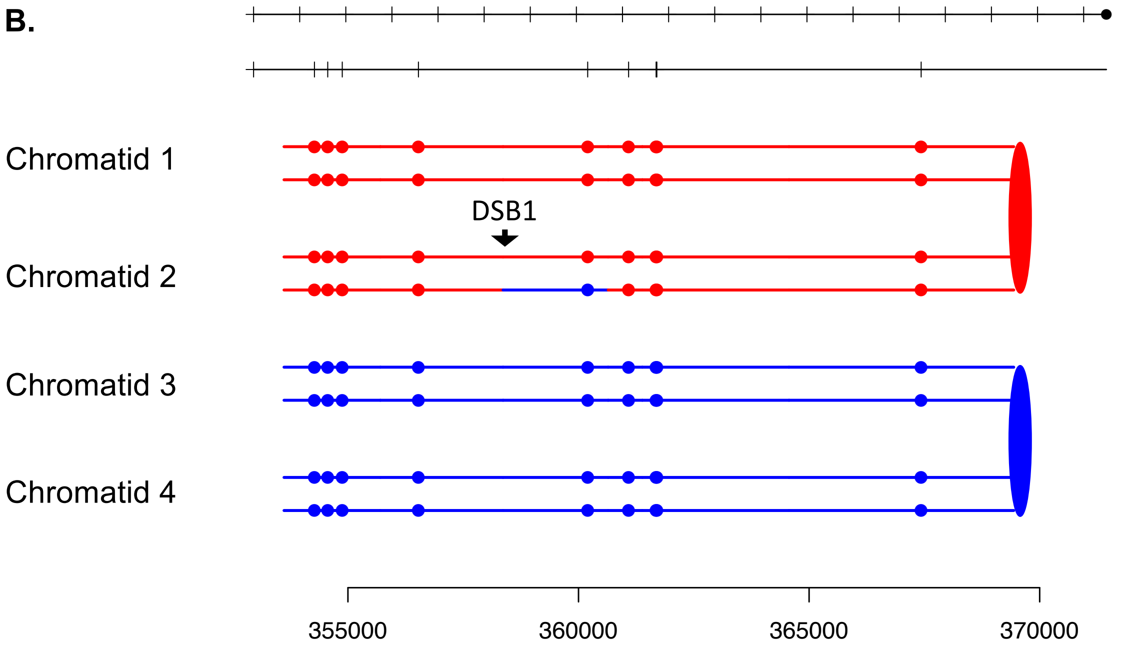
Chromatid 1
DSB1
Chromatid 2
Chromatid 3
Chromatid 4
355000
360000
365000
370000

S7

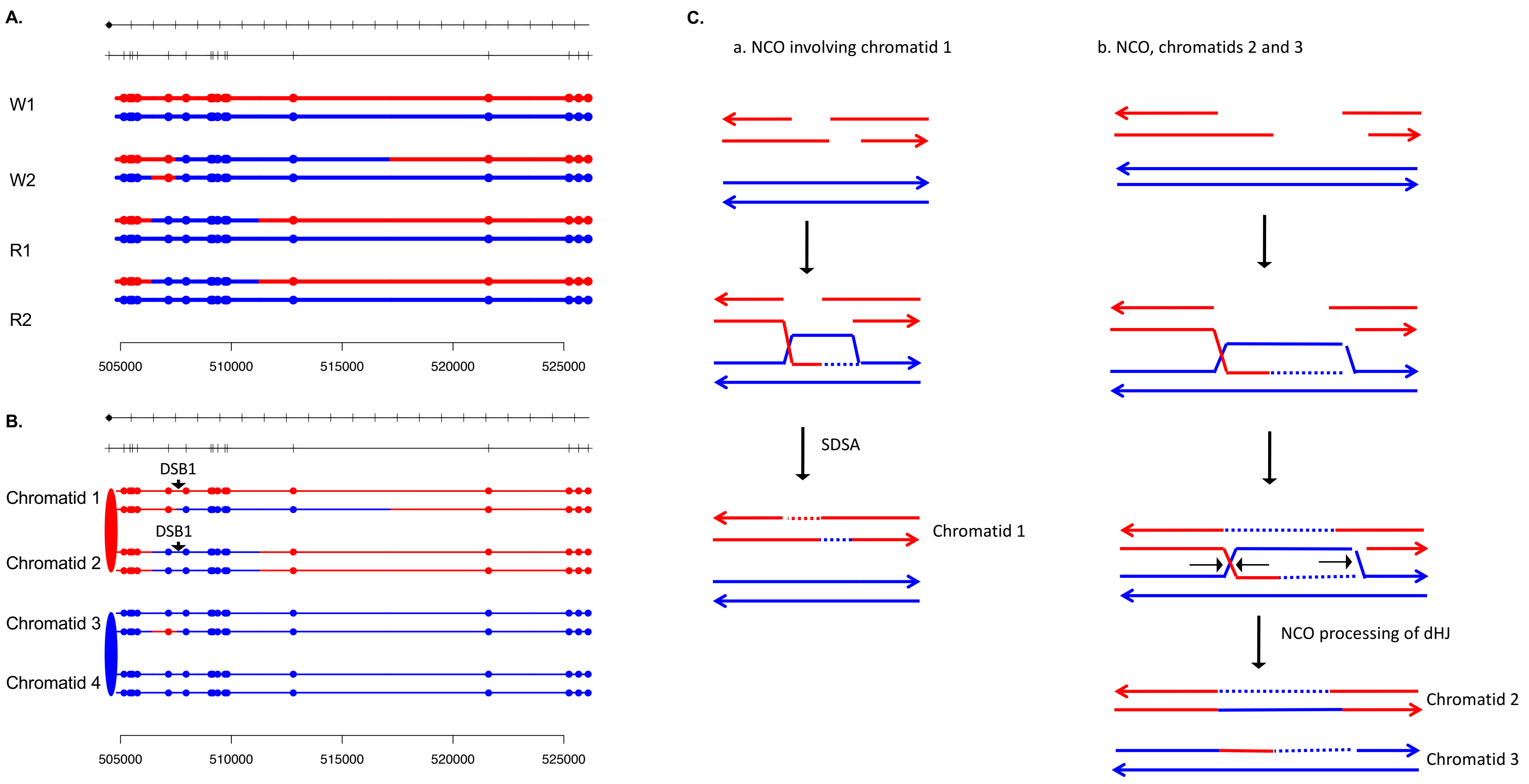

S7	NCO1	Simple heteroduplex on chromatid 1	SDSA
	NCO2	Large homoduplex region separating DSB site from heteroduplex tract (chromatids 2 and 3).	Repair of double-stranded DNA gap

S8

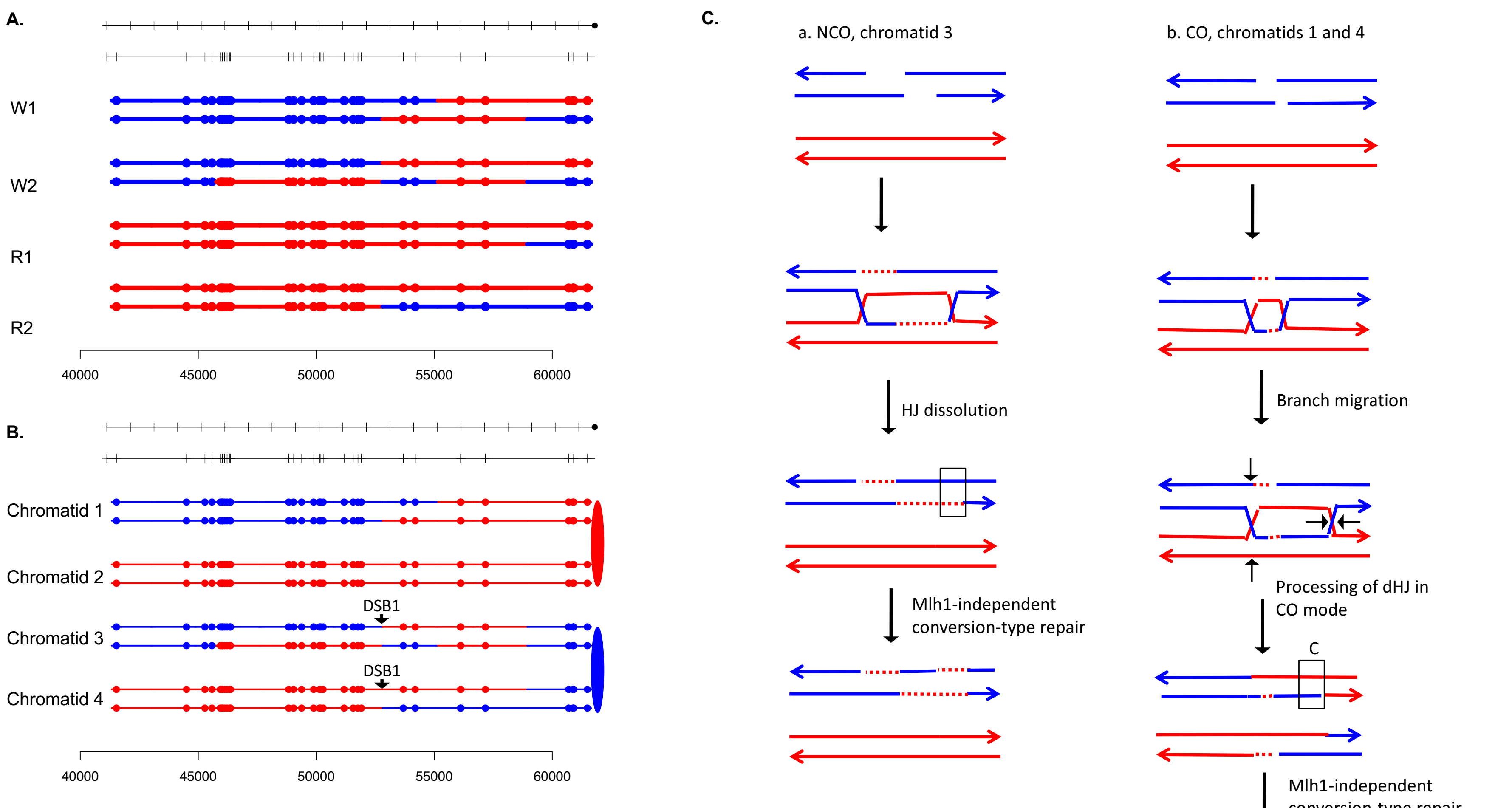

S8	CO	Symmetric heteroduplexes	Branch migration following formation of double Holliday junction (dHJ)
	NCO	Strand switches of heteroduplexes on NCO chromatid, region of conversion at junction of heteroduplex	dHJ dissolution and region of Mlh1-independent MMR

S9

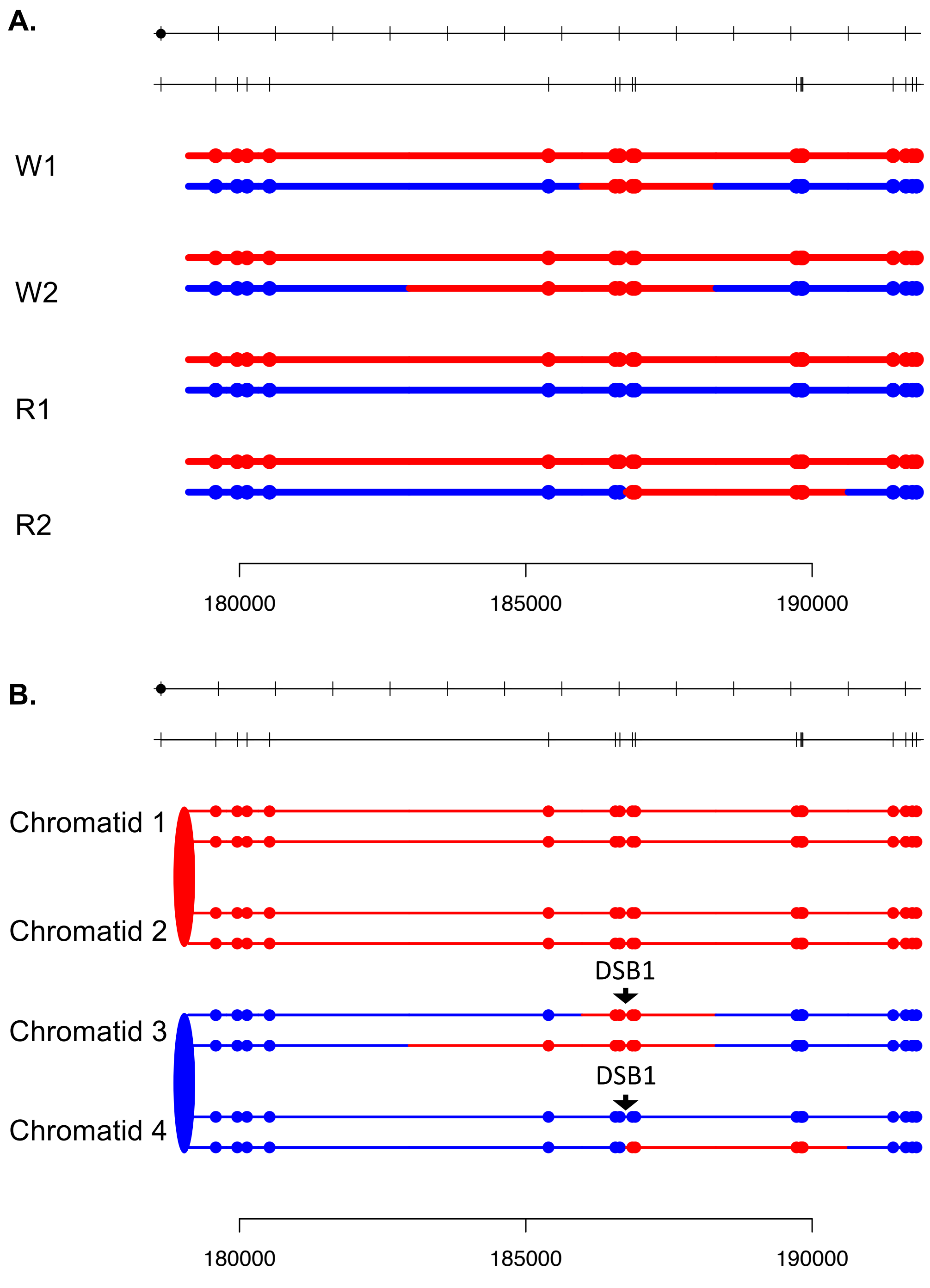
A.
W1
W2
R1
R2
180000
185000
190000
B.
Chromatid 1
Chromatid 2
Chromatid 3
Chromatid 4
DSB1
DSB1
180000
185000
190000

S10

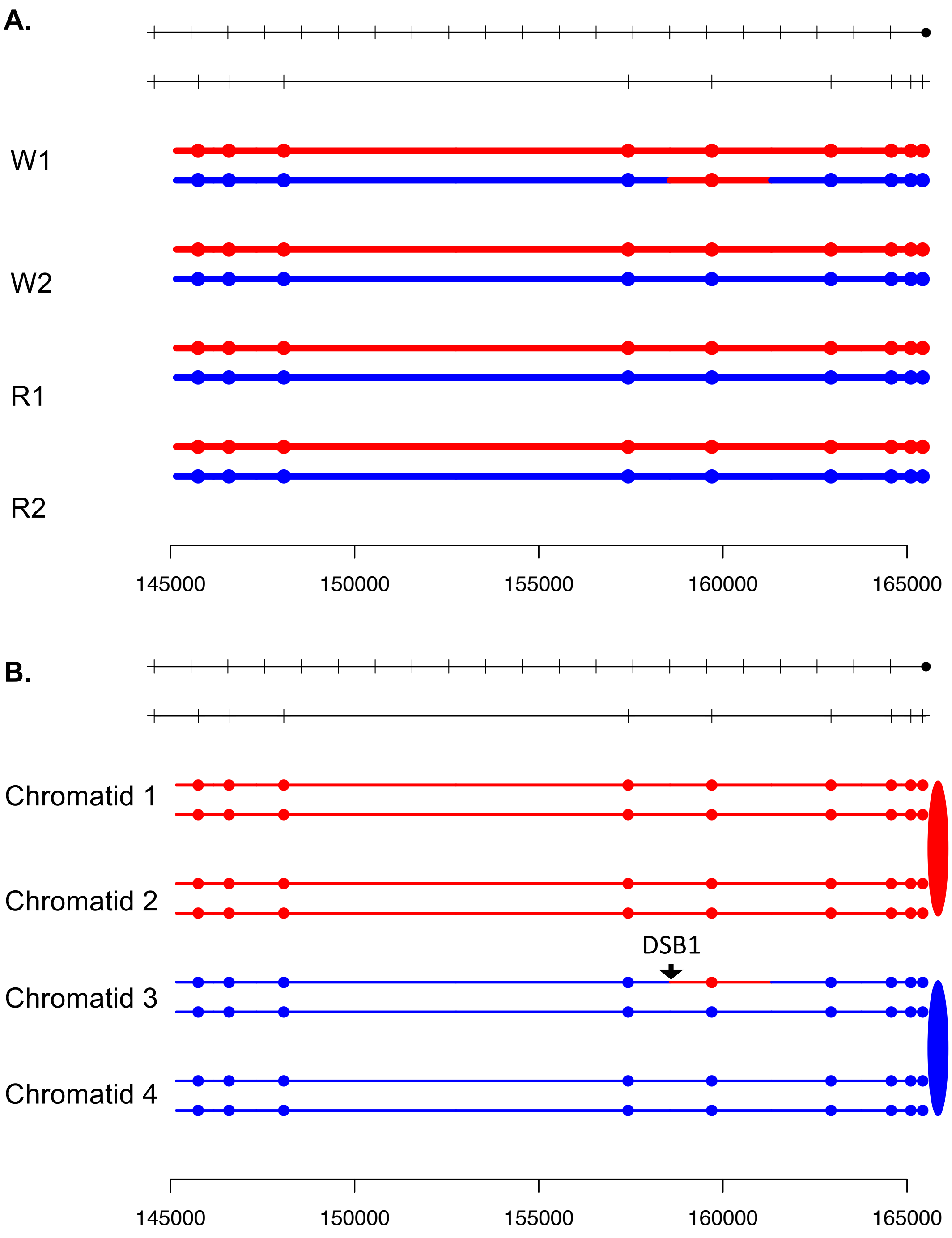
A.
W1
W2
R1
R2
145000
150000
155000
160000
165000
B.
Chromatid 1
Chromatid 2
DSB1
Chromatid 3
Chromatid 4
145000
150000
155000
160000
165000

S11

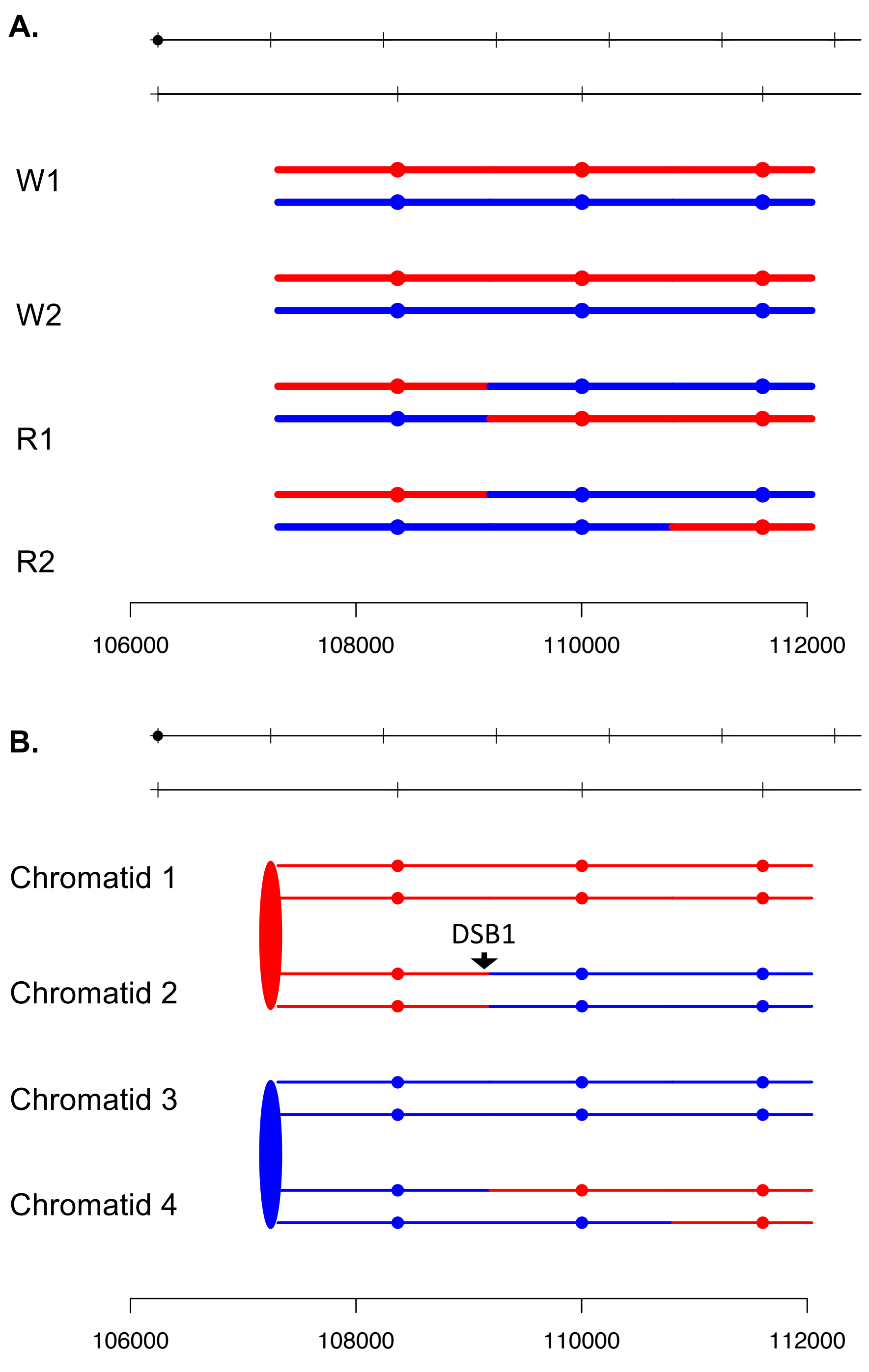
A.
W1
W2
R1
R2
106000
108000
110000
112000
B.
Chromatid 1
Chromatid 2
Chromatid 3
Chromatid 4
DSB1
106000
108000
110000
112000

S12

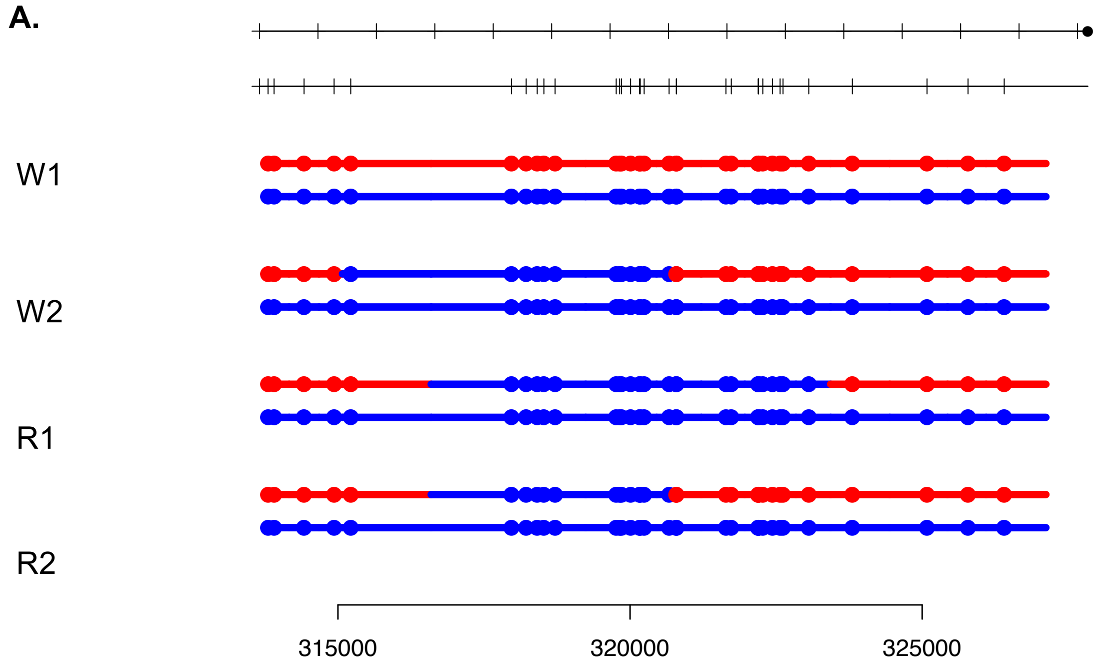
A.
W1
W2
R1
R2
315000
320000
325000

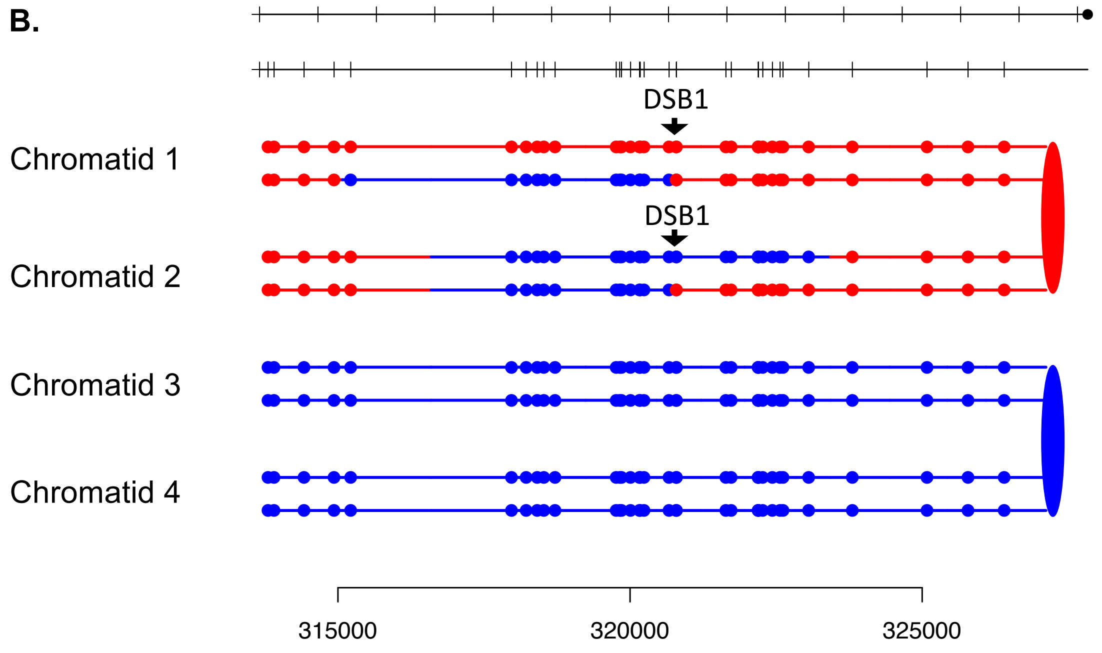
B.
DSB1
Chromatid 1
DSB1
Chromatid 2
Chromatid 3
Chromatid 4
315000
320000
325000

S13

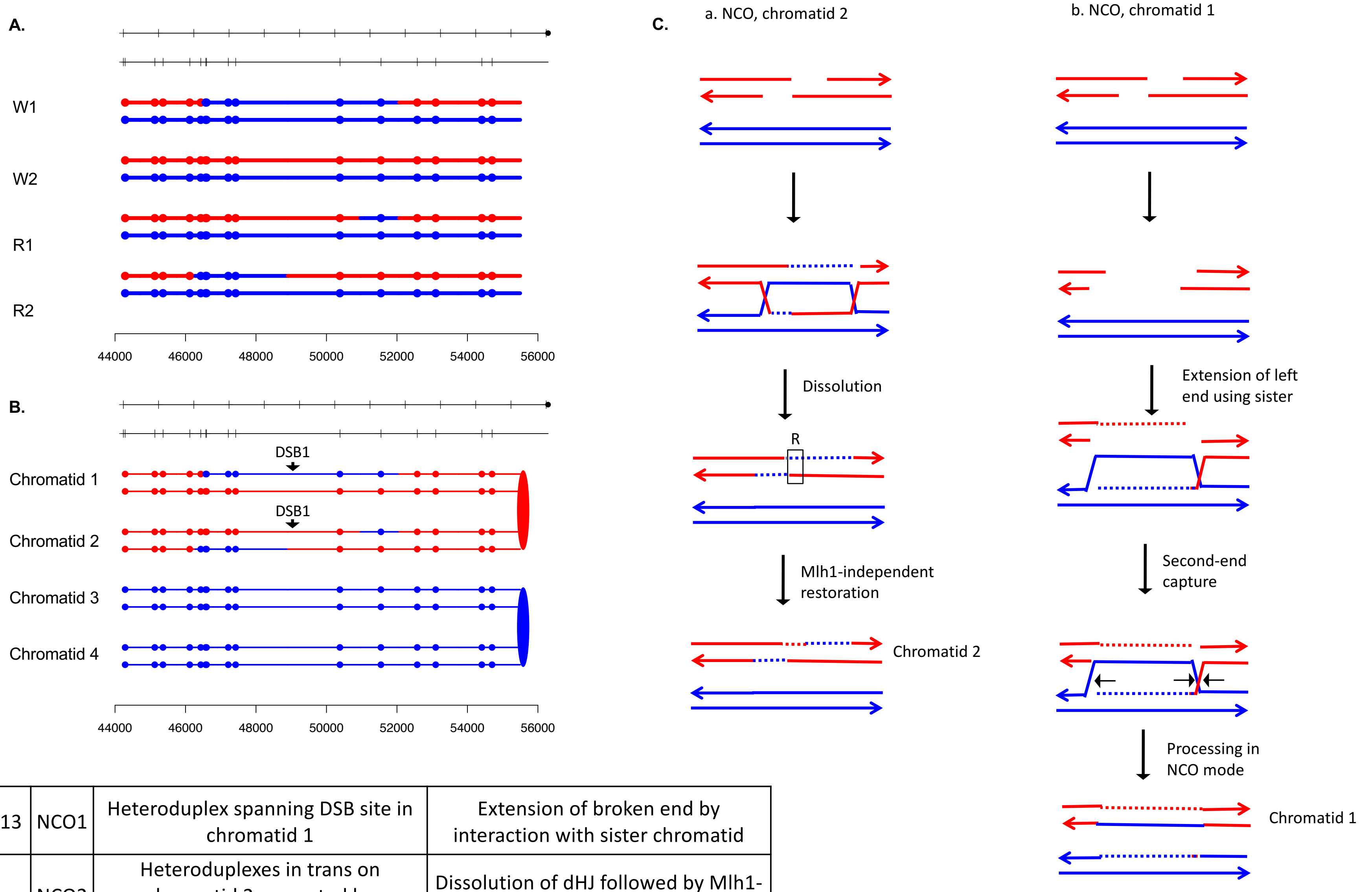

S13	NCO1	Heteroduplex spanning DSB site in chromatid 1	Extension of broken end by interaction with sister chromatid
	NCO2	Heteroduplexes in trans on chromatid 2 separated by homoduplex	Dissolution of dHJ followed by Mlh1-independent MMR

S14

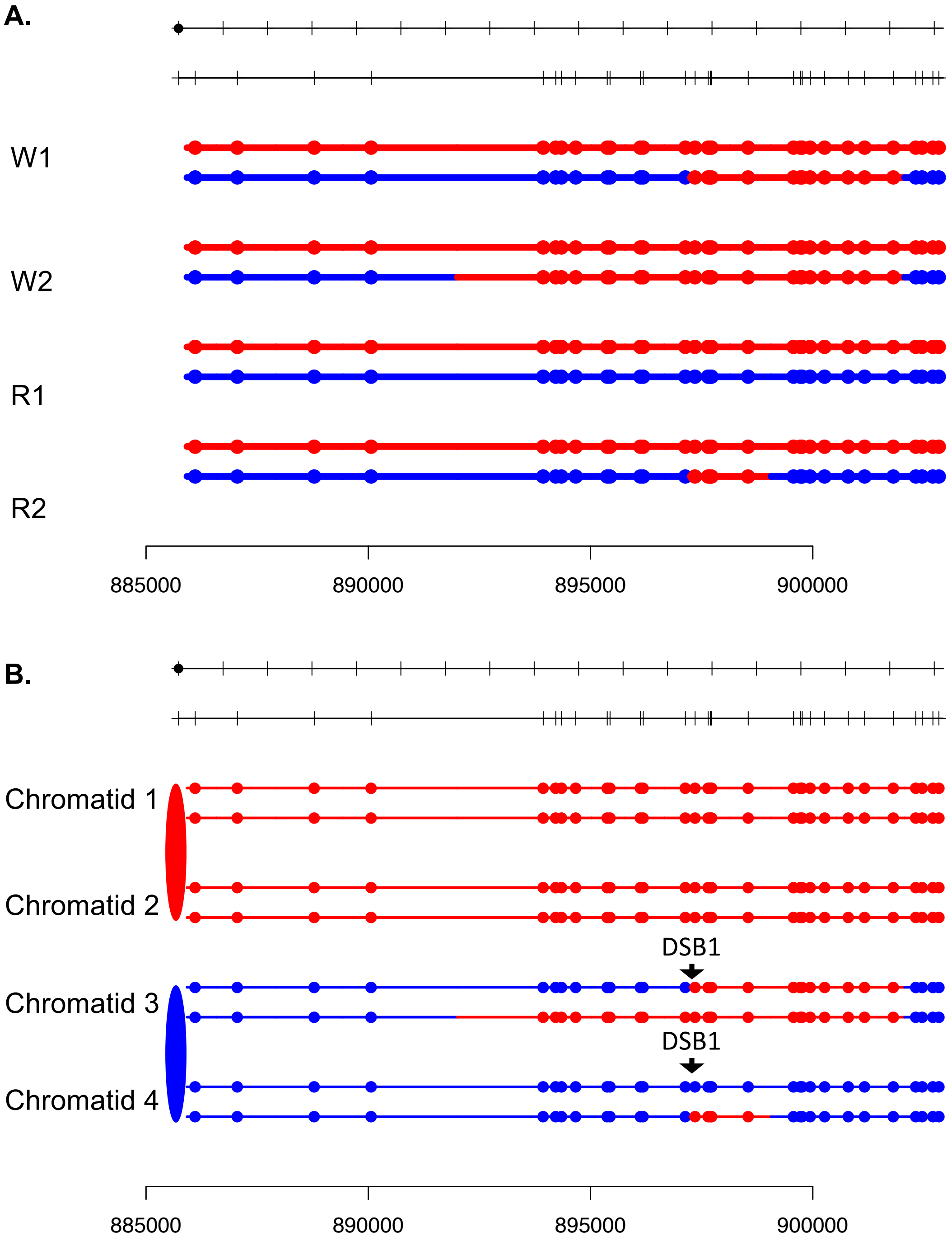
A.
W1
W2
R1
R2
885000
890000
895000
900000
B.
Chromatid 1
Chromatid 2
Chromatid 3
Chromatid 4
DSB1
DSB1
885000
890000
895000
900000

S15

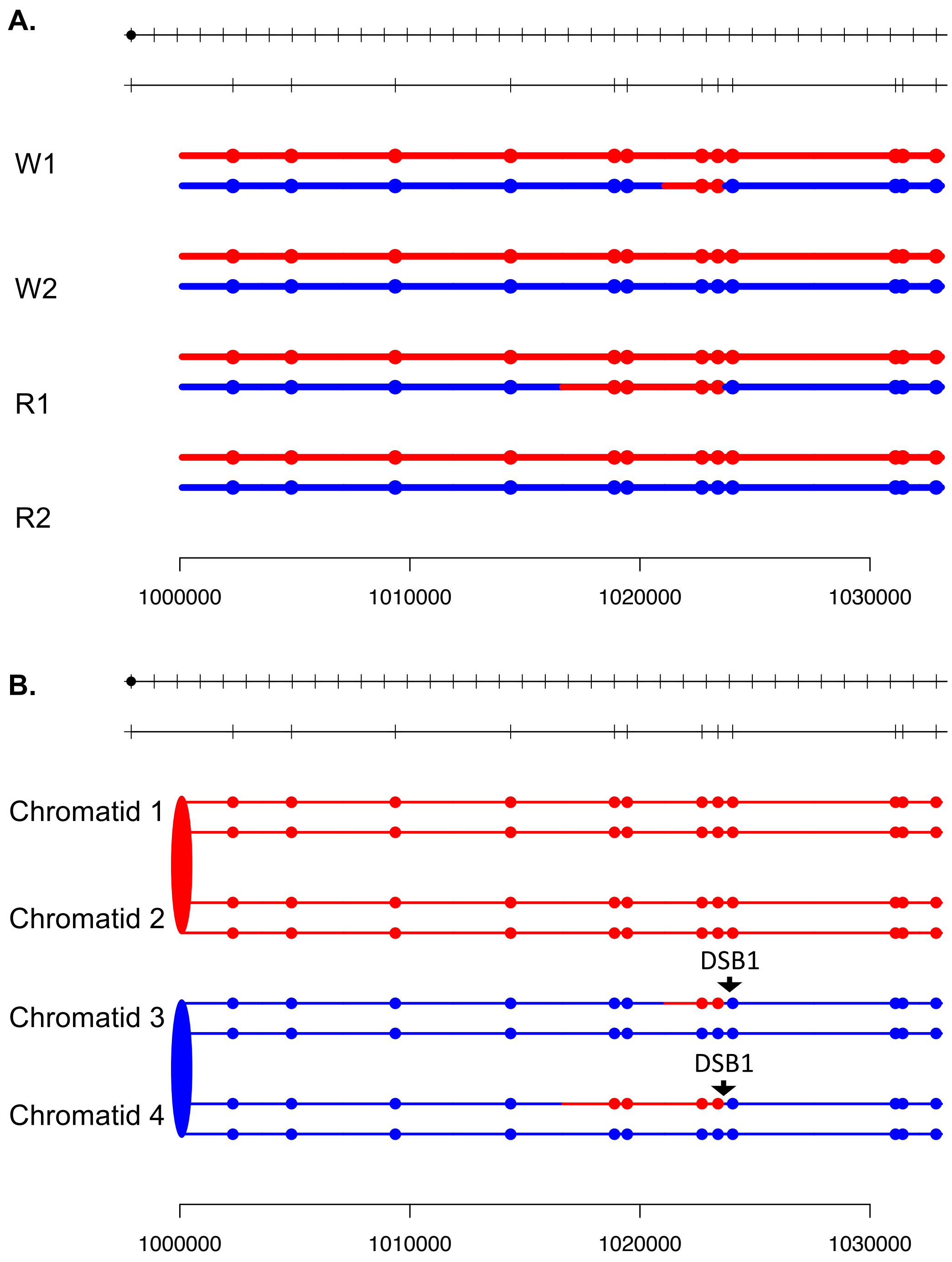
A.
W1
W2
R1
R2
1000000
1010000
1020000
1030000
B.
Chromatid 1
Chromatid 2
DSB1
Chromatid 3
DSB1
Chromatid 4
1000000
1010000
1020000
1030000

S16

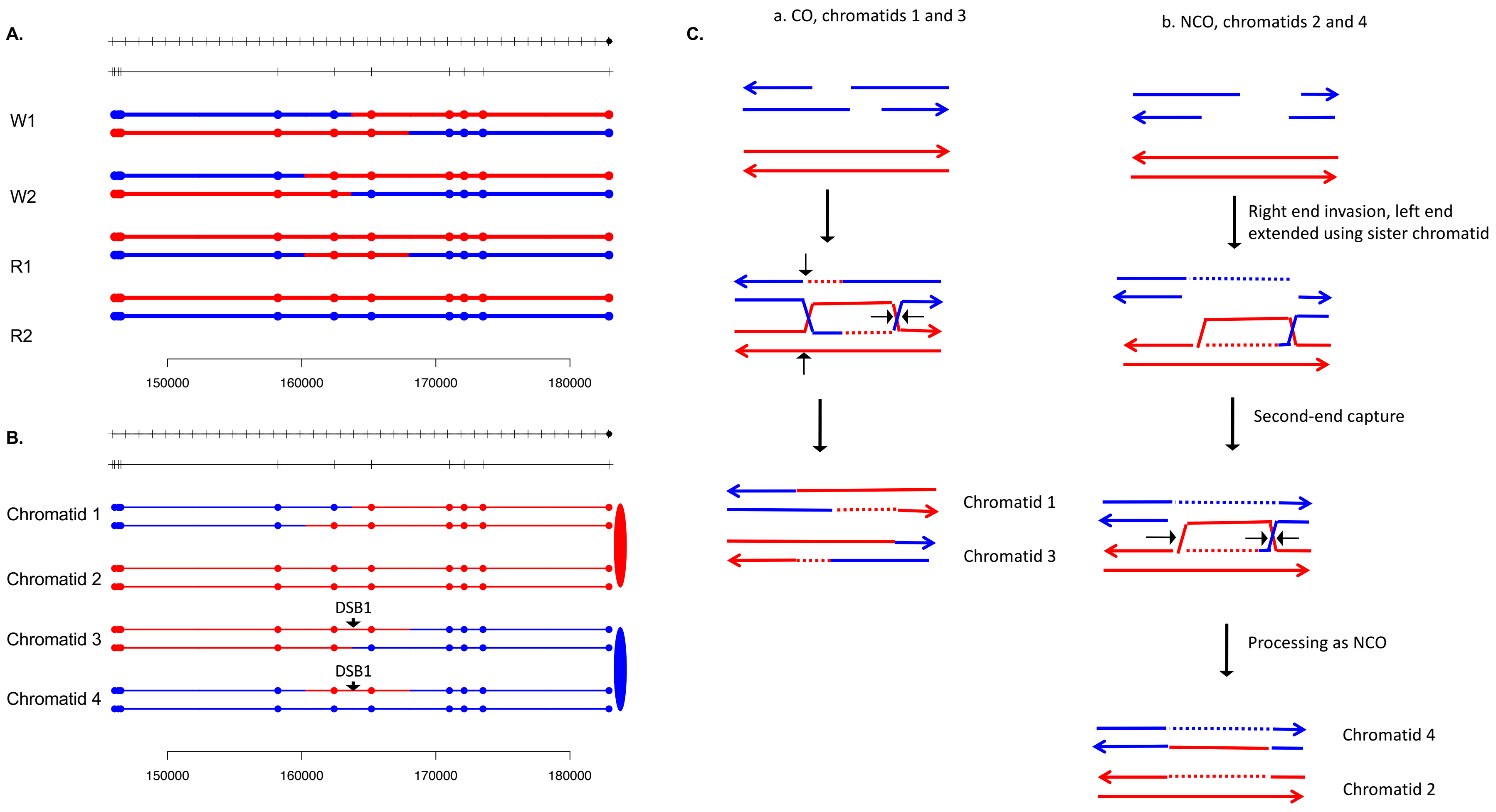

S16	CO	Heteroduplexes in *trans* flanking DSB site	Standard CO according to DSBR model
	NCO	Heteroduplex spanning DSB site on NCO chromatid 4	Extension of broken end by interaction with sister chromatid

S17

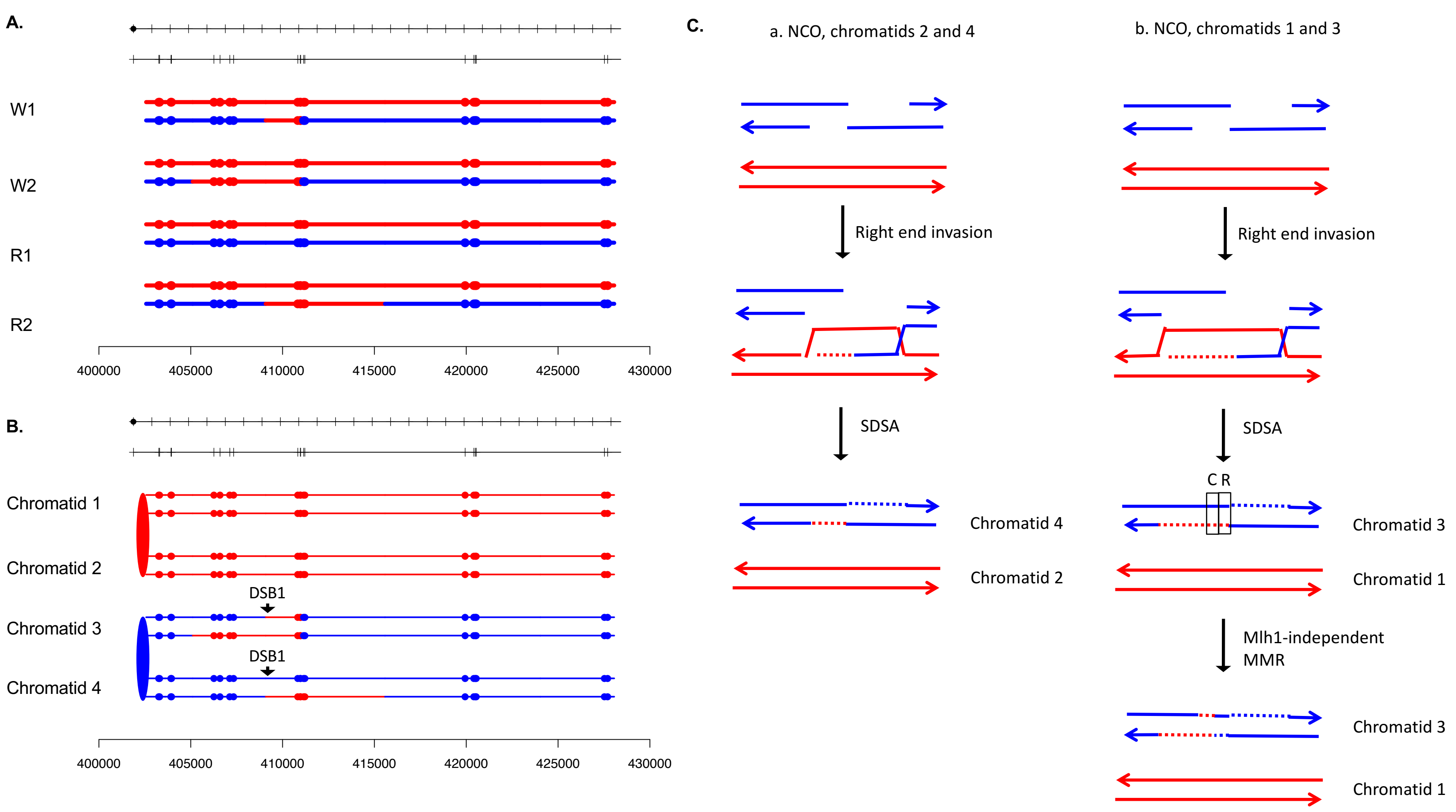

S17	NCO1	Heteroduplex with interspersed conversion and restoration tracts (chromatid 3)	SDSA with Mlh1-independent MMR
	NCO2	Simple heteroduplex (chromatid 4)	SDSA

S18

A.

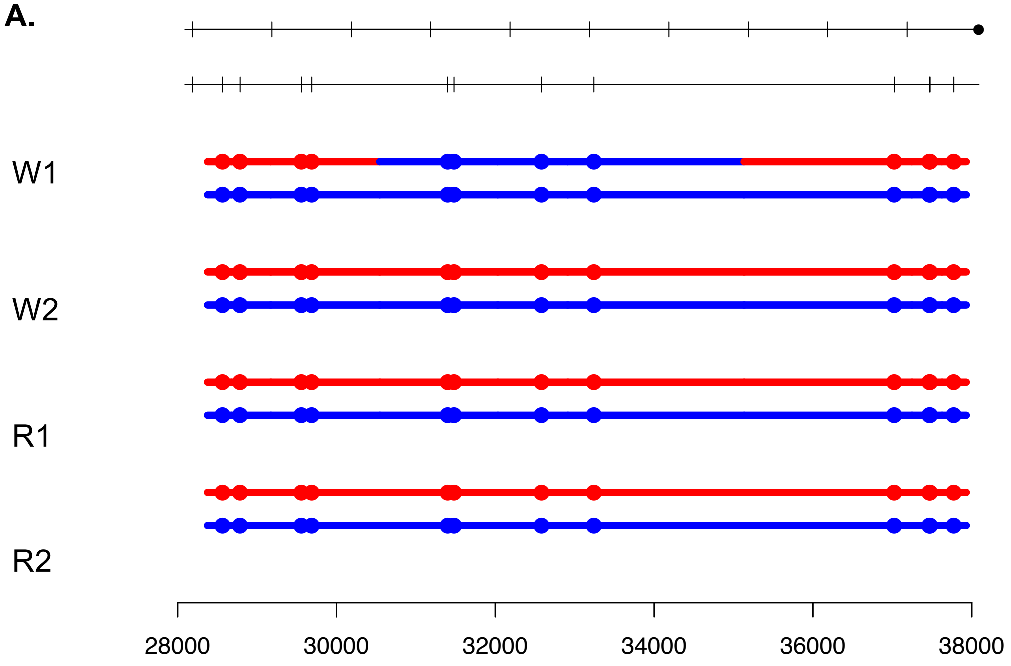

W1
W2
R1
R2
28000
30000
32000
34000
36000
38000

B.

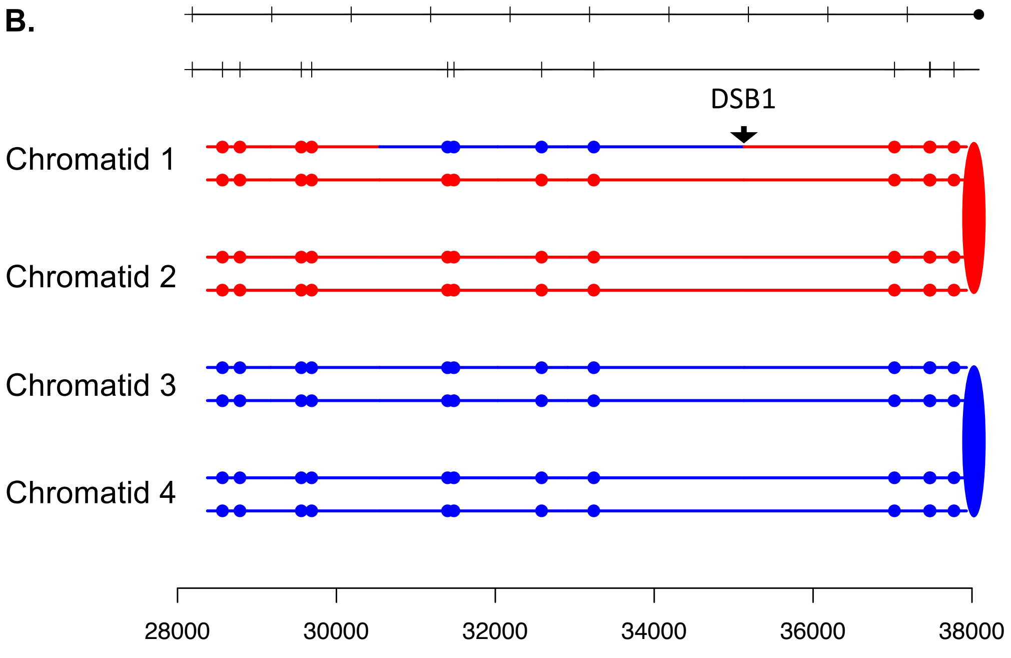

DSB1
Chromatid 1
Chromatid 2
Chromatid 3
Chromatid 4
28000
30000
32000
34000
36000
38000

S19

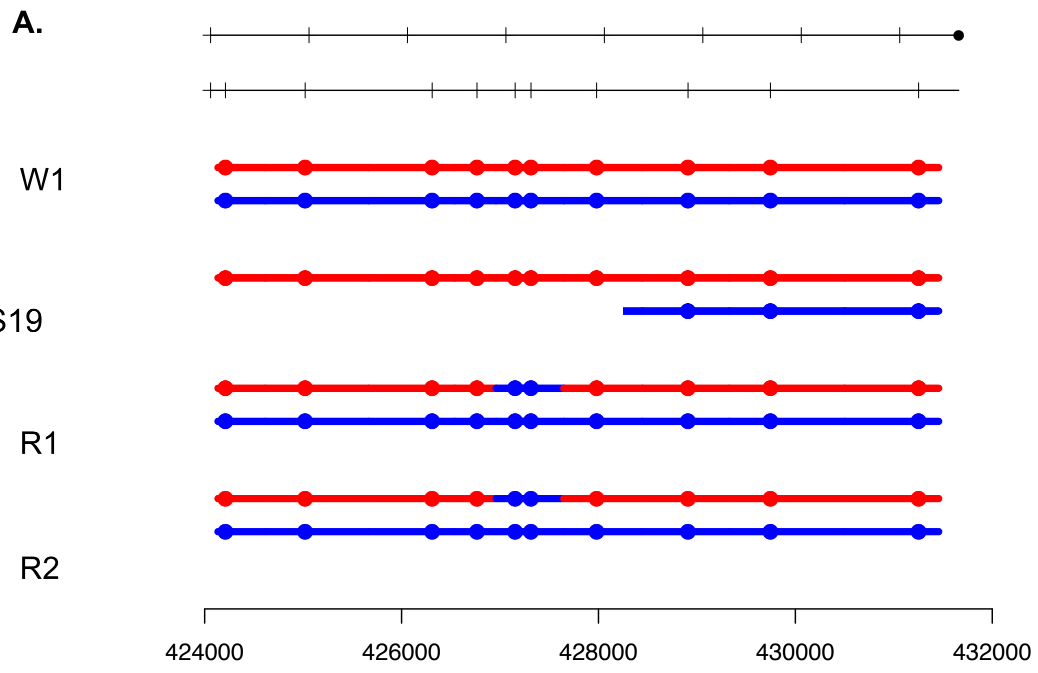
A.
W1
S19
R1
R2
424000
426000
428000
430000
432000

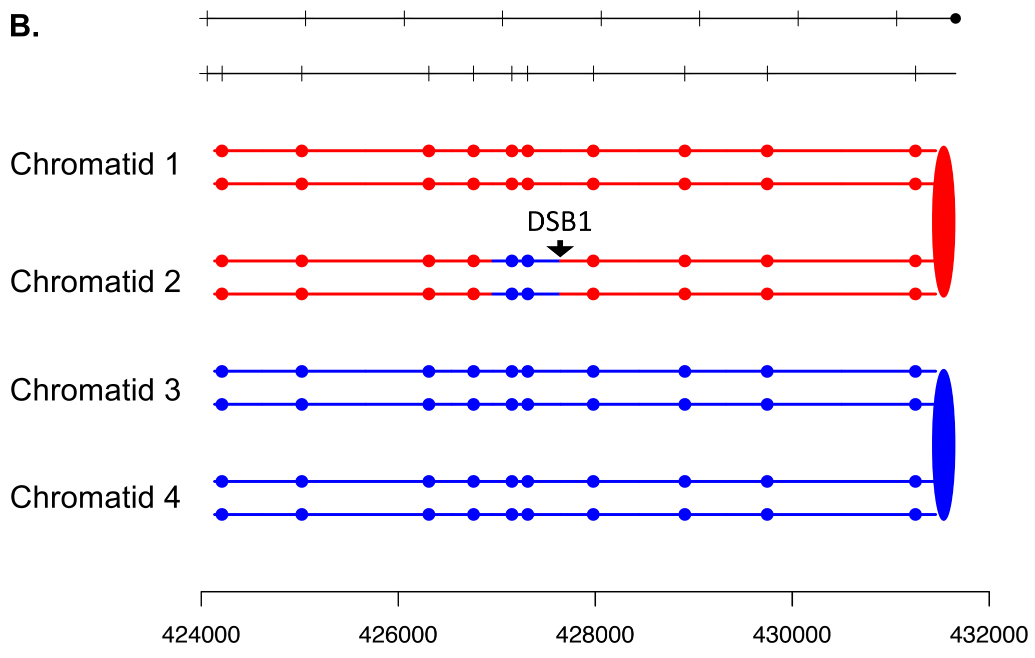
B.
Chromatid 1
DSB1
Chromatid 2
Chromatid 3
Chromatid 4
424000
426000
428000
430000
432000

S20

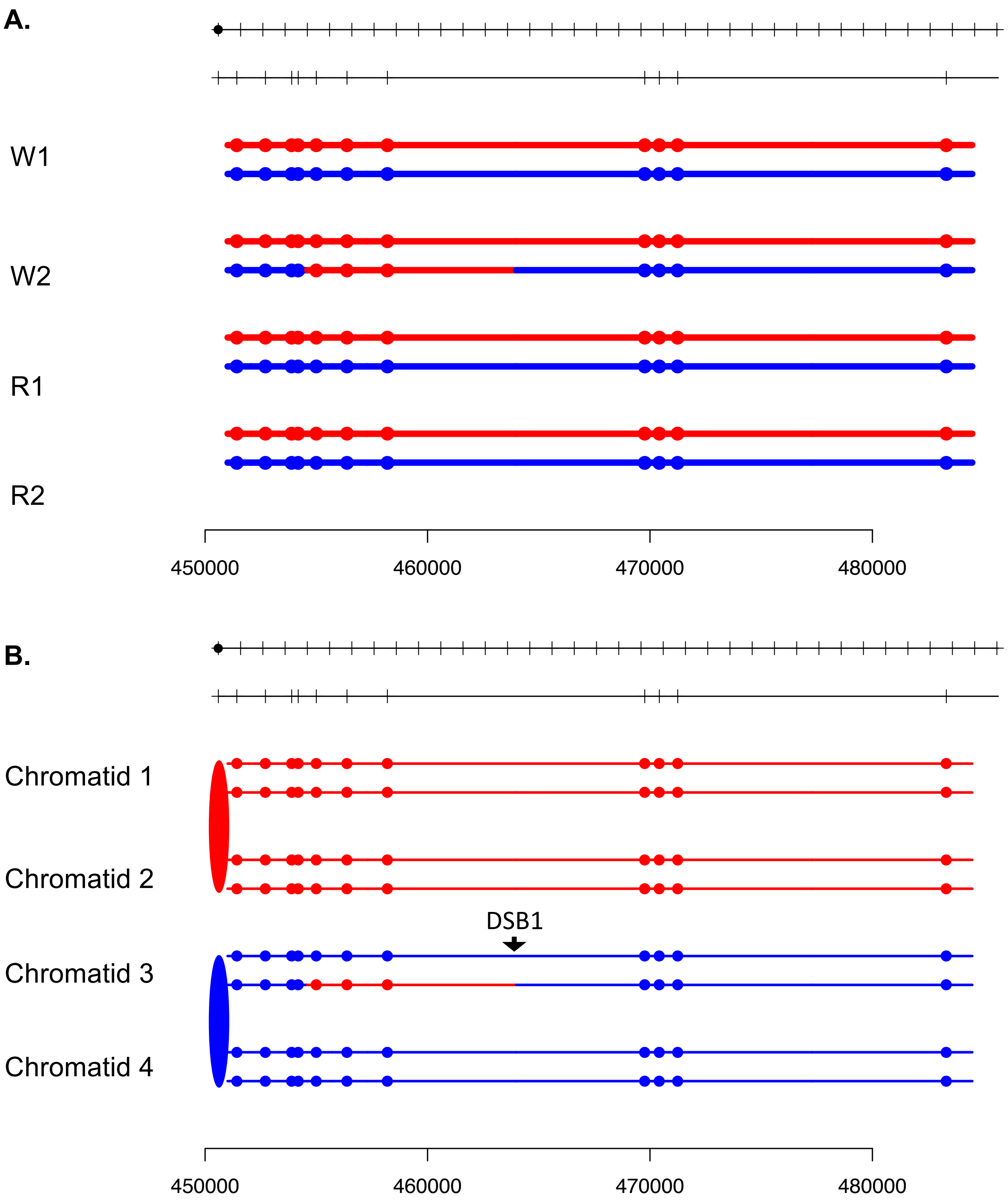
A.
W1
W2
R1
R2
450000
460000
470000
480000
B.
Chromatid 1
Chromatid 2
DSB1
Chromatid 3
Chromatid 4
450000
460000
470000
480000

S21

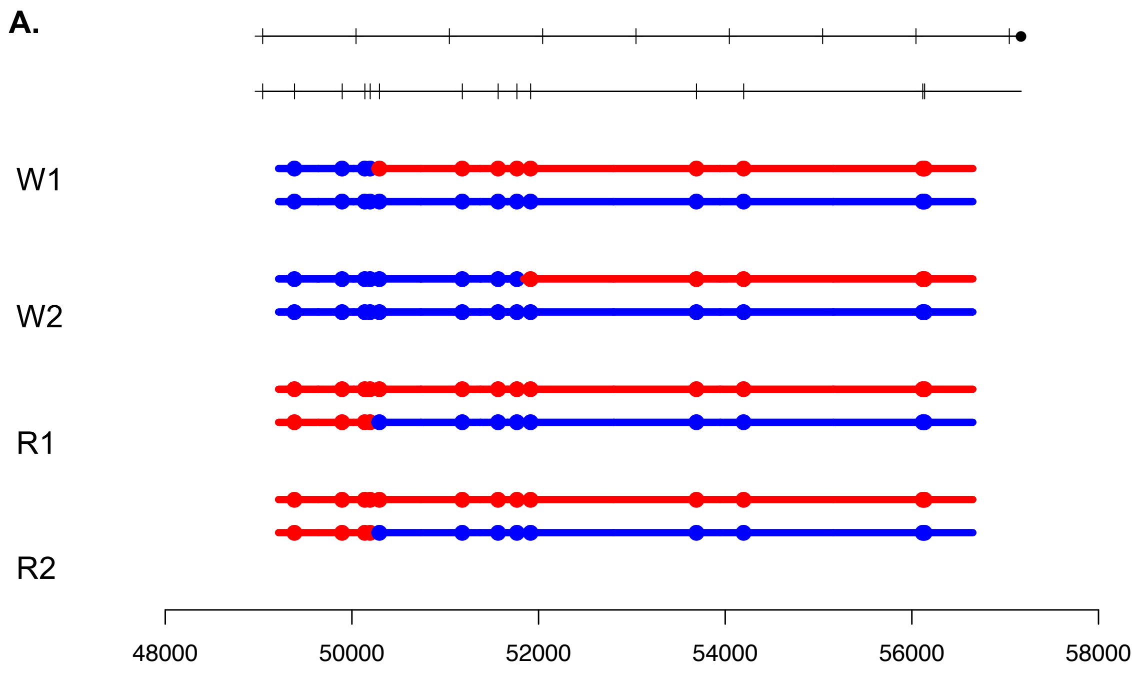
A.
W1
W2
R1
R2
48000
50000
52000
54000
56000
58000

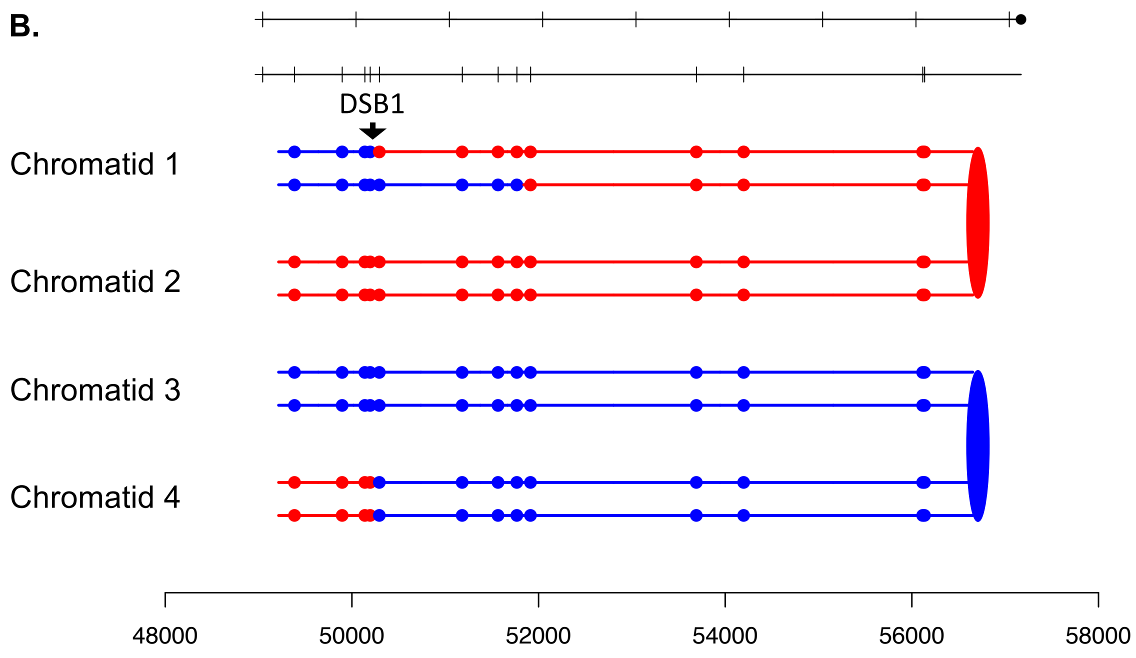
B.
DSB1
Chromatid 1
Chromatid 2
Chromatid 3
Chromatid 4
48000
50000
52000
54000
56000
58000

S22

A.

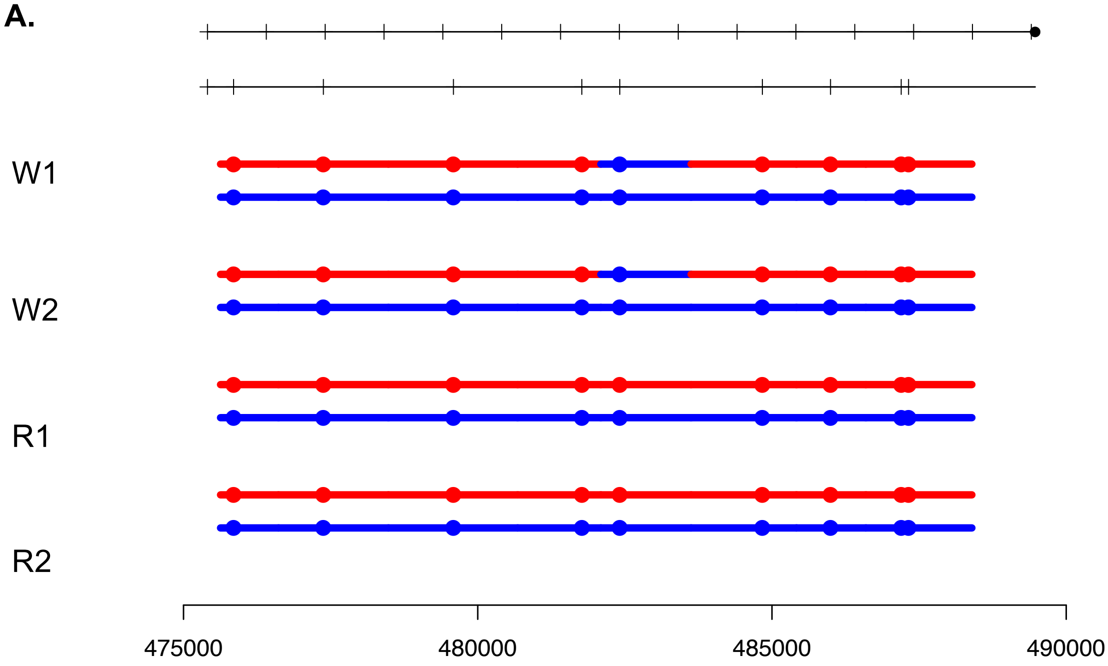
W1
W2
R1
R2
475000
480000
485000
490000

B.

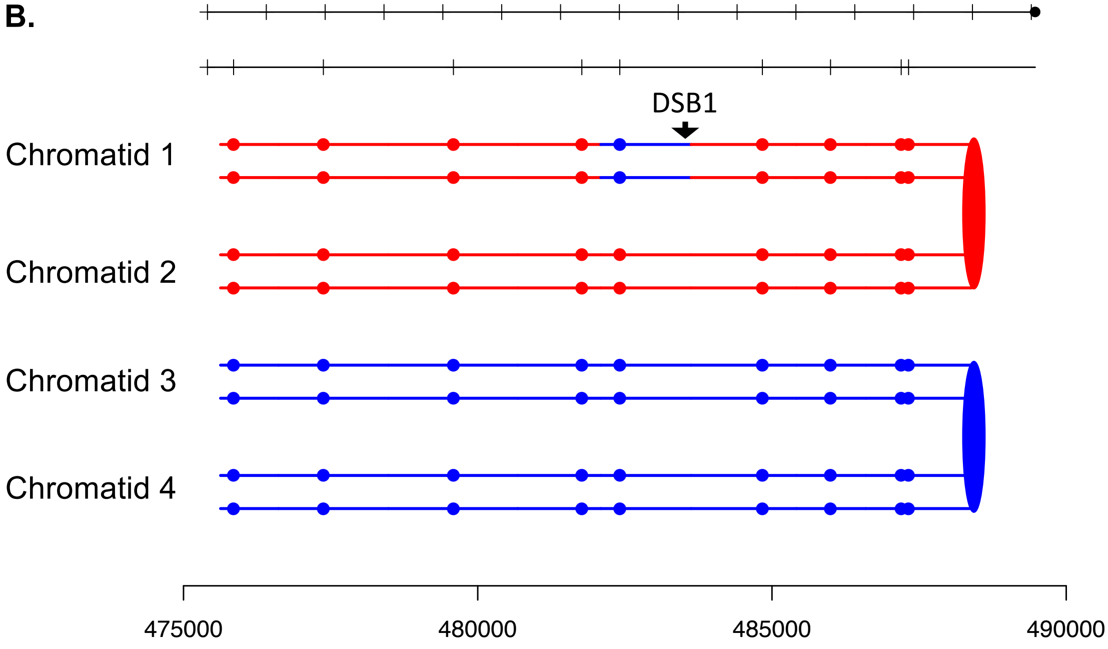
DSB1
Chromatid 1
Chromatid 2
Chromatid 3
Chromatid 4
475000
480000
485000
490000

S23

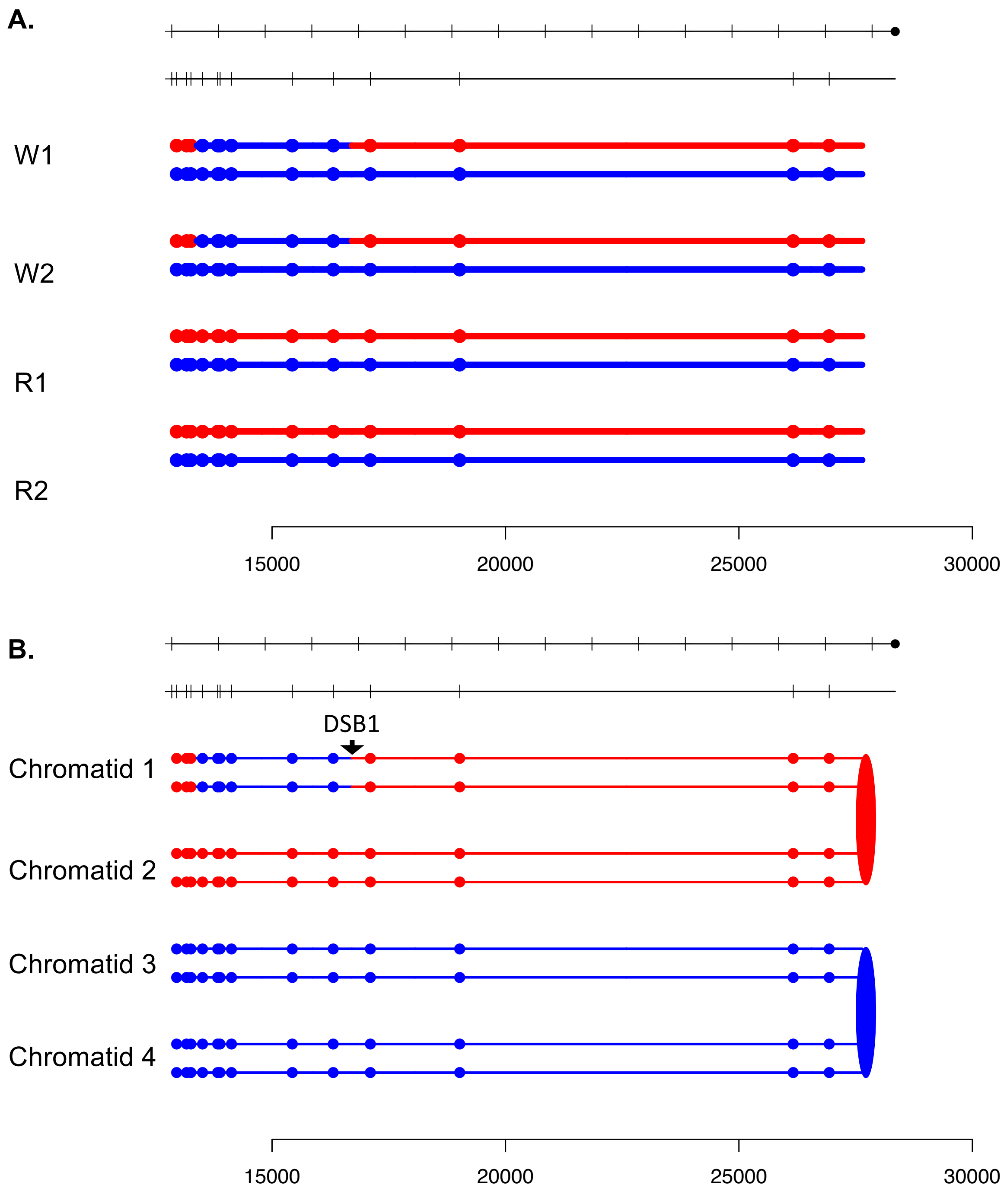
A.
W1
W2
R1
R2
15000
20000
25000
30000
B.
DSB1
Chromatid 1
Chromatid 2
Chromatid 3
Chromatid 4
15000
20000
25000
30000

S24

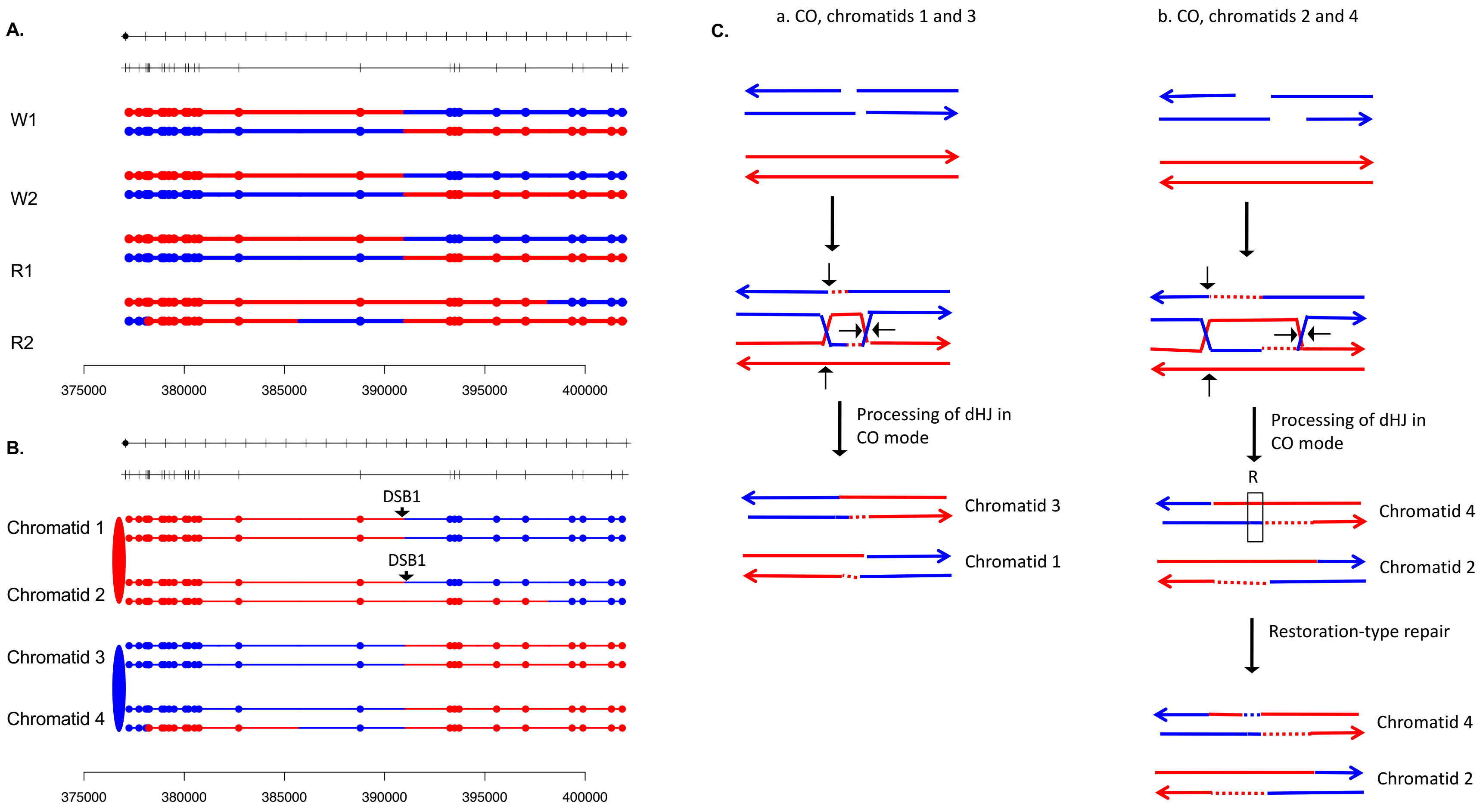

S24	CO1	CO between chromatids 1 and 3 with no heteroduplex	Standard DSBR model with limited strand invasion and limited DNA synthesis
	CO2	CO between chromatids 2 and 4 with restoration tract between *trans* heteroduplexes	Standard DSBR model with tract of Mlh1-independent restoration repair

S25

A.

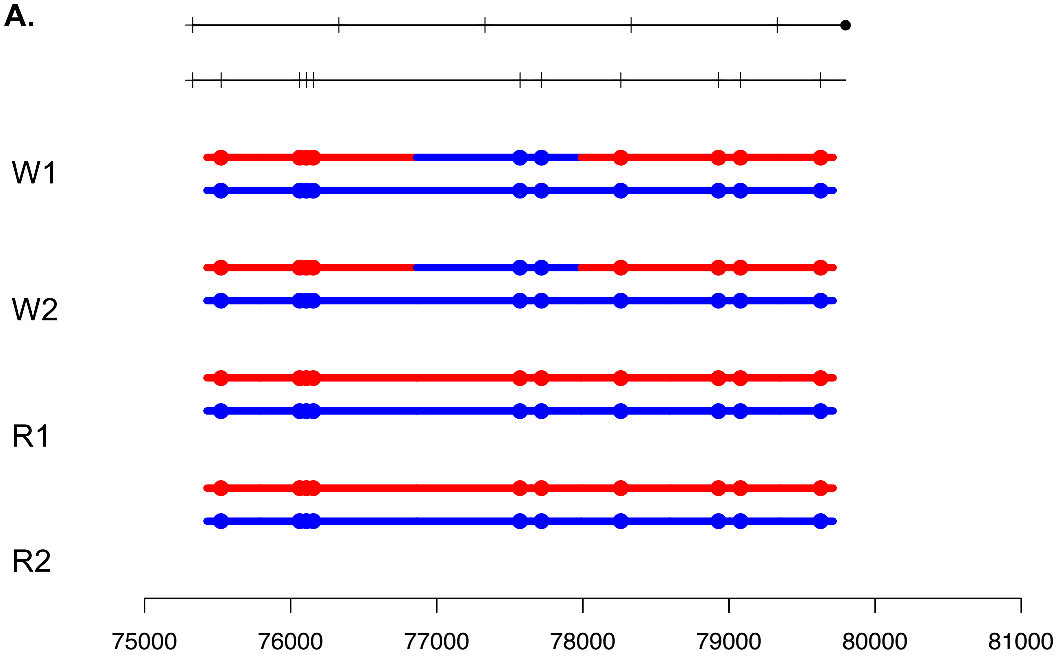
W1
W2
R1
R2
75000
76000
77000
78000
79000
80000
81000

B.

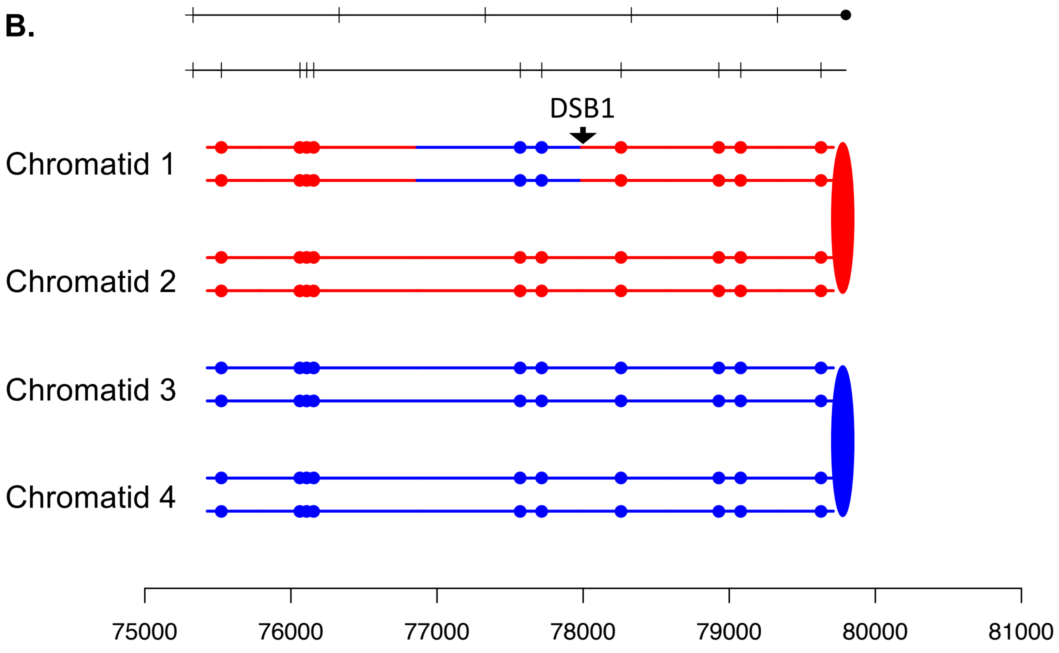
DSB1
Chromatid 1
Chromatid 2
Chromatid 3
Chromatid 4
75000
76000
77000
78000
79000
80000
81000

S26

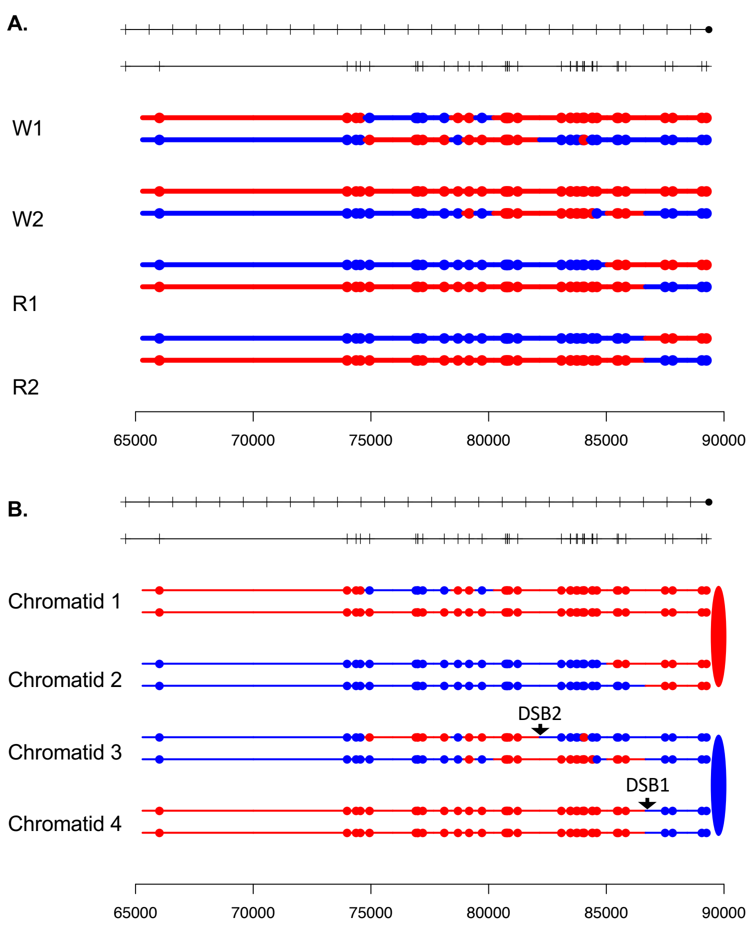

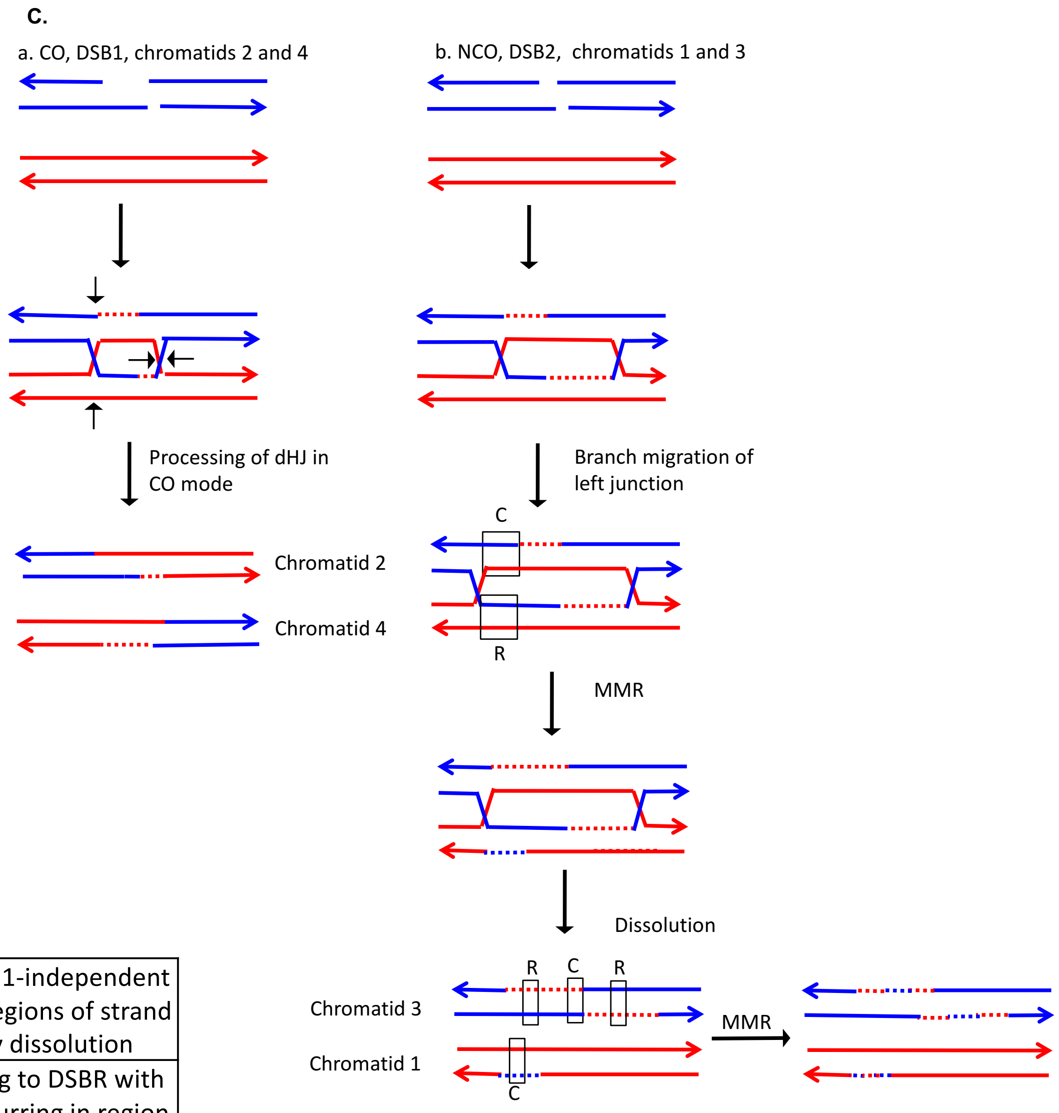

S26	NCO	Symmetric heteroduplexes; regions of homoduplex within heteroduplex; strand switch within heteroduplex	Branch migration; Mlh1-independent MMR; DSB between regions of strand switch, followed by dissolution
	CO	CO between chromatids 2 and 4 with heteroduplex on only one chromatid	Standard CO according to DSBR with one heteroduplex occurring in region without SNP

S27

A.

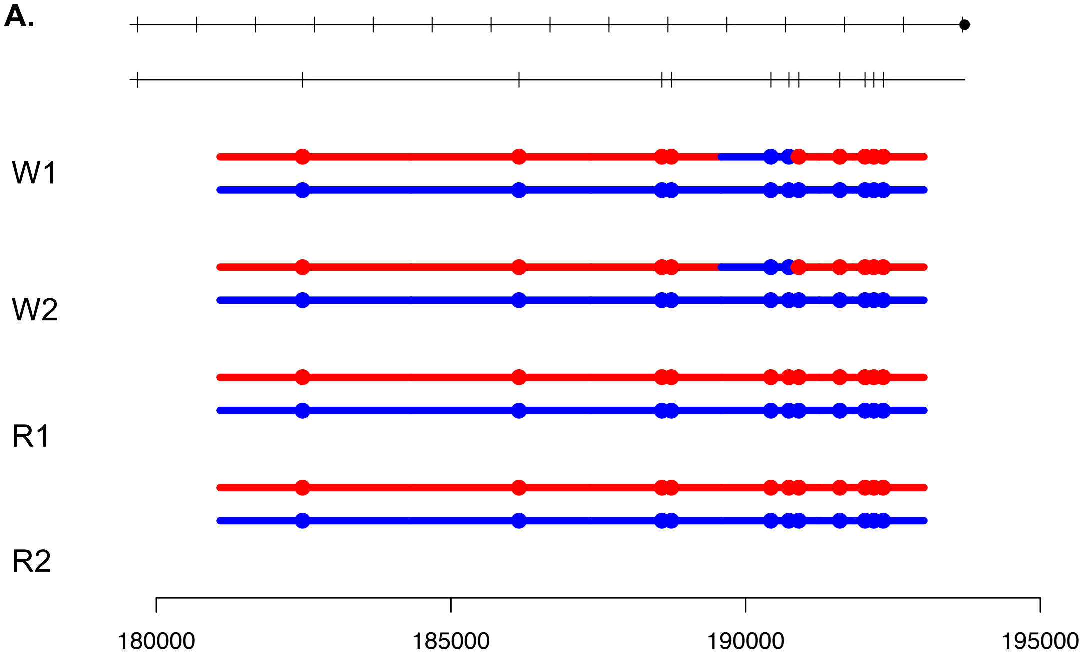
W1
W2
R1
R2
180000
185000
190000
195000

B.

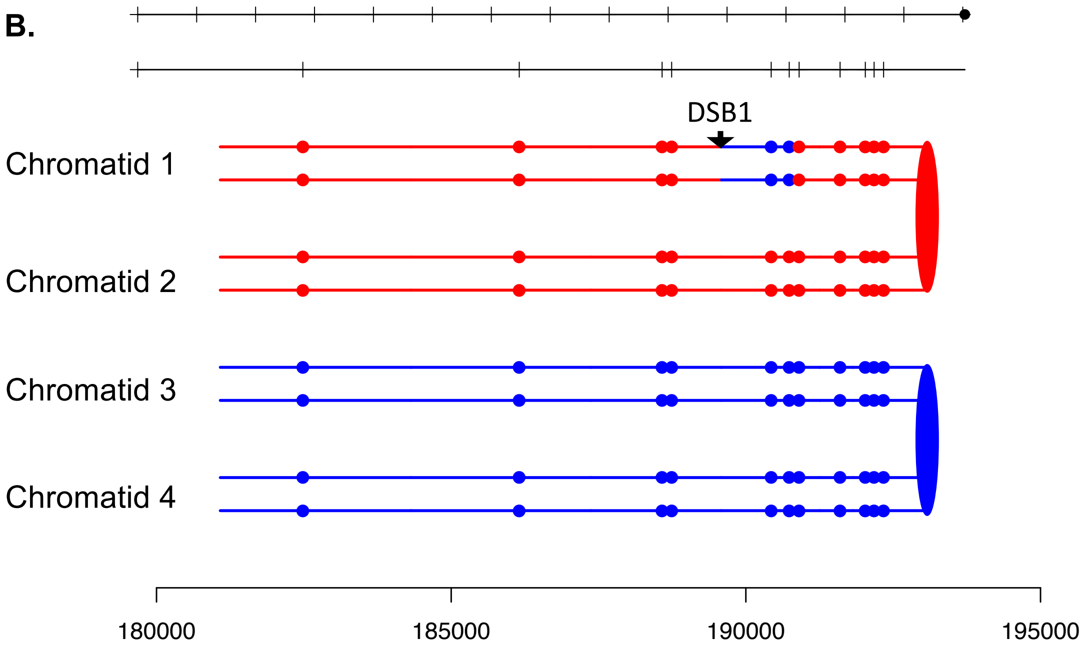
DSB1
Chromatid 1
Chromatid 2
Chromatid 3
Chromatid 4
180000
185000
190000
195000

S28

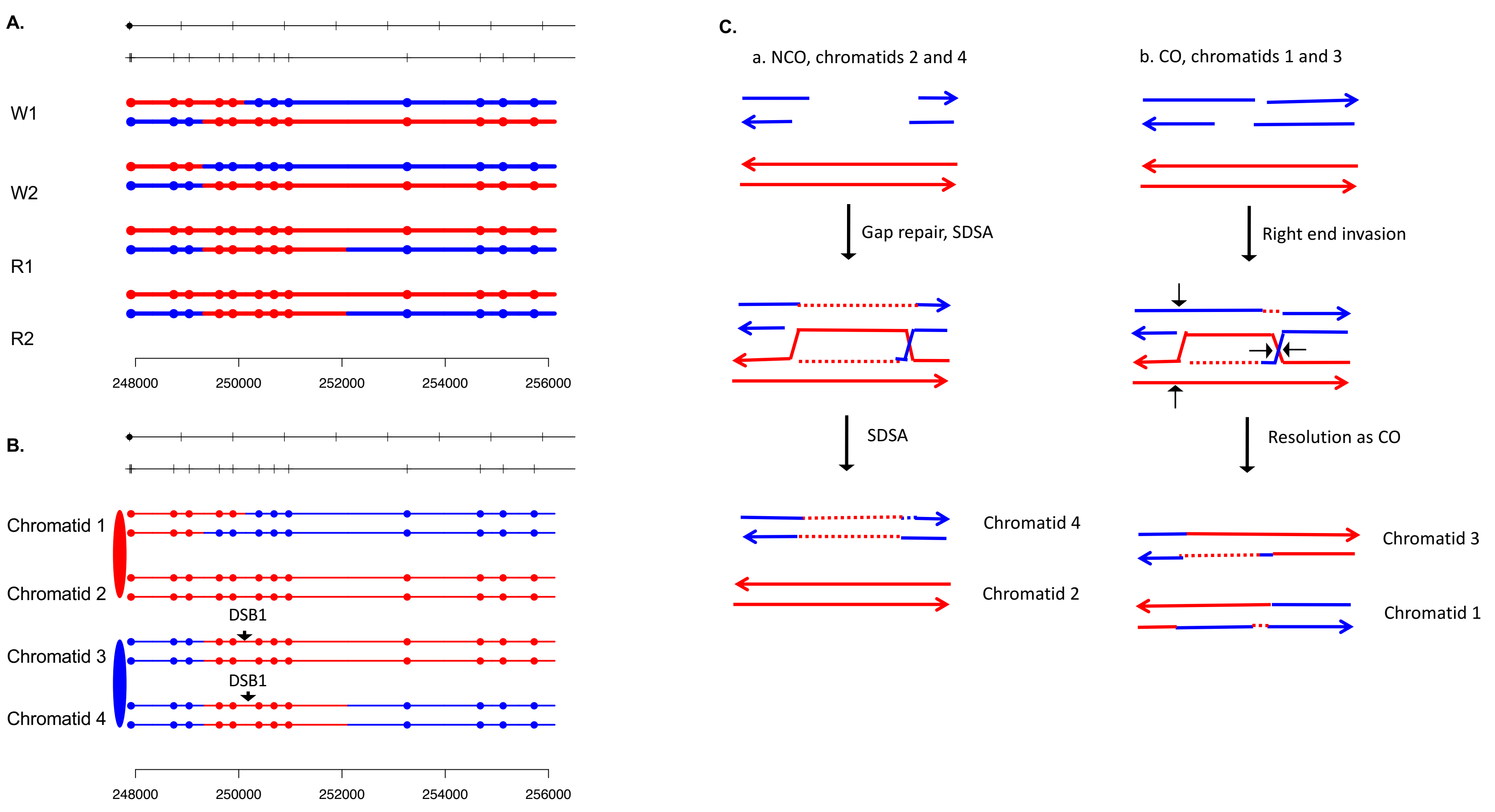

S28	NCO	Long conversion tract spanning putative DSB site	Repair of double-stranded DNA gap
	CO	Heteroduplex tract on only one side of DSB site	Standard CO according to DSBR with one heteroduplex occurring in region without SNP

S29

A.

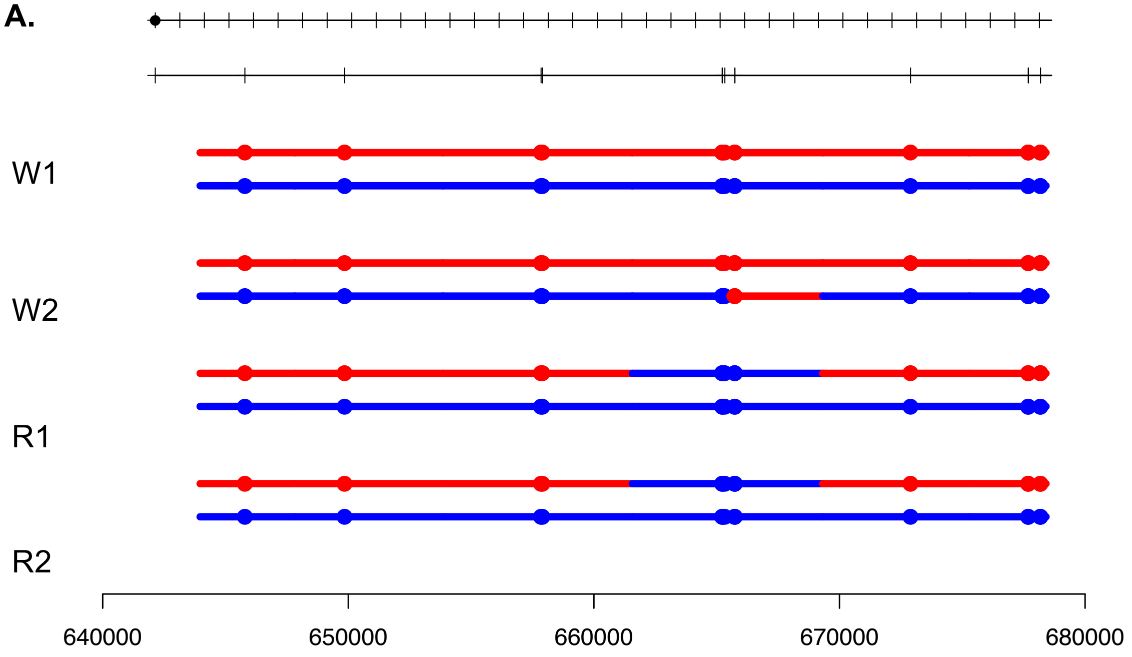
W1
W2
R1
R2
640000
650000
660000
670000
680000

B.

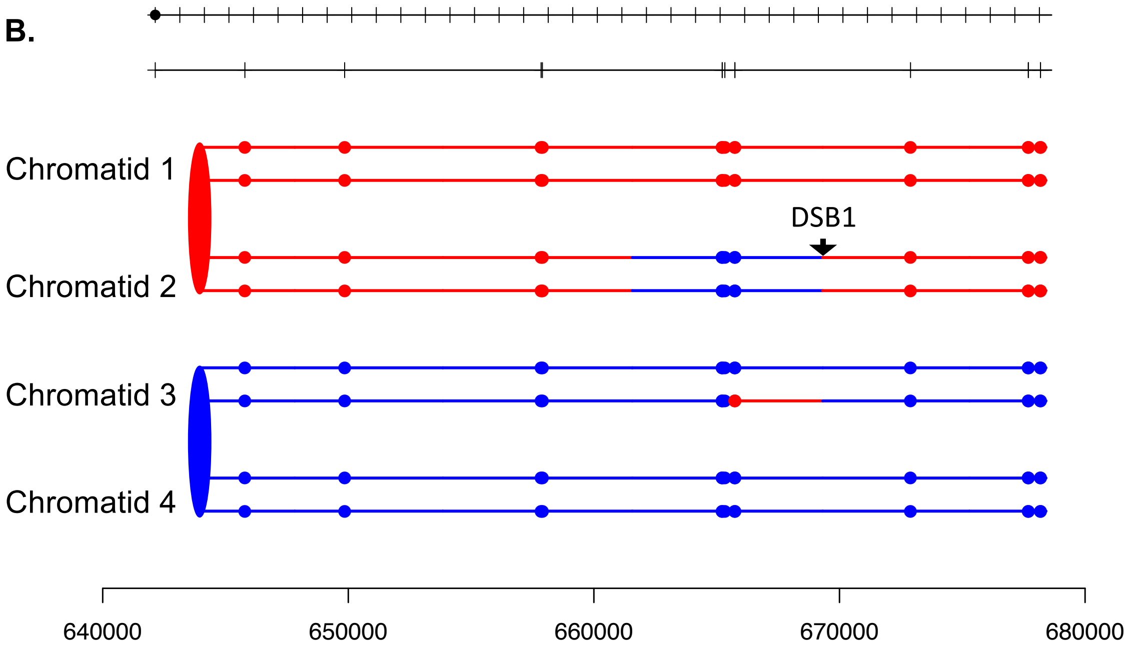
Chromatid 1
Chromatid 2
Chromatid 3
Chromatid 4
DSB1
640000
650000
660000
670000
680000

S30

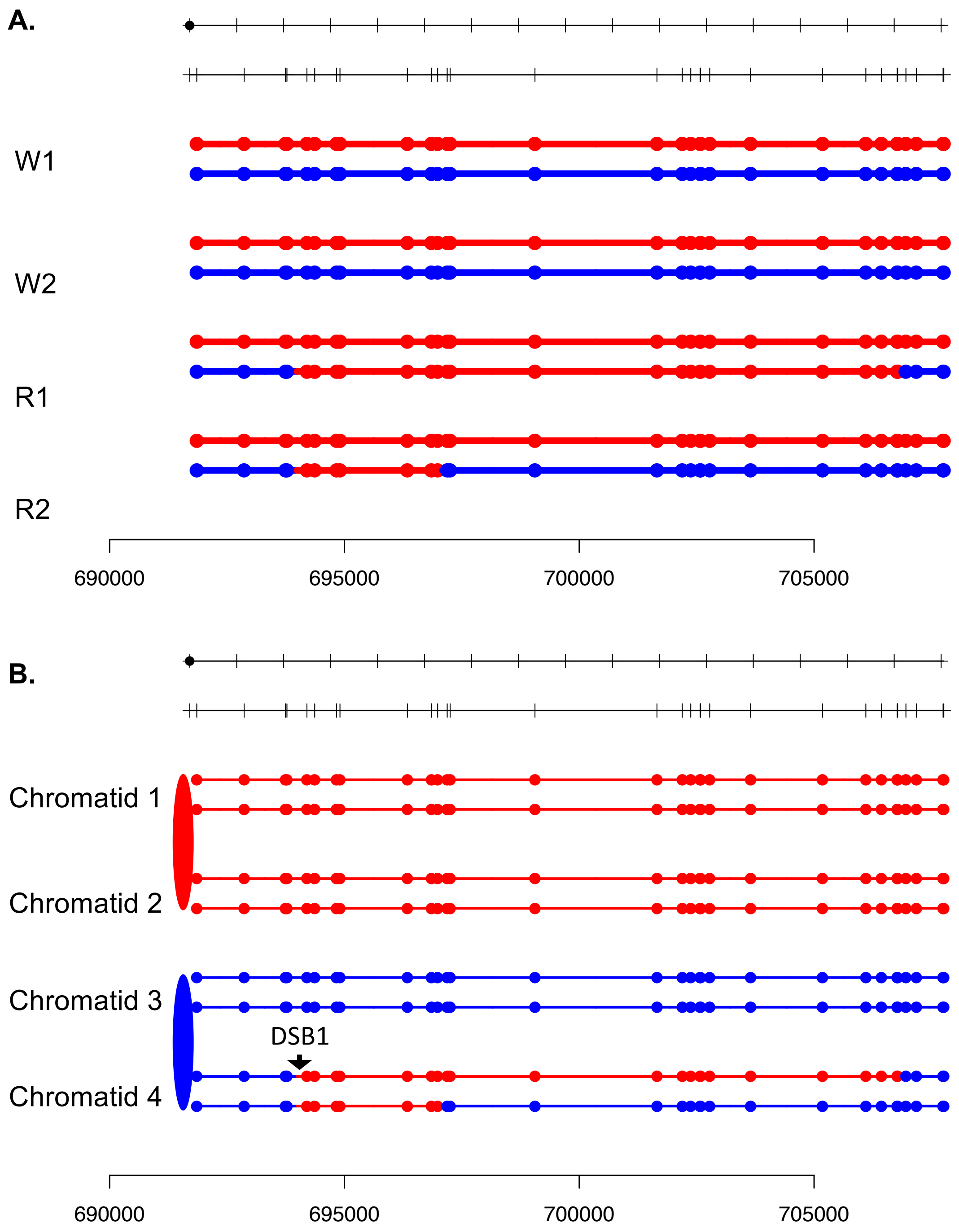
A.
W1
W2
R1
R2
690000
695000
700000
705000
B.
Chromatid 1
Chromatid 2
Chromatid 3
Chromatid 4
DSB1
690000
695000
700000
705000

S31

A.

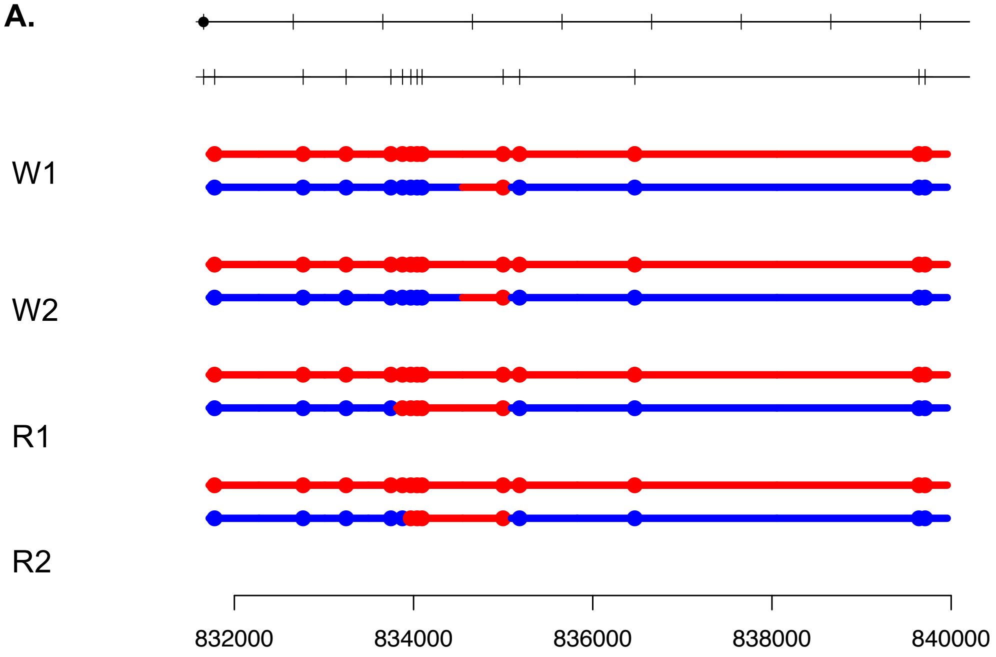
W1
W2
R1
R2
832000
834000
836000
838000
840000

B.

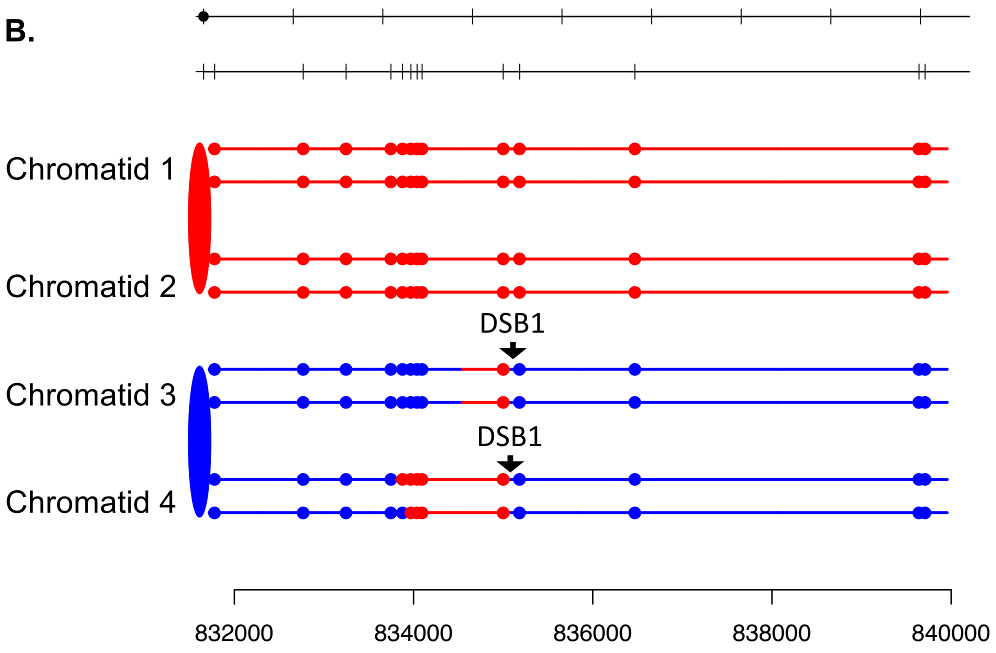
Chromatid 1
Chromatid 2
DSB1
Chromatid 3
DSB1
Chromatid 4
832000
834000
836000
838000
840000

S32

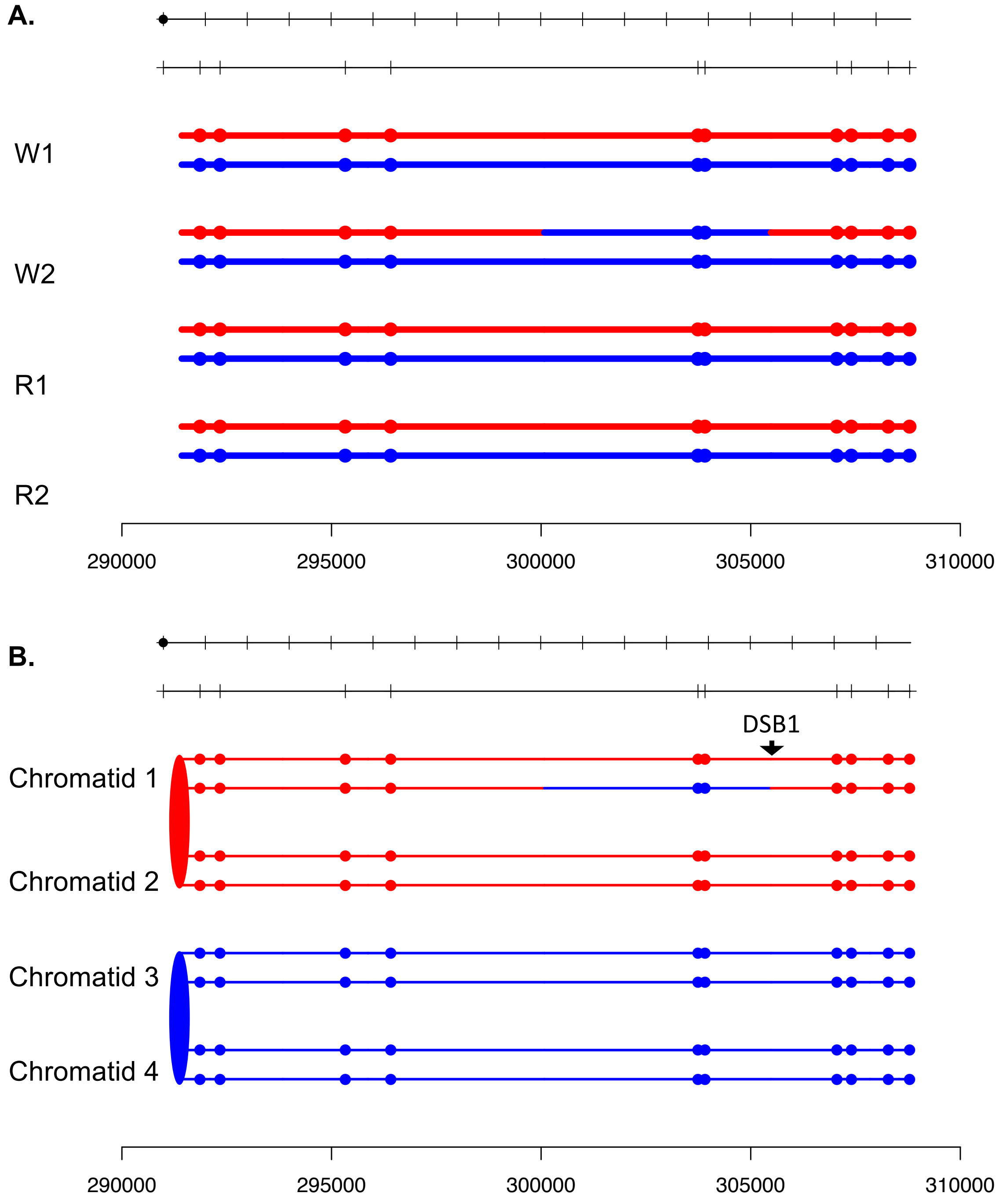
A.
W1
W2
R1
R2
290000
295000
300000
305000
310000
B.
DSB1
Chromatid 1
Chromatid 2
Chromatid 3
Chromatid 4
290000
295000
300000
305000
310000

S33

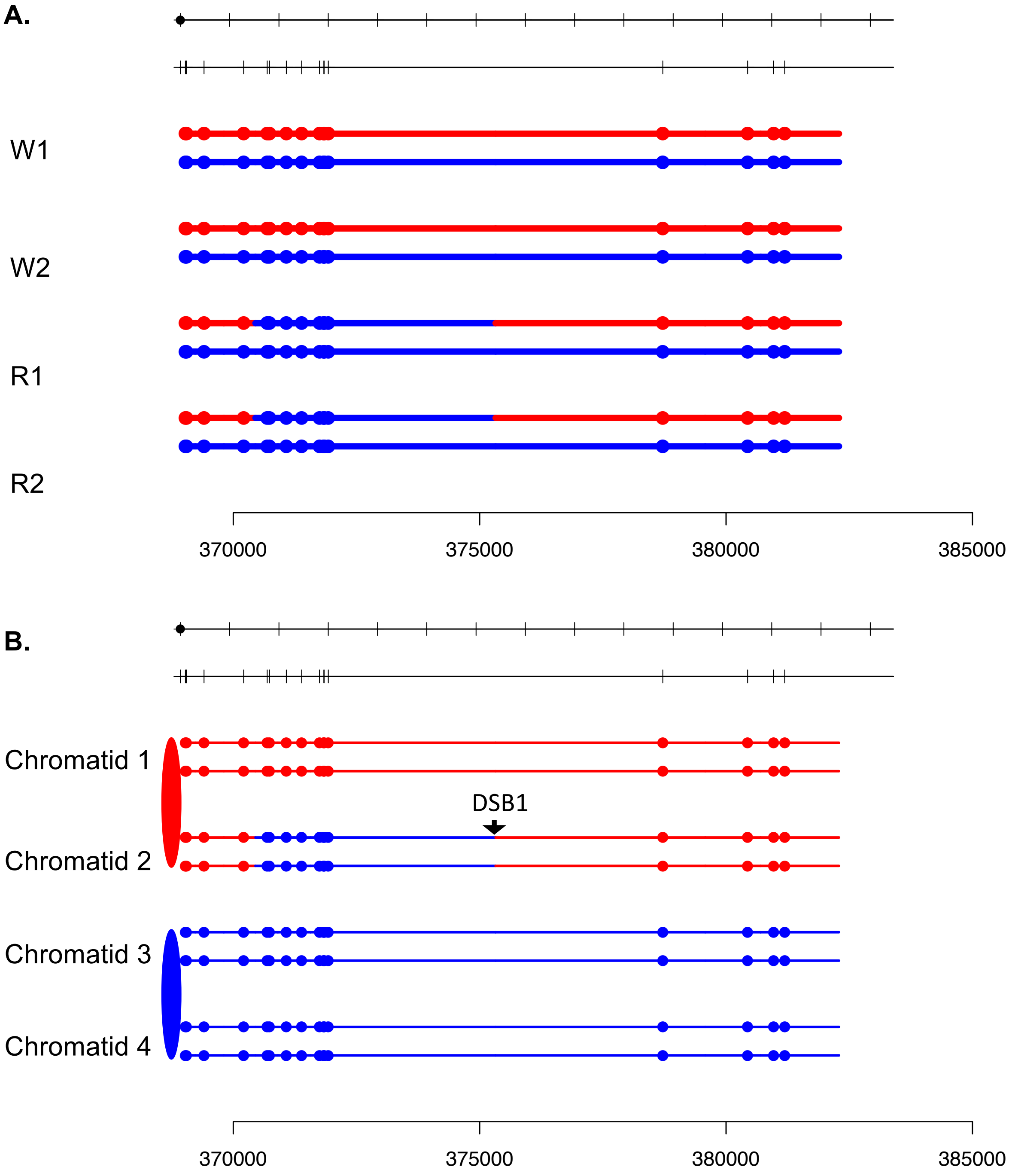
A.
W1
W2
R1
R2
370000
375000
380000
385000
B.
Chromatid 1
Chromatid 2
Chromatid 3
Chromatid 4
DSB1
370000
375000
380000
385000

S34

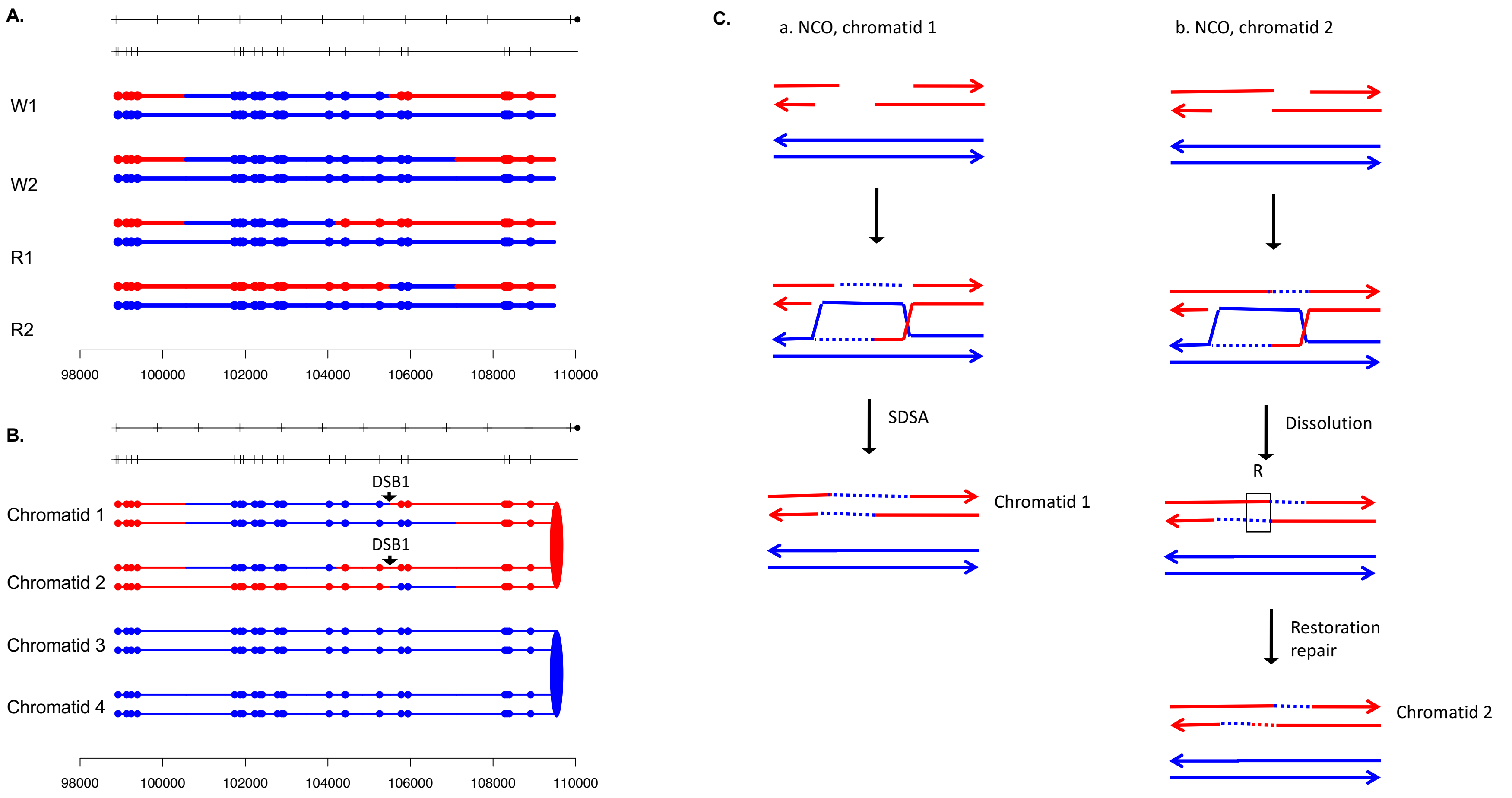

S34	NCO1	Long conversion-tract on chromatid 1	Repair of double-stranded DNA gap
	NCO2	Heteroduplexes in trans on chromatid 2 separated by homoduplex	Dissolution of dHJ associated with restoration repair

S35

A.

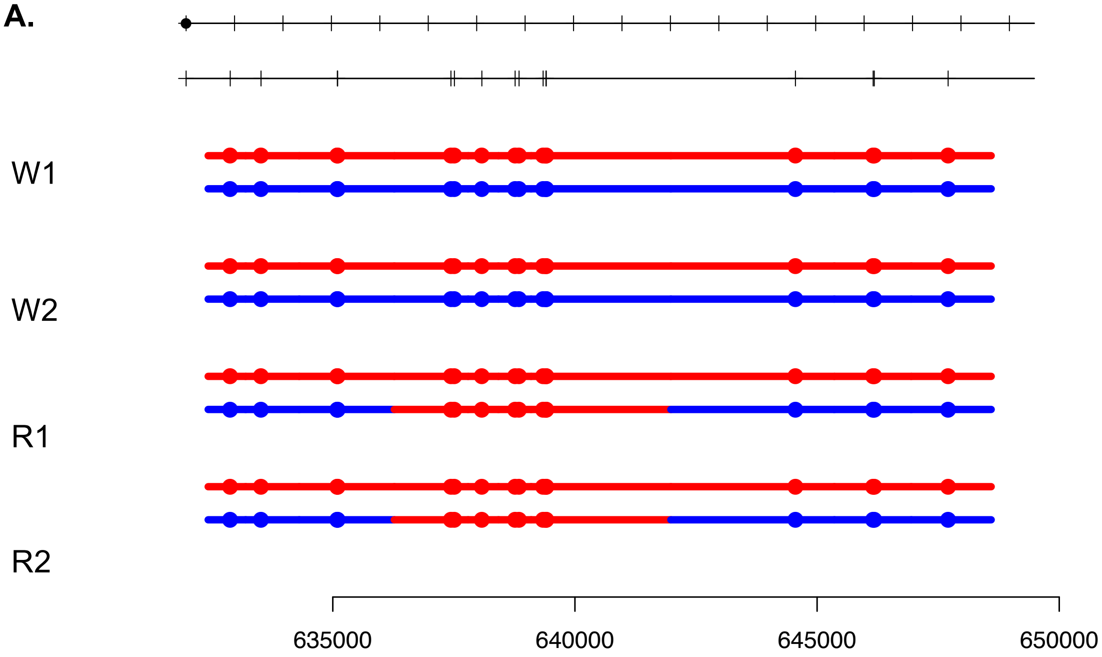
W1
W2
R1
R2
635000
640000
645000
650000

B.

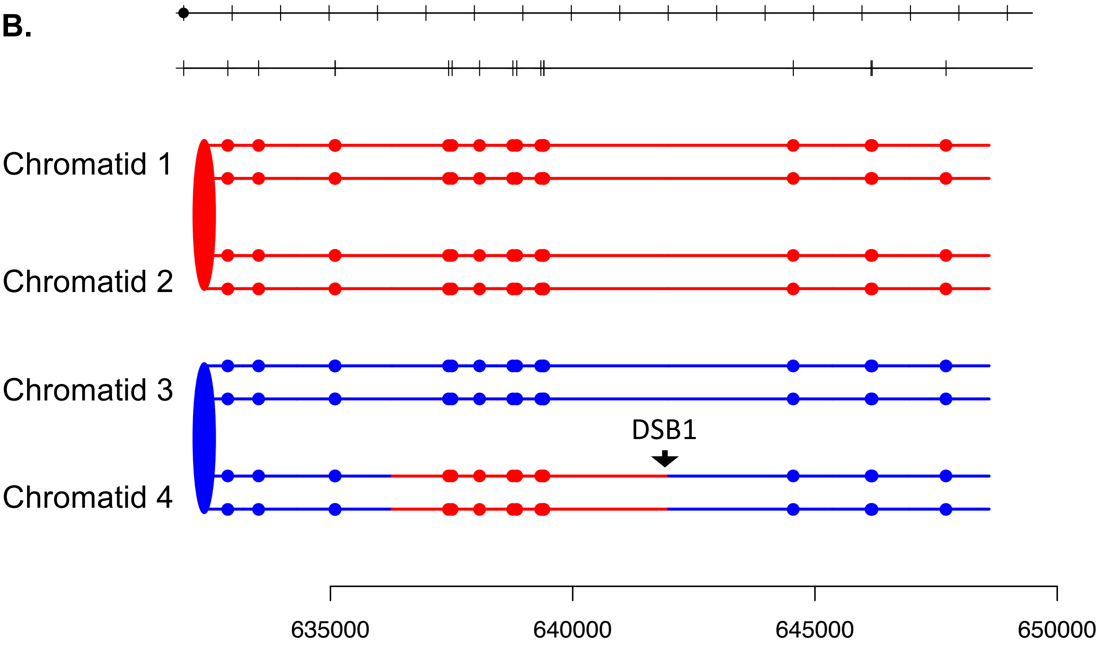
Chromatid 1
Chromatid 2
Chromatid 3
Chromatid 4
DSB1
635000
640000
645000
650000

S36

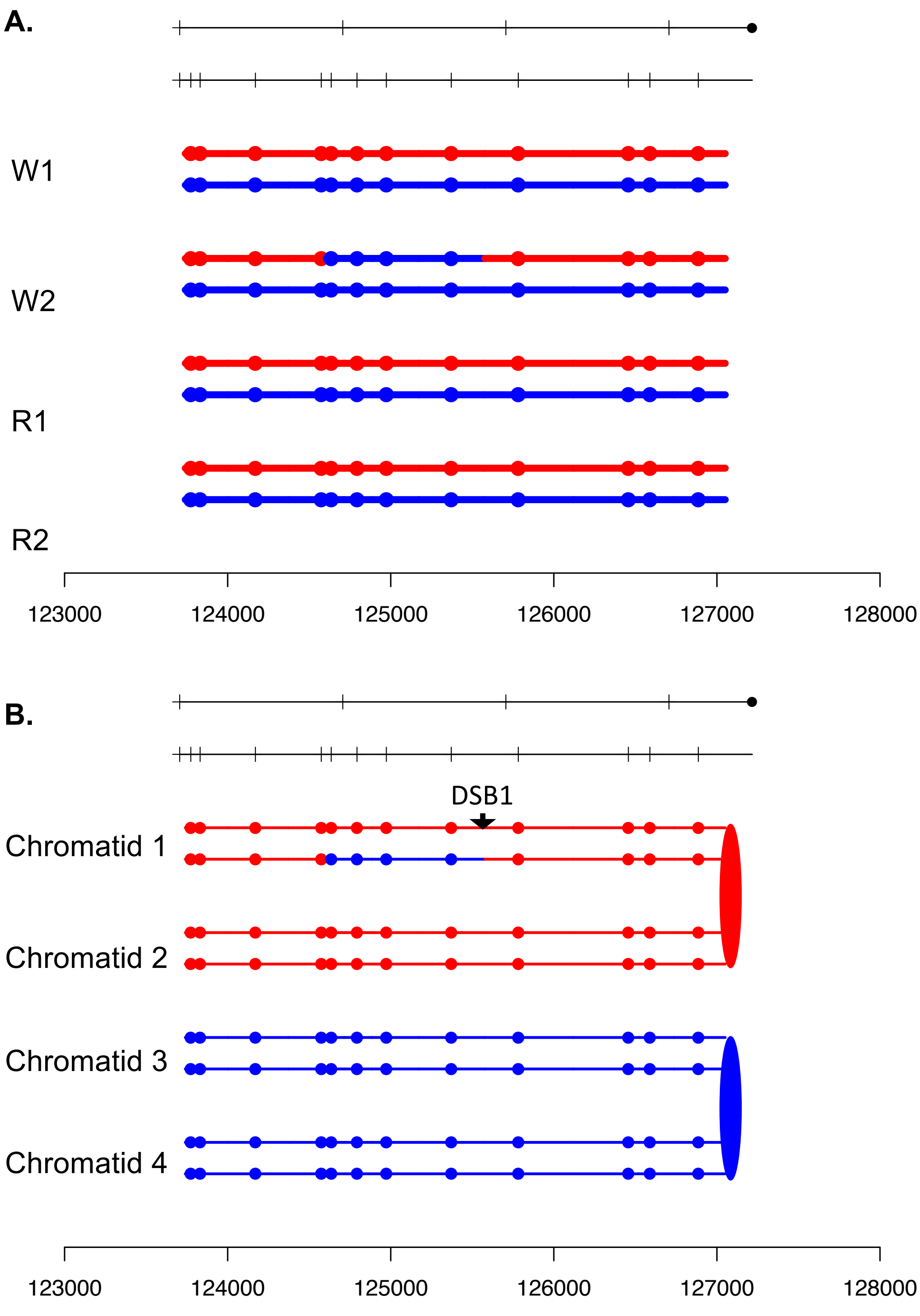
A.
W1
W2
R1
R2
123000
124000
125000
126000
127000
128000
B.
DSB1
Chromatid 1
Chromatid 2
Chromatid 3
Chromatid 4
123000
124000
125000
126000
127000
128000

S37

A.

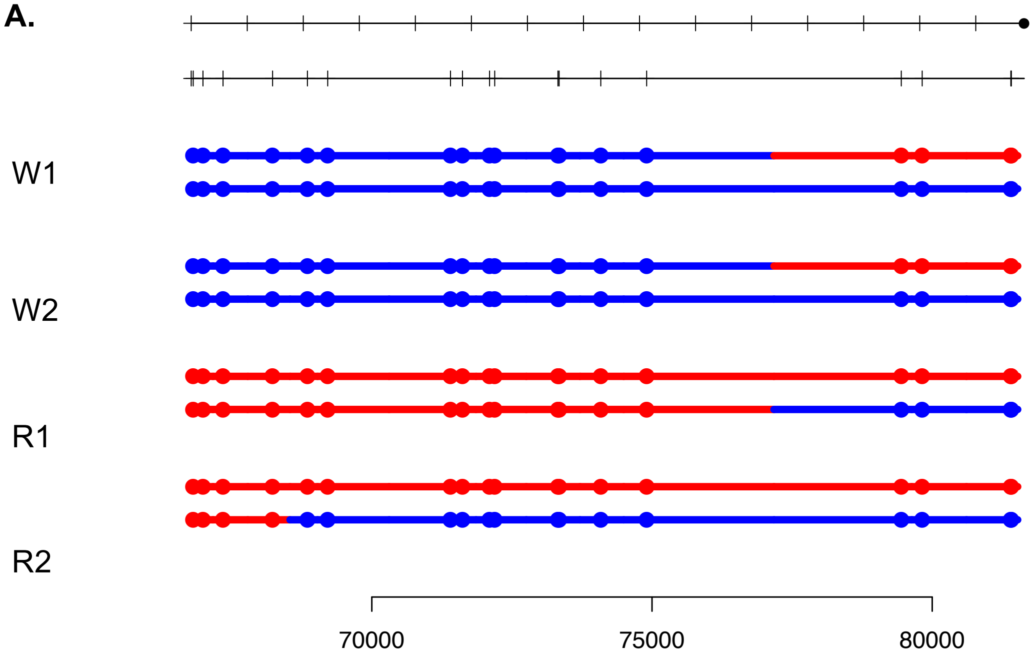
W1
W2
R1
R2
70000
75000
80000

B.

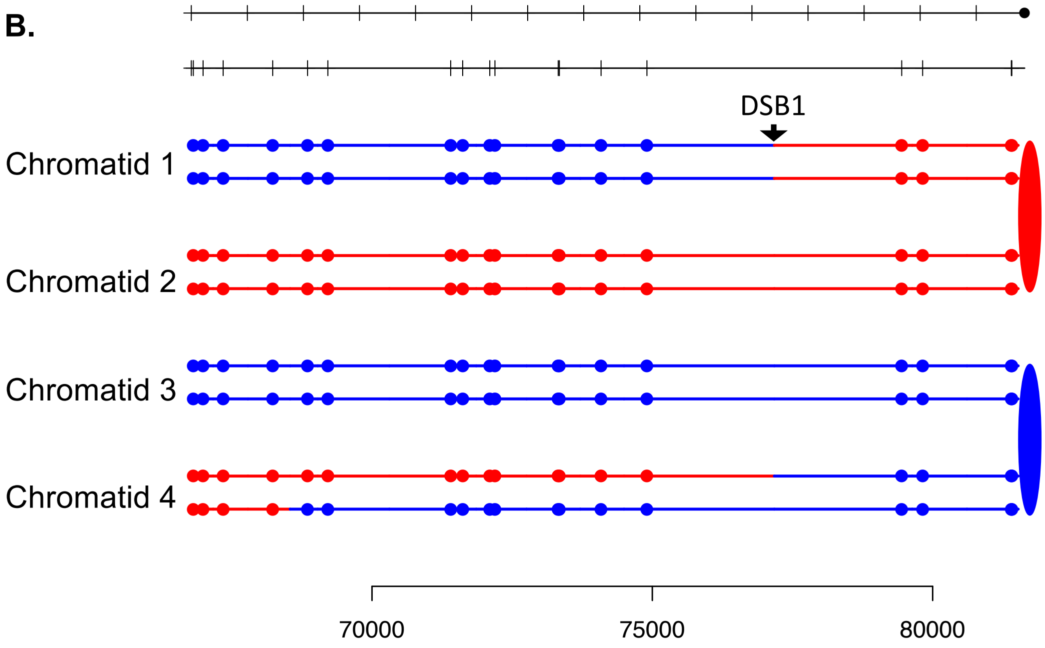
DSB1
Chromatid 1
Chromatid 2
Chromatid 3
Chromatid 4
70000
75000
80000

S38

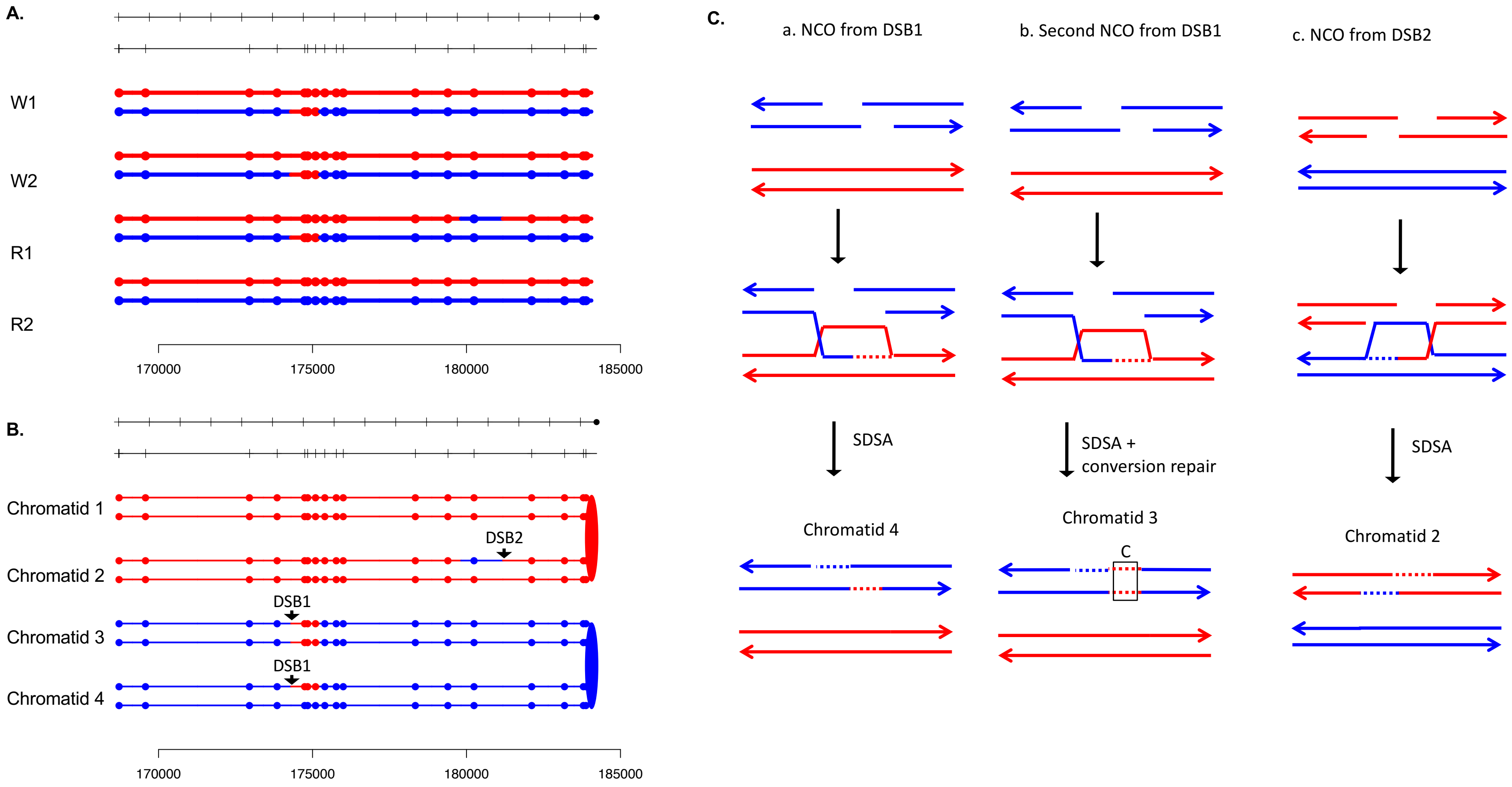

S38	NCO1	Conversion tract adjacent to DSB1 site on chromatid 3	SDSA, Mlh1-independent MMR
	NCO2	Heteroduplex adjacent to DSB1 site on chromatid 4	SDSA
	NCO3	Heteroduplex adjacent to DSB2 site on chromatid 2	SDSA

S39

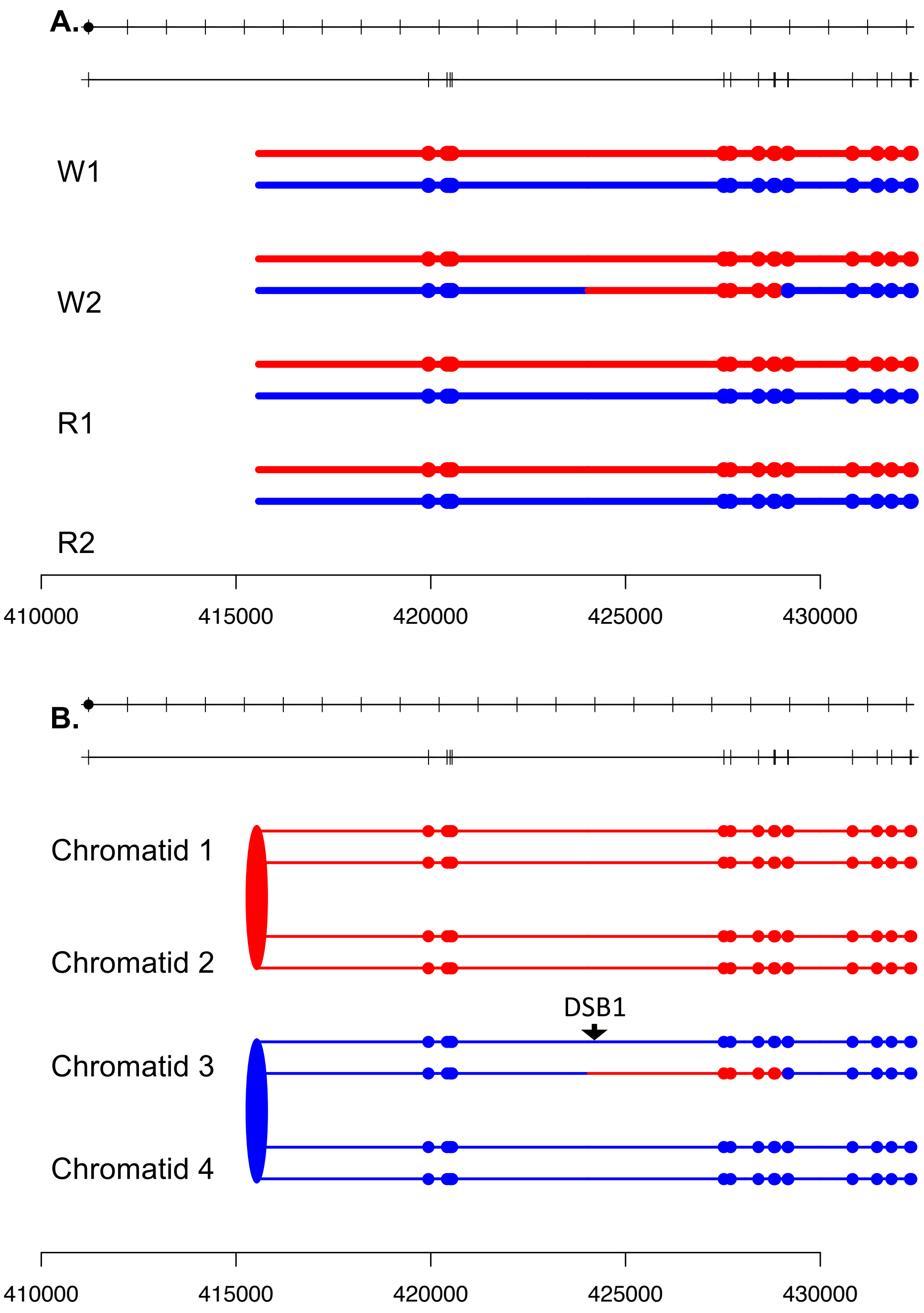
A.
W1
W2
R1
R2
410000
415000
420000
425000
430000
B.
Chromatid 1
Chromatid 2
DSB1
Chromatid 3
Chromatid 4
410000
415000
420000
425000
430000

S40

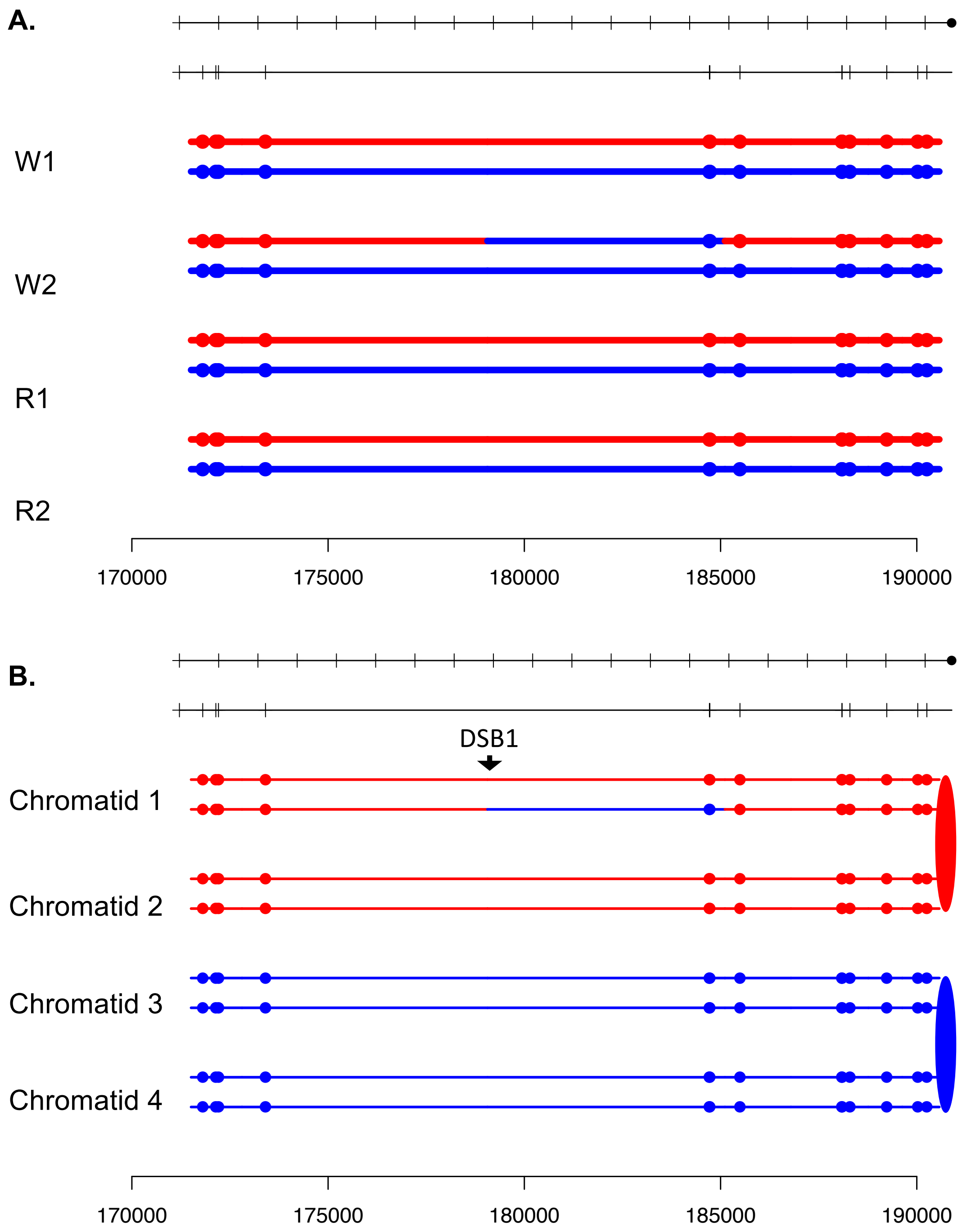
A.
W1
W2
R1
R2
170000
175000
180000
185000
190000
B.
DSB1
Chromatid 1
Chromatid 2
Chromatid 3
Chromatid 4
170000
175000
180000
185000
190000

S41

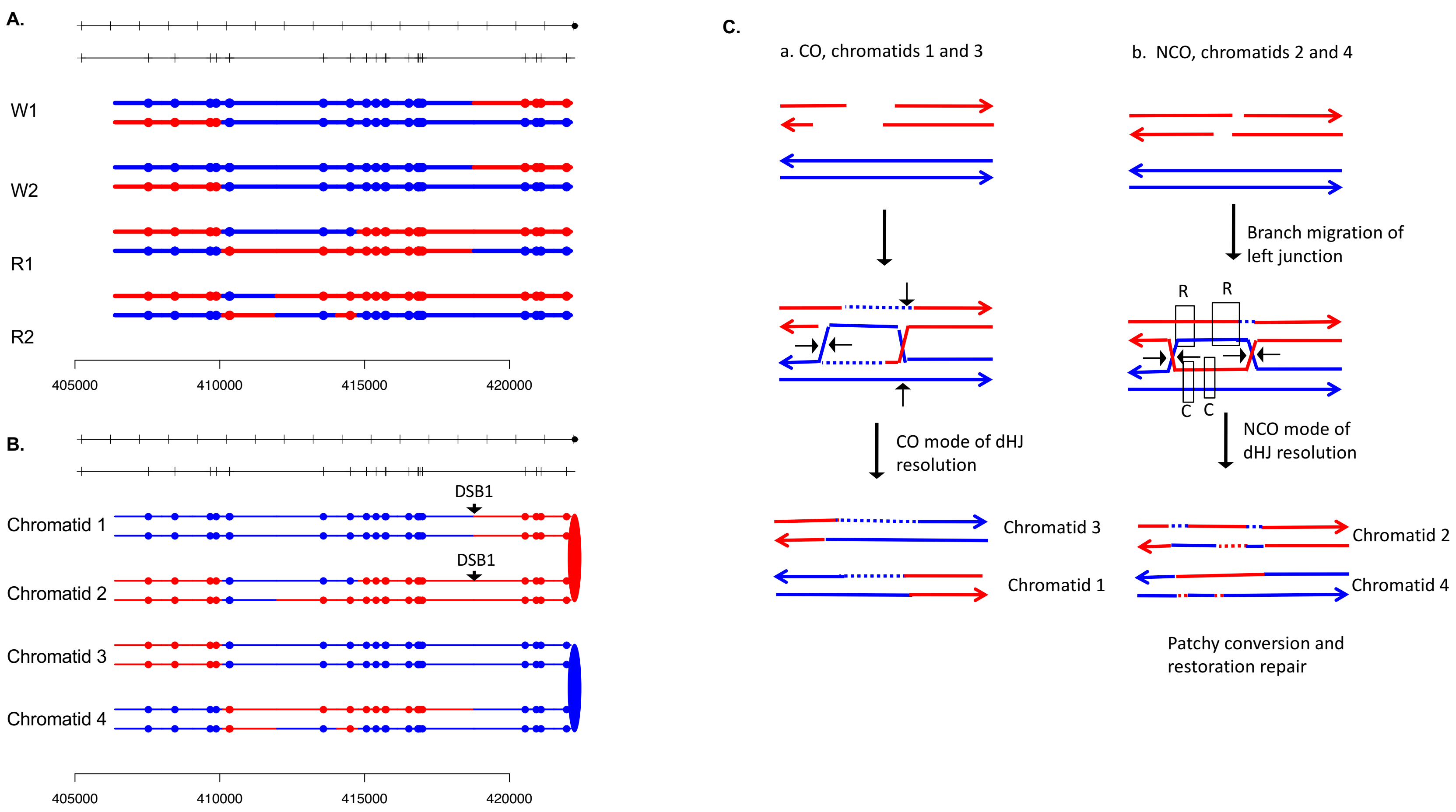

S41	NCO	Symmetric heteroduplexes on chromatids 2 and 4; homoduplex regions within heteroduplex	Branch migration; Mlh1-independent MMR
	CO	Crossover between chromatids 1 and 3 associated with long conversion tract	Repair of double-stranded DNA gap

S42

A.

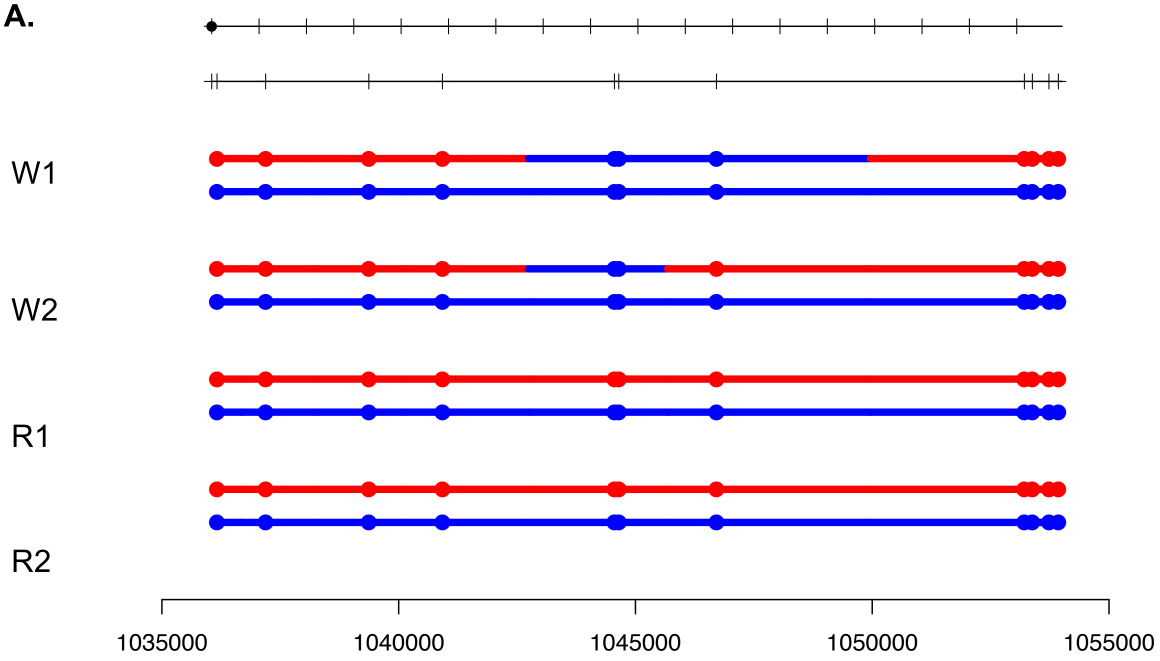
W1
W2
R1
R2
1035000
1040000
1045000
1050000
1055000

B.

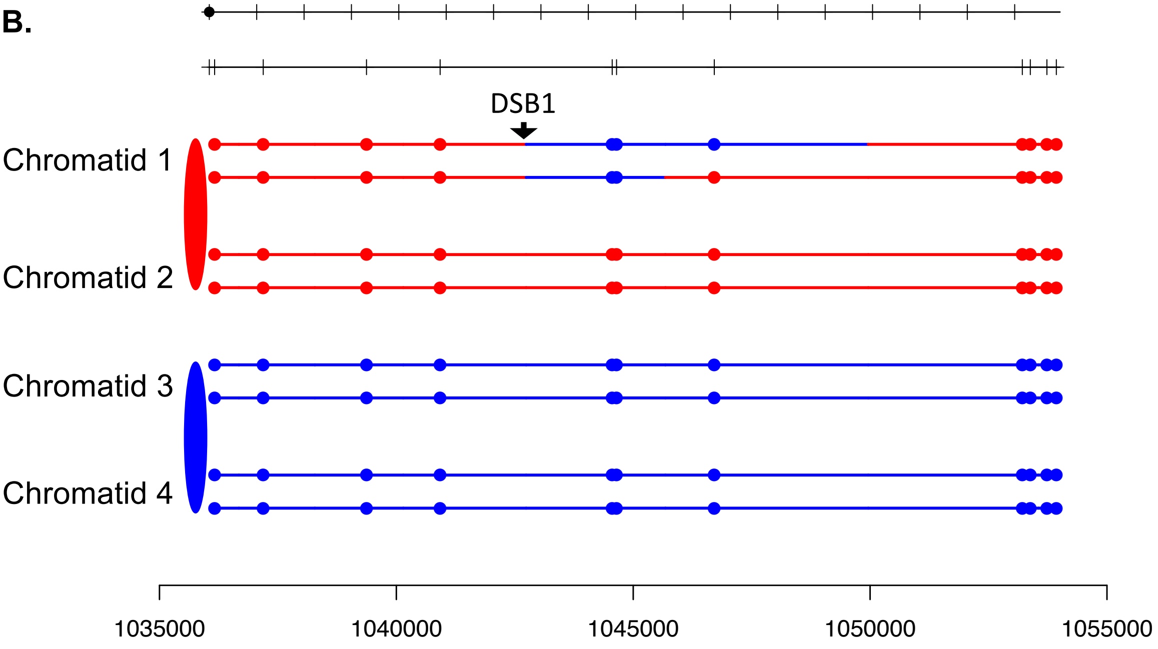
DSB1
Chromatid 1
Chromatid 2
Chromatid 3
Chromatid 4
1035000
1040000
1045000
1050000
1055000

S43

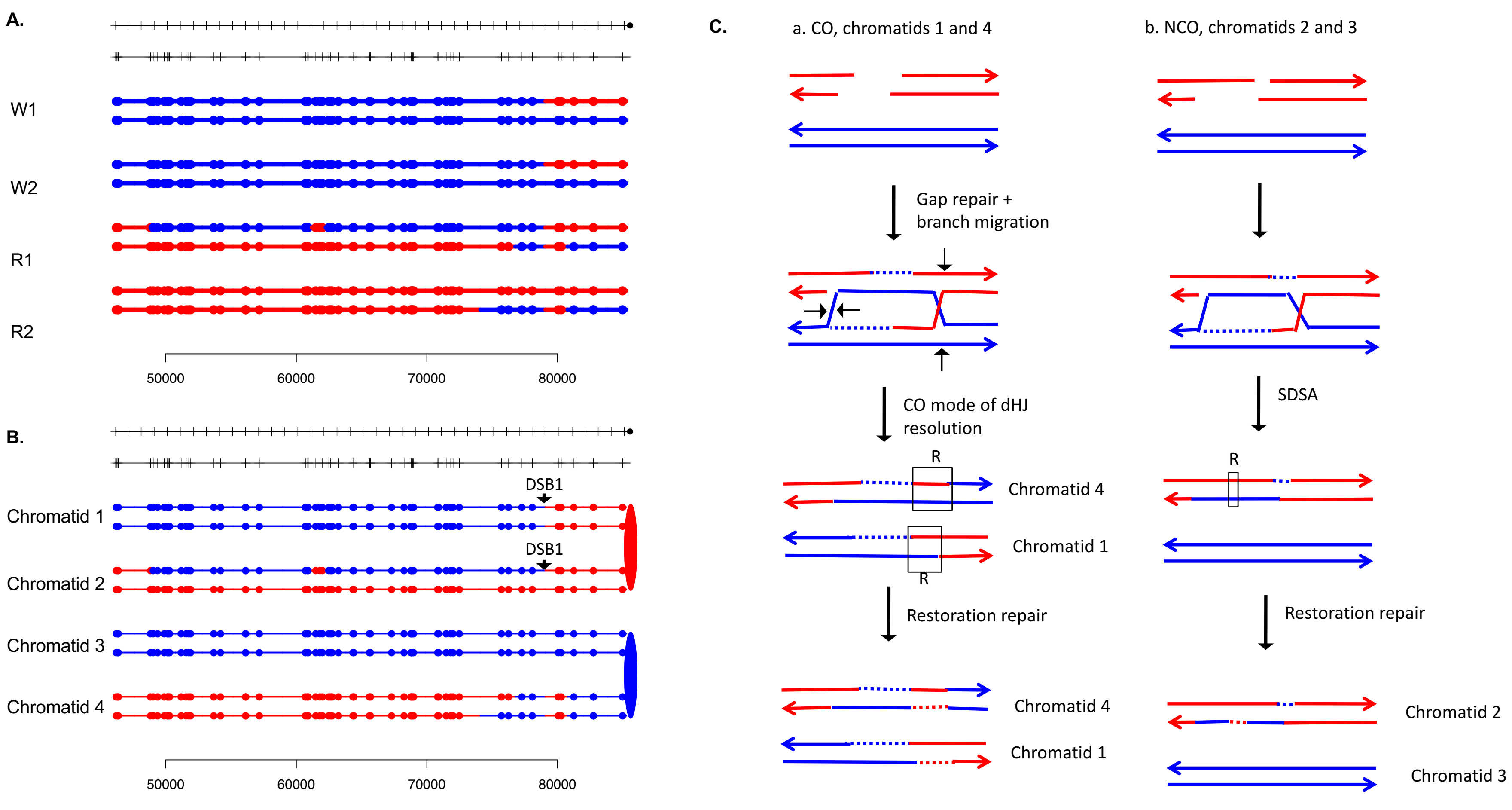

S43	CO	Restoration repair on one side of CO chromatid 4 and conversion repair on the other	Branch migration; Mlh1-independent MMR
	NCO	Long heteroduplex region with short restoration tract	SDSA with Mlh1-independent MMR

S44

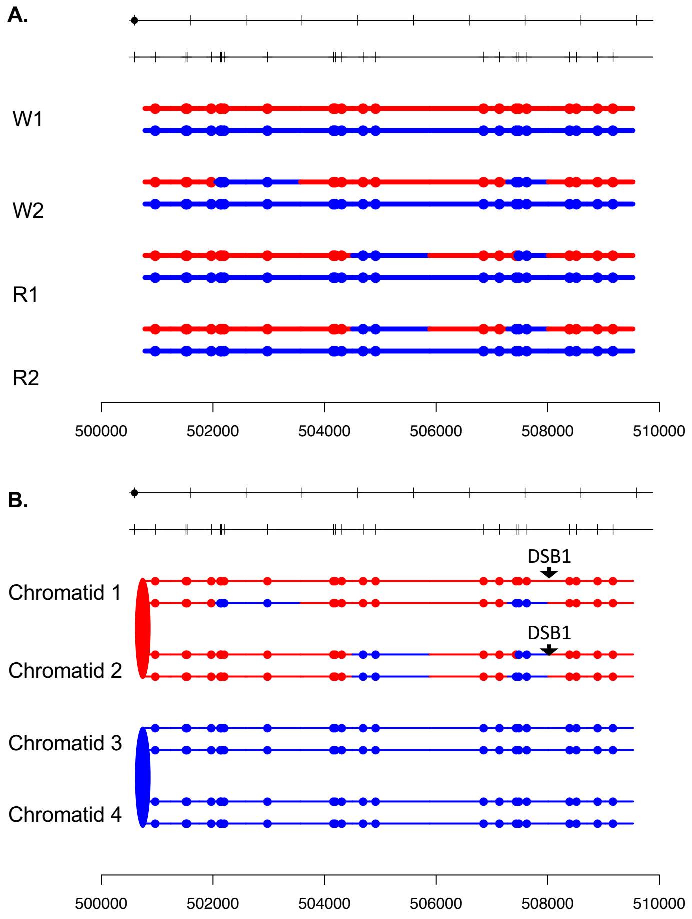

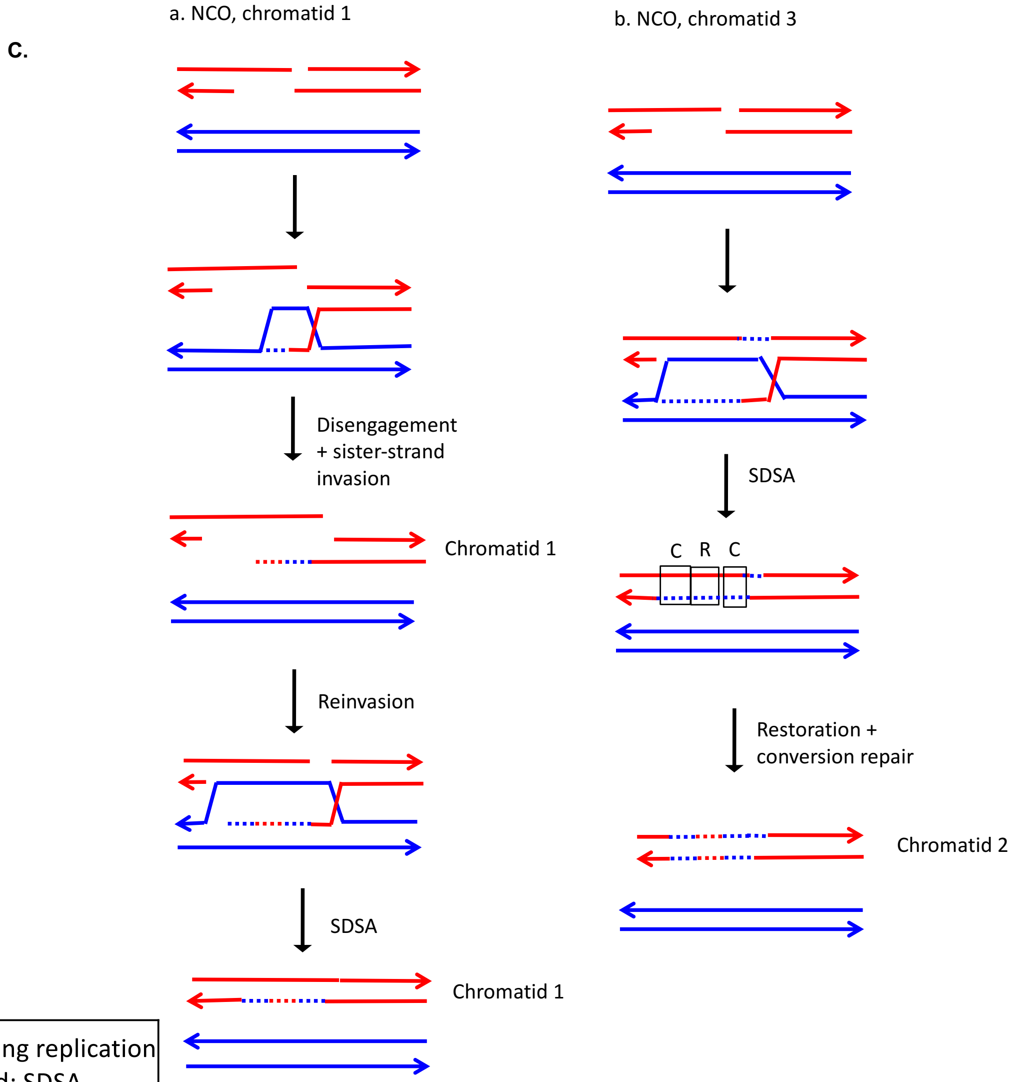

S44	NCO1	Chromatid 1 has heteroduplex region with long restoration tract in the middle	Template switch during replication to sister strand; SDSA
	NCO2	Chromatid 2 has mixture of homoduplex conversion and restoration tracts	Mlh1-independent patchy MMR

S45

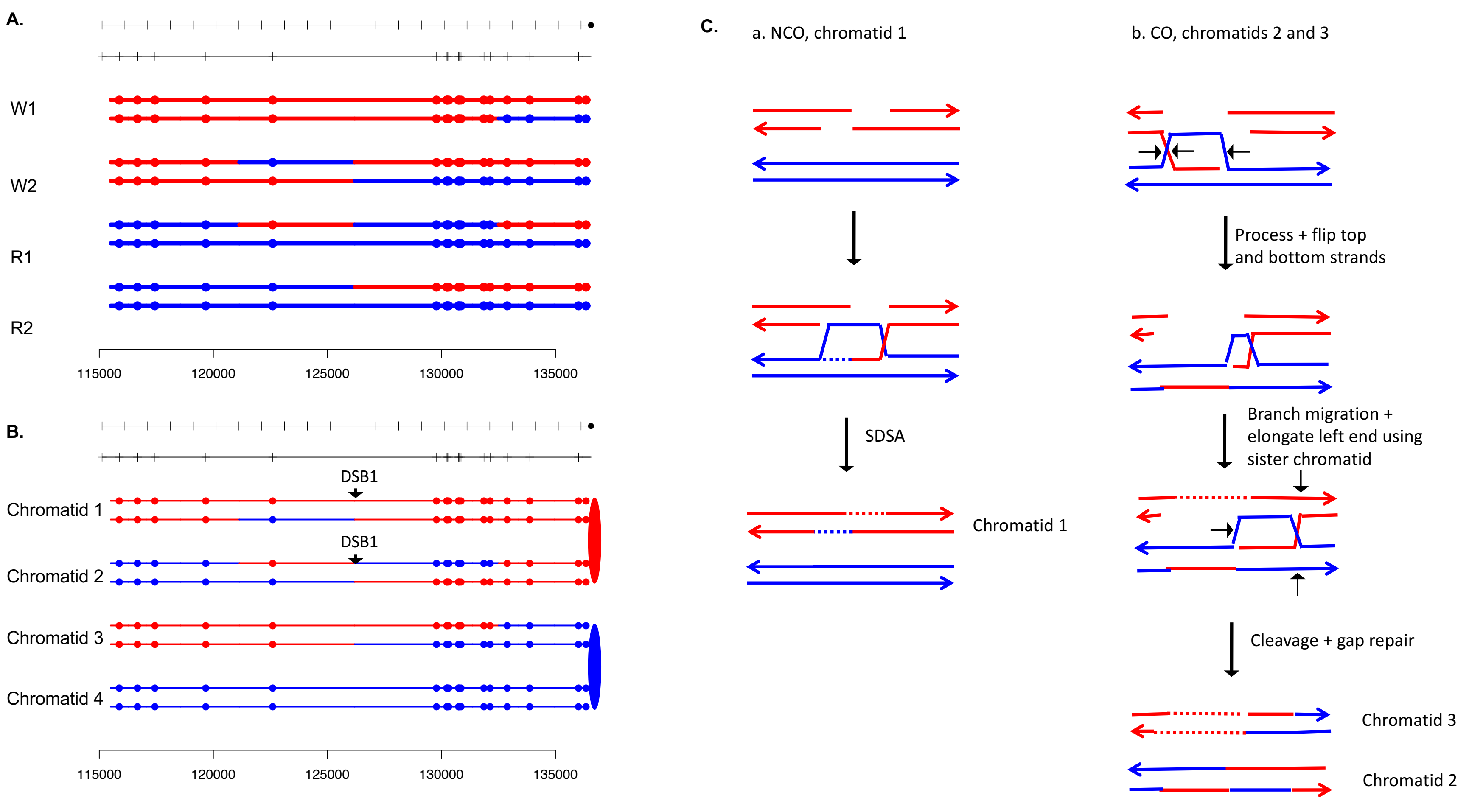

S45	NCO	Simple heteroduplex (chromatid 1)	SDSA
	CO	Regions of heteroduplex with switched strands on chromatid 3; symmetric heteroduplexes	Independent invasion of two broken ends; branch migration

S46

A.

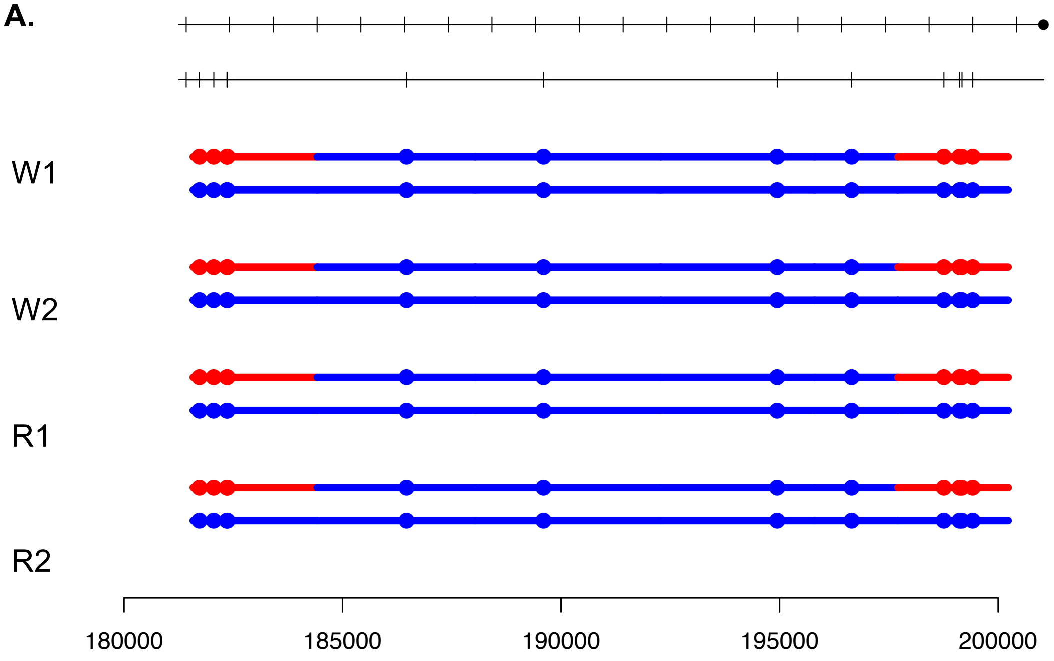
W1
W2
R1
R2
180000
185000
190000
195000
200000

B.

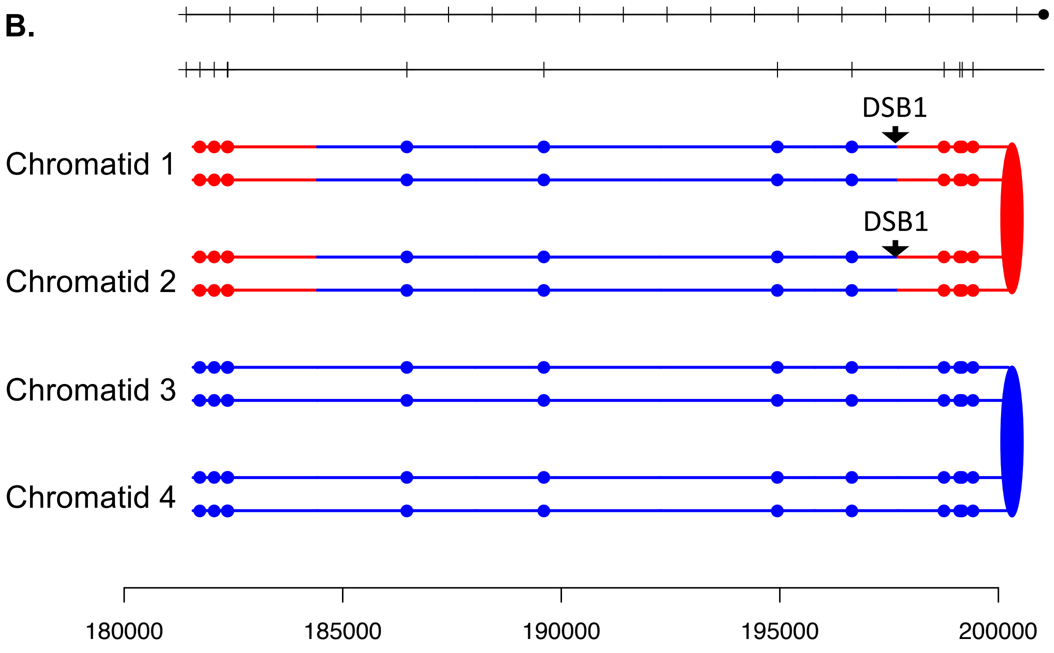
DSB1
DSB1
Chromatid 1
Chromatid 2
Chromatid 3
Chromatid 4
180000
185000
190000
195000
200000

S47

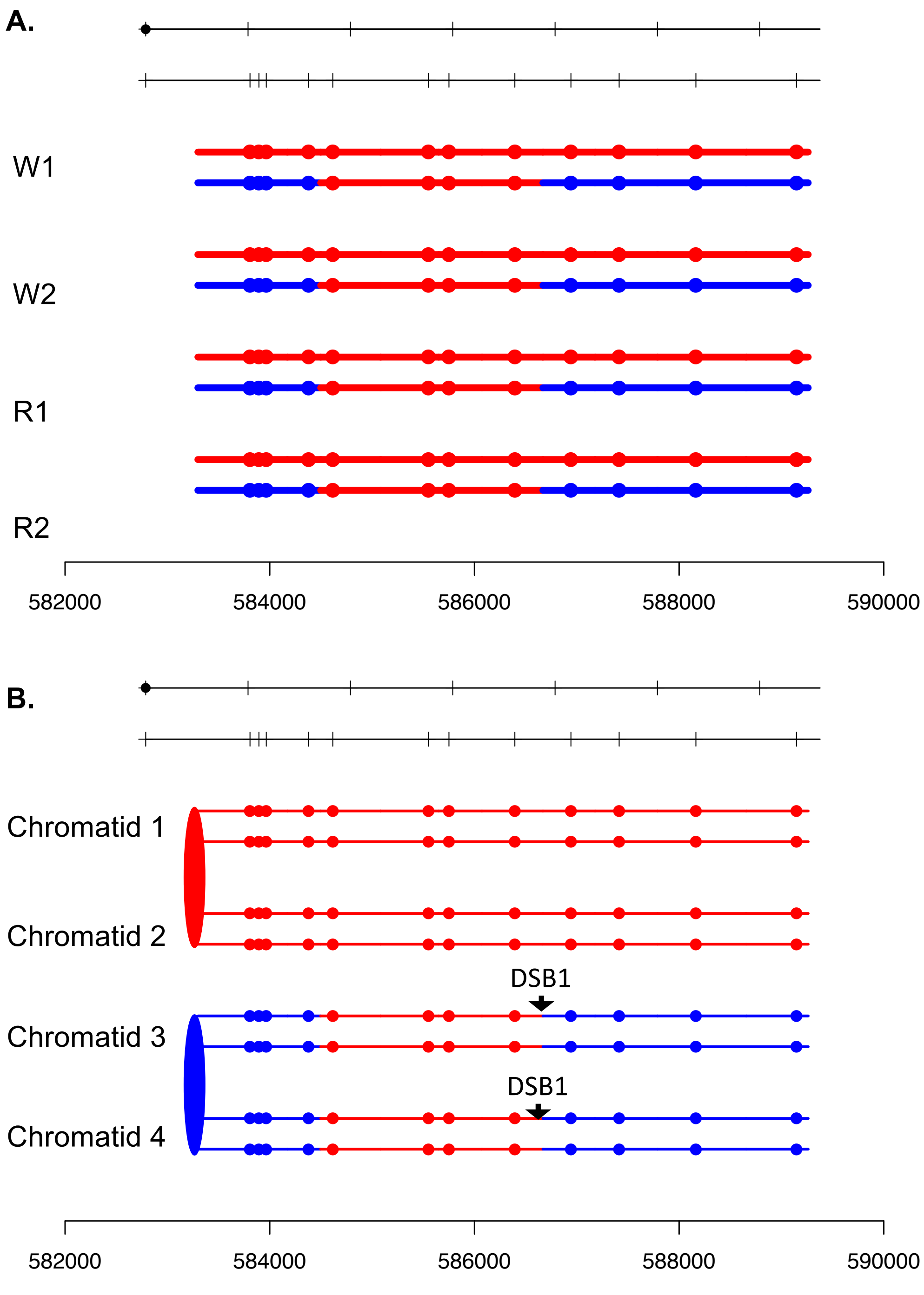

A.
W1
W2
R1
R2
582000
584000
586000
588000
590000
B.
Chromatid 1
Chromatid 2
Chromatid 3
Chromatid 4
DSB1
DSB1
582000
584000
586000
588000
590000

S48

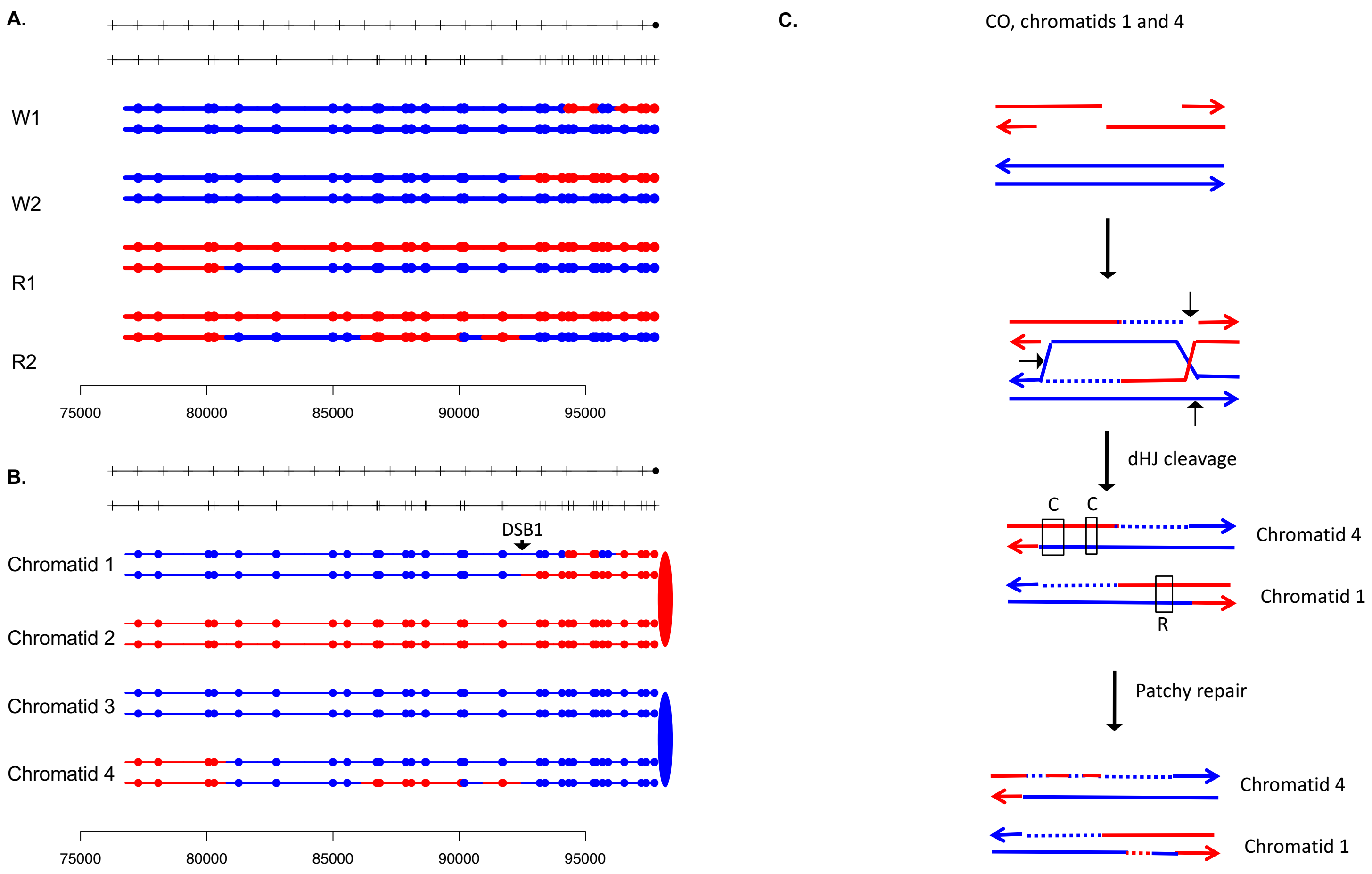

S48	CO	Regions of heteroduplex interspersed with homoduplex regions	Mlh1-independent patchy MMR

S49

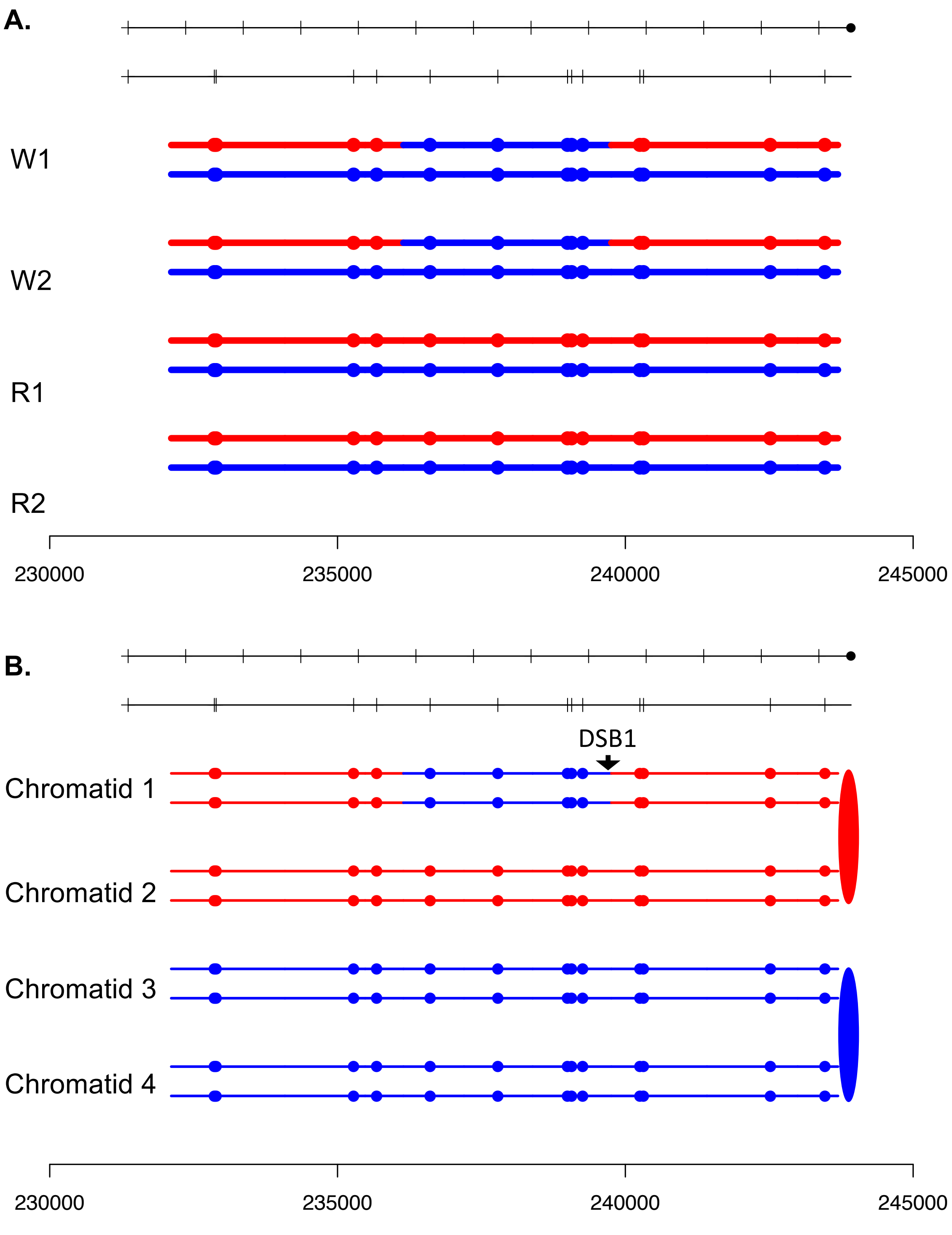
A.
W1
W2
R1
R2
230000
235000
240000
245000
B.
DSB1
Chromatid 1
Chromatid 2
Chromatid 3
Chromatid 4
230000
235000
240000
245000

S50

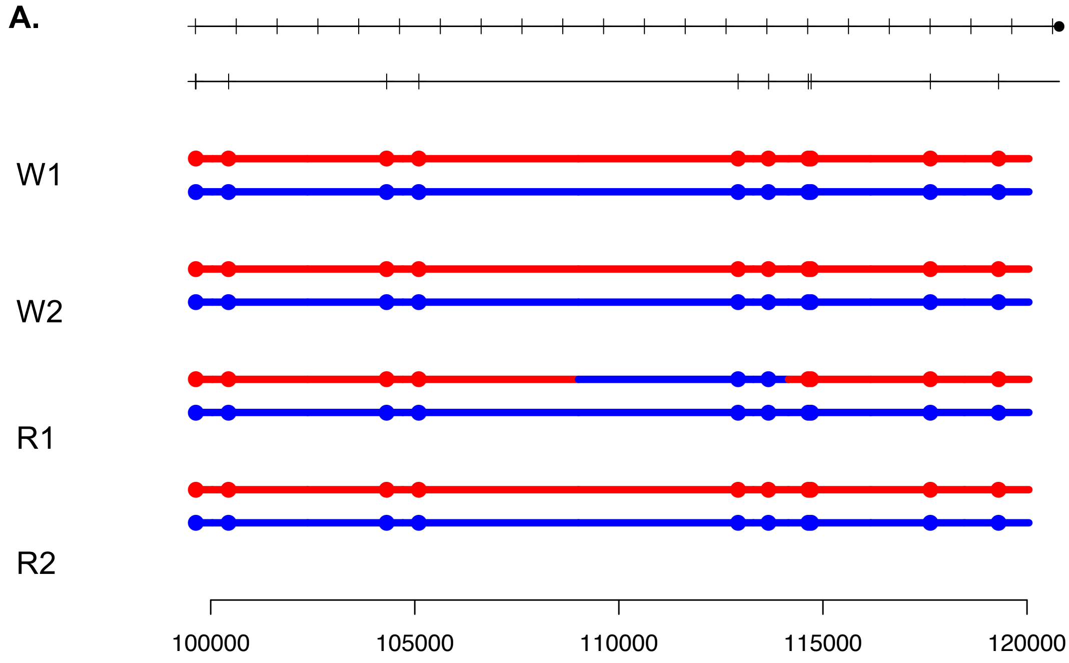
A.
W1
W2
R1
R2
100000
105000
110000
115000
120000

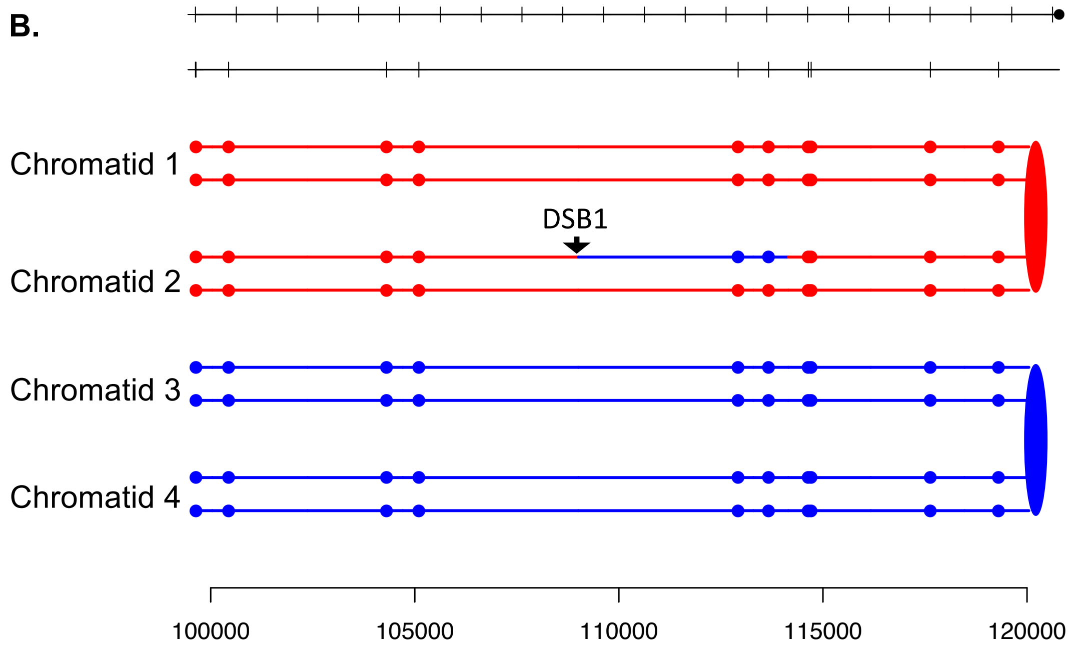
B.
Chromatid 1
DSB1
Chromatid 2
Chromatid 3
Chromatid 4
100000
105000
110000
115000
120000

S51

A.

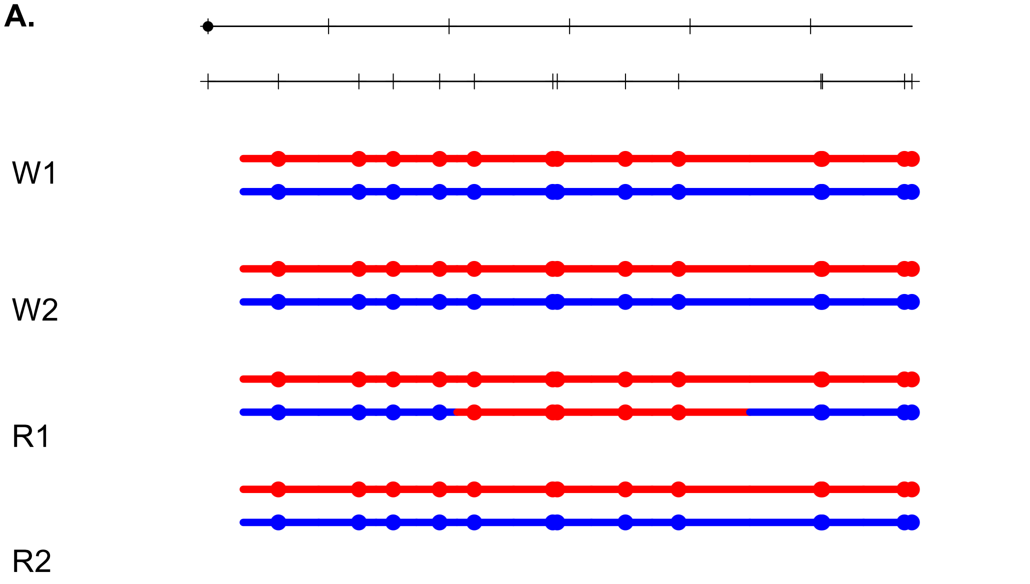
W1
W2
R1
R2
756000
758000
760000
762000

B.

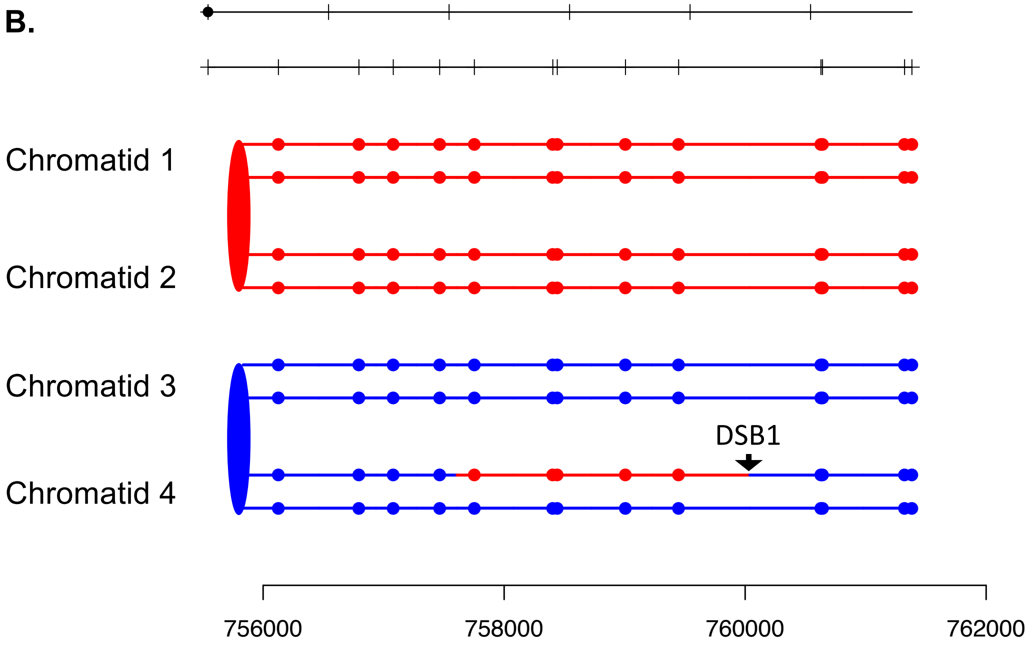
Chromatid 1
Chromatid 2
Chromatid 3
Chromatid 4
DSB1
756000
758000
760000
762000

S52

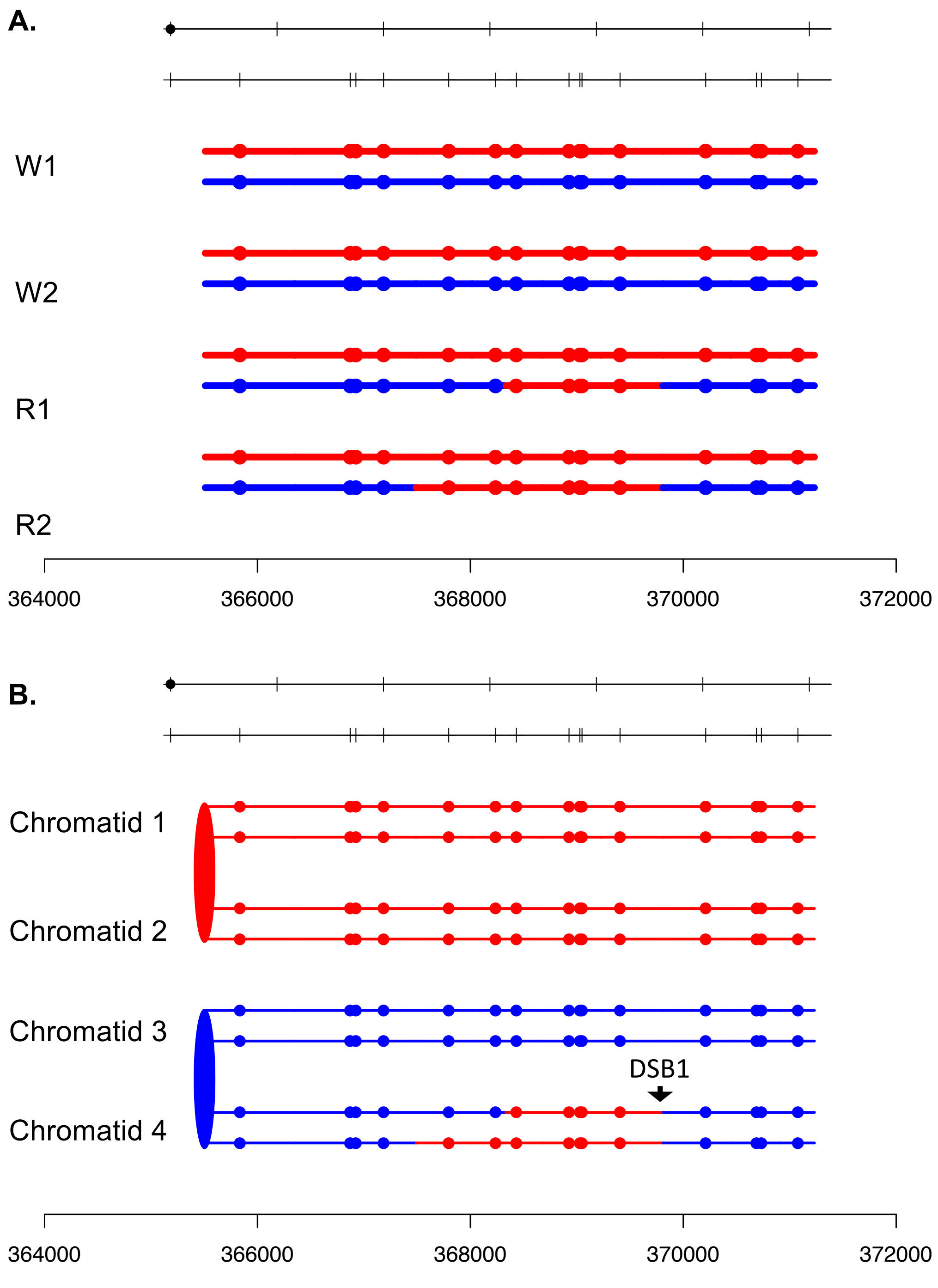
A.
W1
W2
R1
R2
364000
366000
368000
370000
372000
B.
Chromatid 1
Chromatid 2
Chromatid 3
Chromatid 4
DSB1
364000
366000
368000
370000
372000

S53

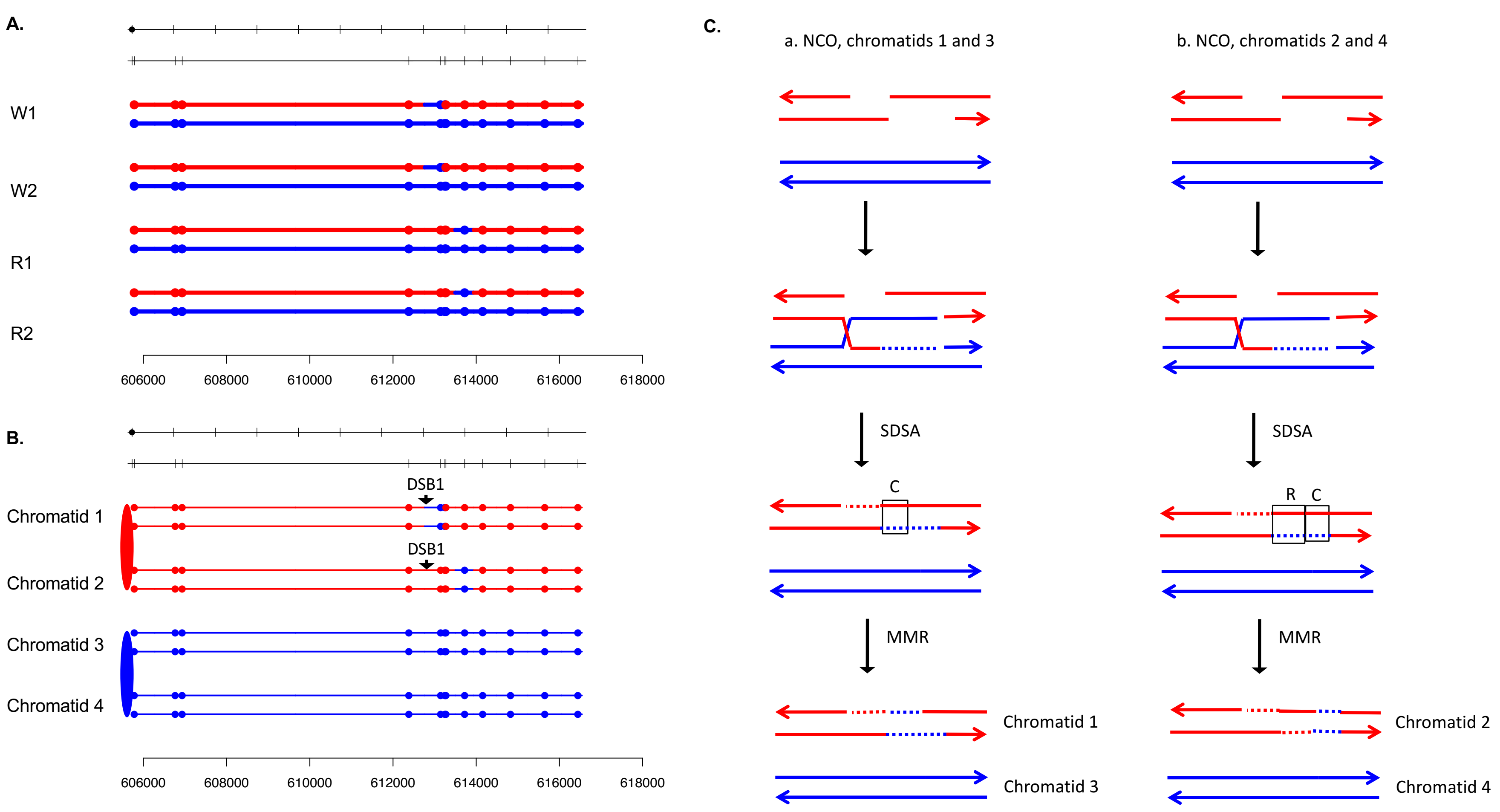

S53	NCO1	Conversion tract with no heteroduplex in chromatid 1	Mlh1-independent MMR
	NCO2	Conversion and restoration tracts in chromatid 2	Mlh1-independent patchy MMR

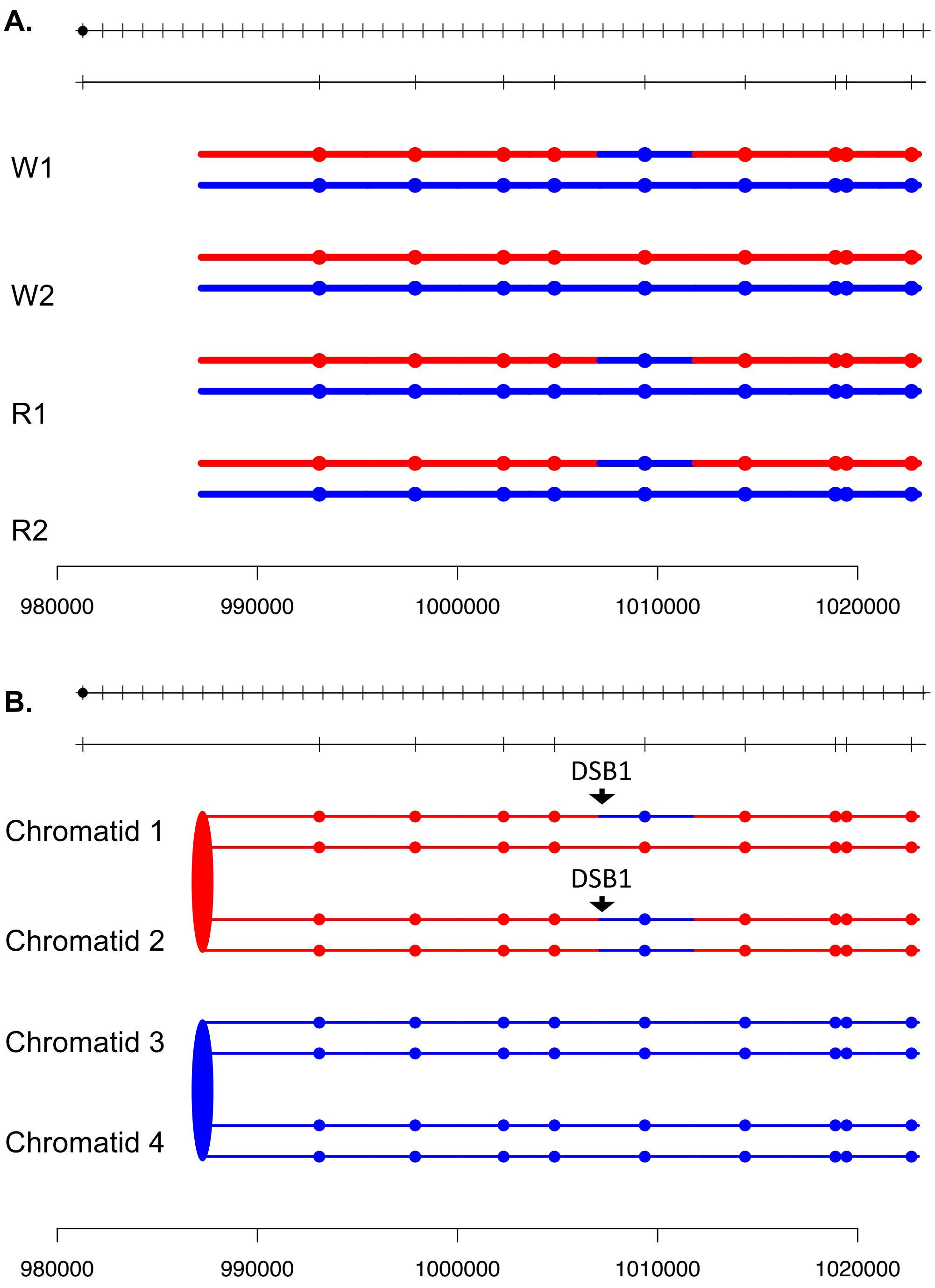
A.
W1
W2
R1
R2
980000
990000
1000000
1010000
1020000
B.
DSB1
DSB1
Chromatid 1
Chromatid 2
Chromatid 3
Chromatid 4
980000
990000
1000000
1010000
1020000

S55

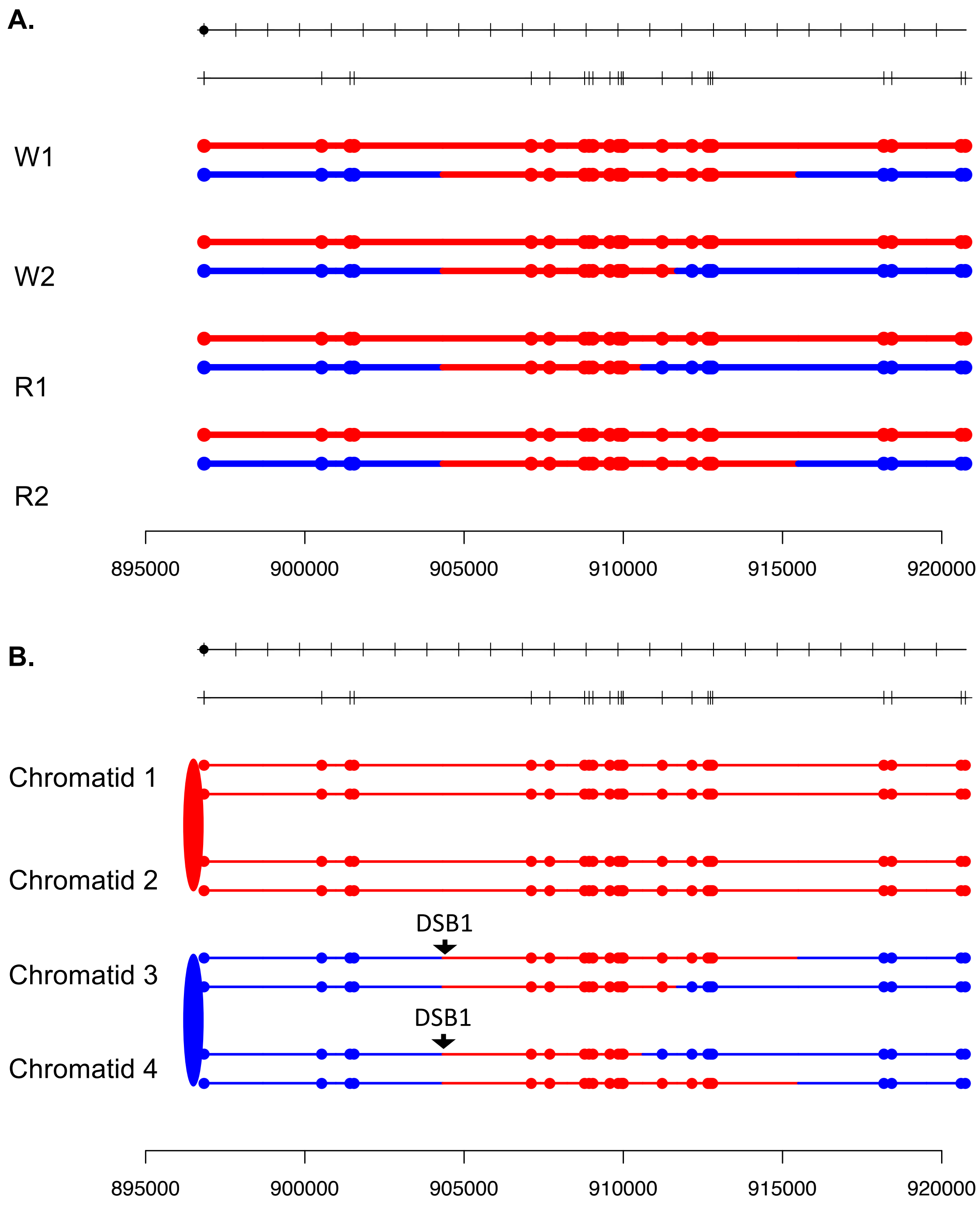
A.
W1
W2
R1
R2
895000
900000
905000
910000
915000
920000
B.
Chromatid 1
Chromatid 2
Chromatid 3
Chromatid 4
DSB1
DSB1
895000
900000
905000
910000
915000
920000

S56

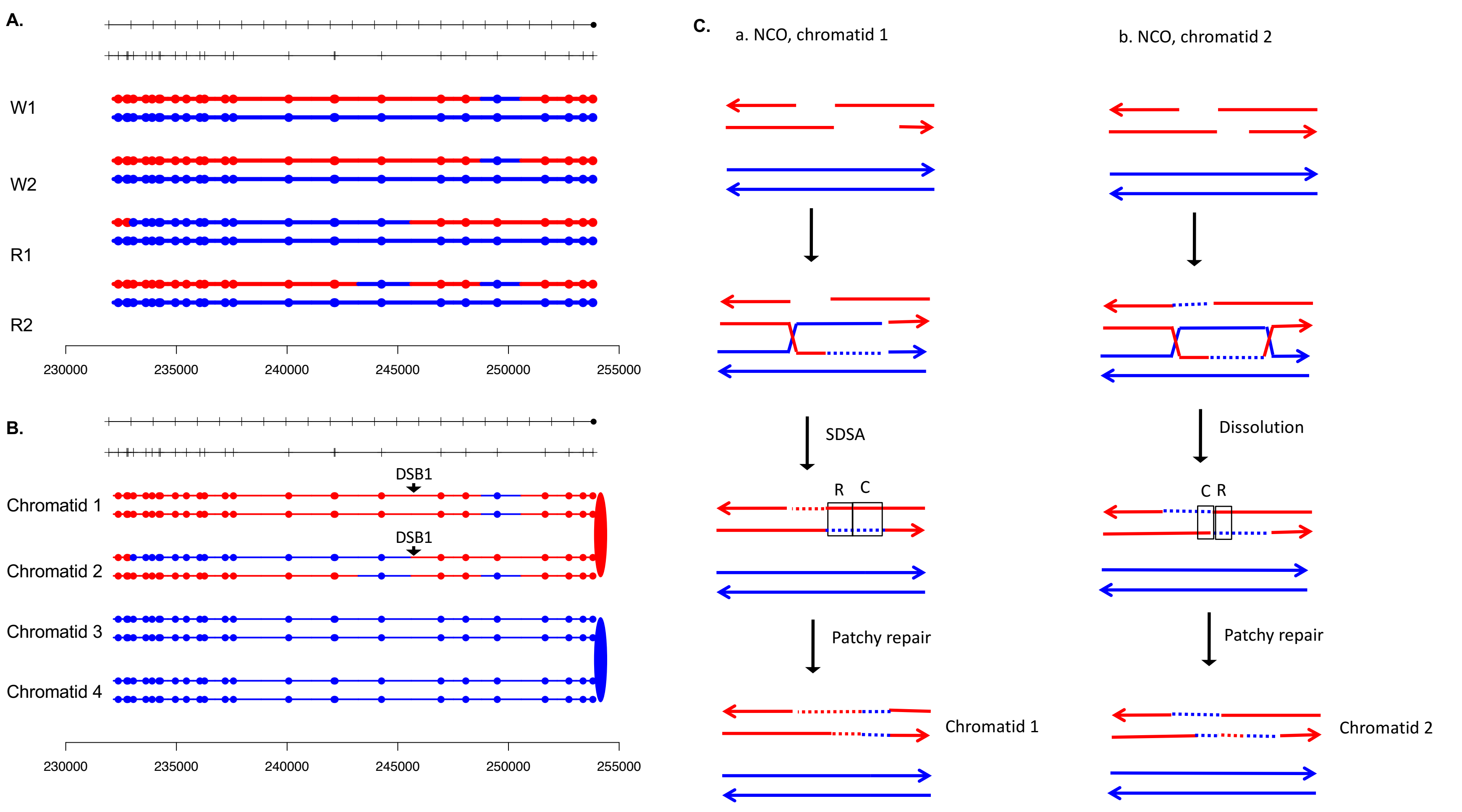

S56	NCO1	Conversion tract with no heteroduplex in chromatid 1	Mlh1-independent MMR
	NCO2	Conversion and restoration tracts in chromatid 2	Mlh1-independent patchy MMR

S57

A.

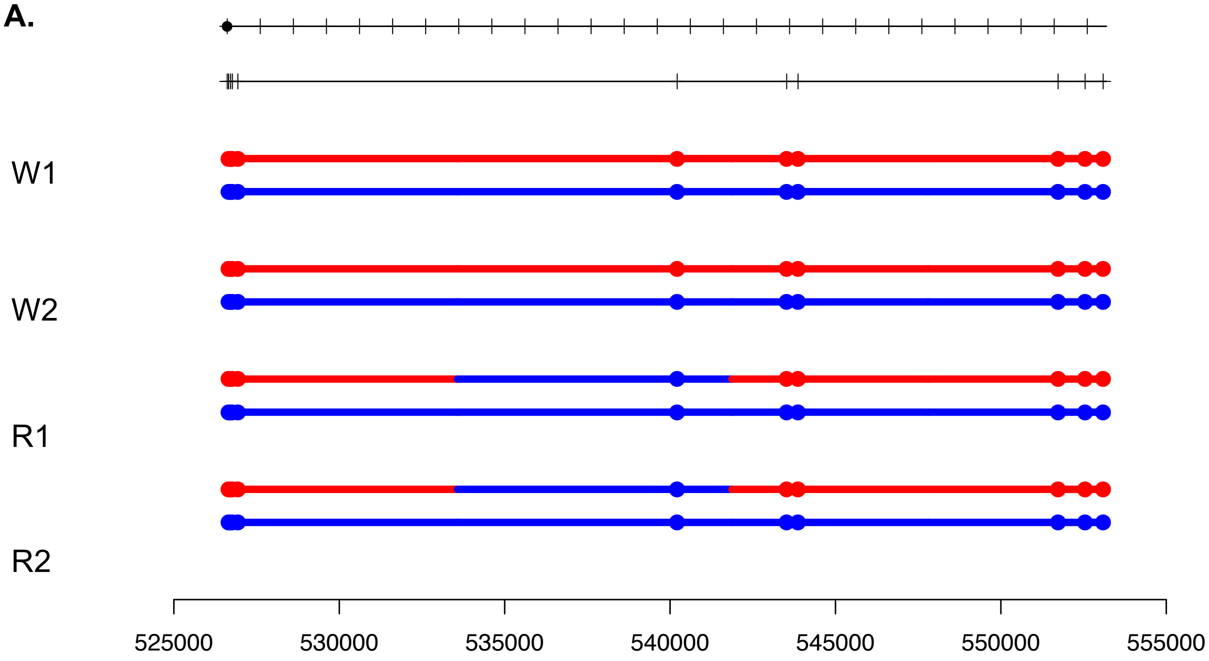

W1
W2
R1
R2
525000
530000
535000
540000
545000
550000
555000

B.

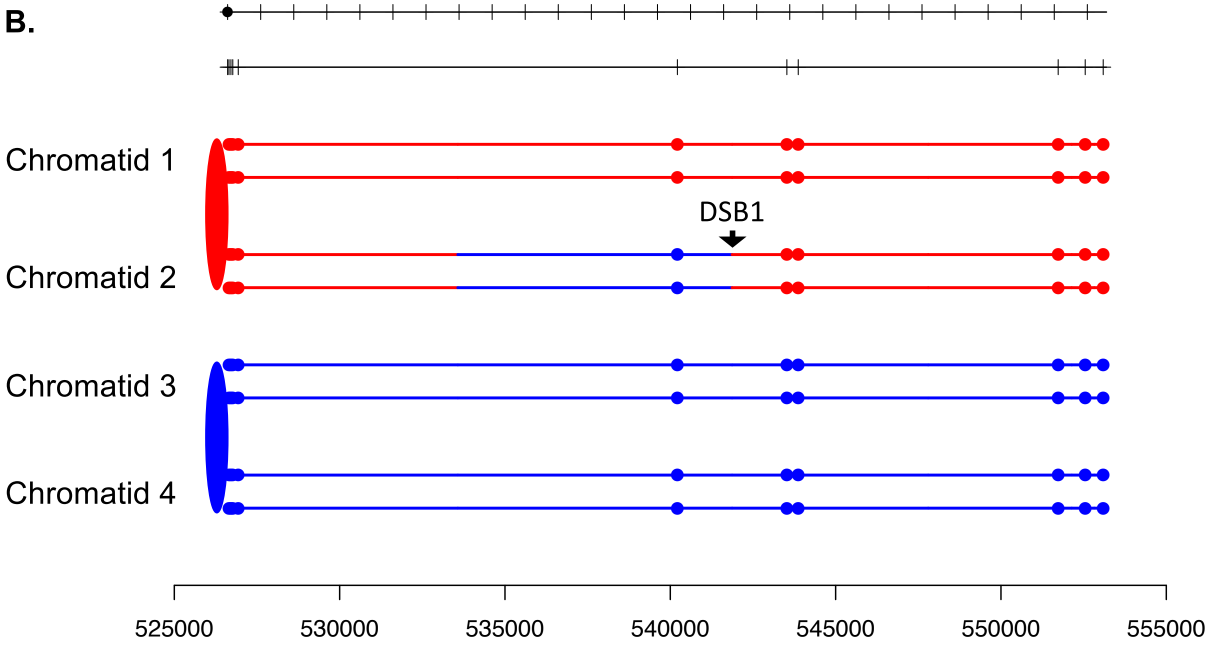

Chromatid 1
Chromatid 2
DSB1
Chromatid 3
Chromatid 4
525000
530000
535000
540000
545000
550000
555000

S58

A.

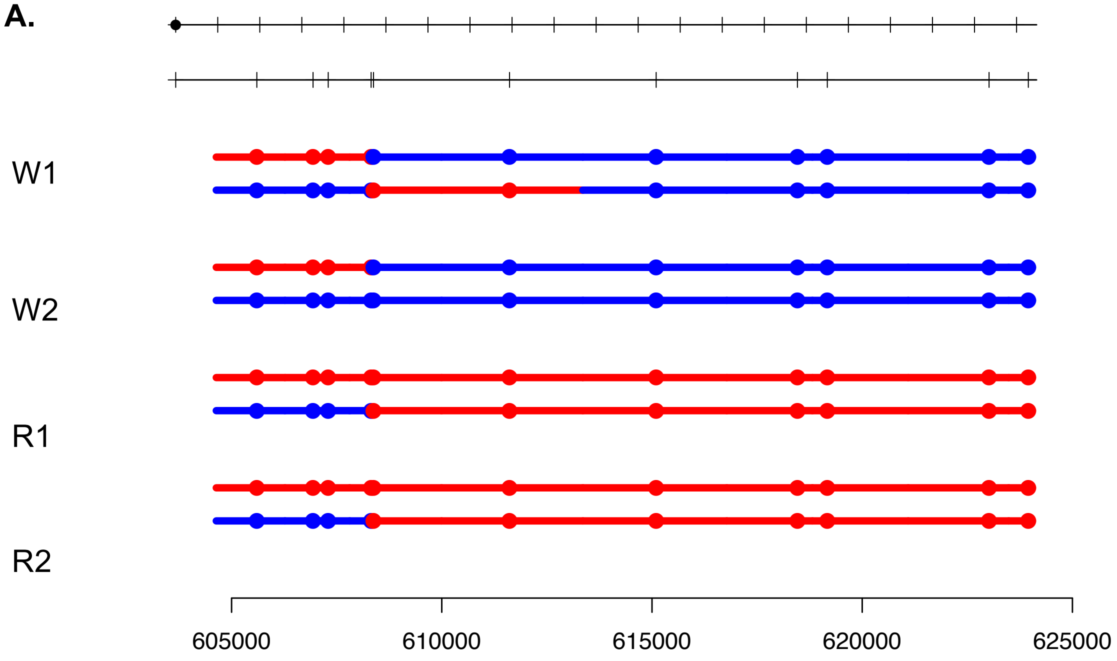
W1
W2
R1
R2
605000
610000
615000
620000
625000

B.

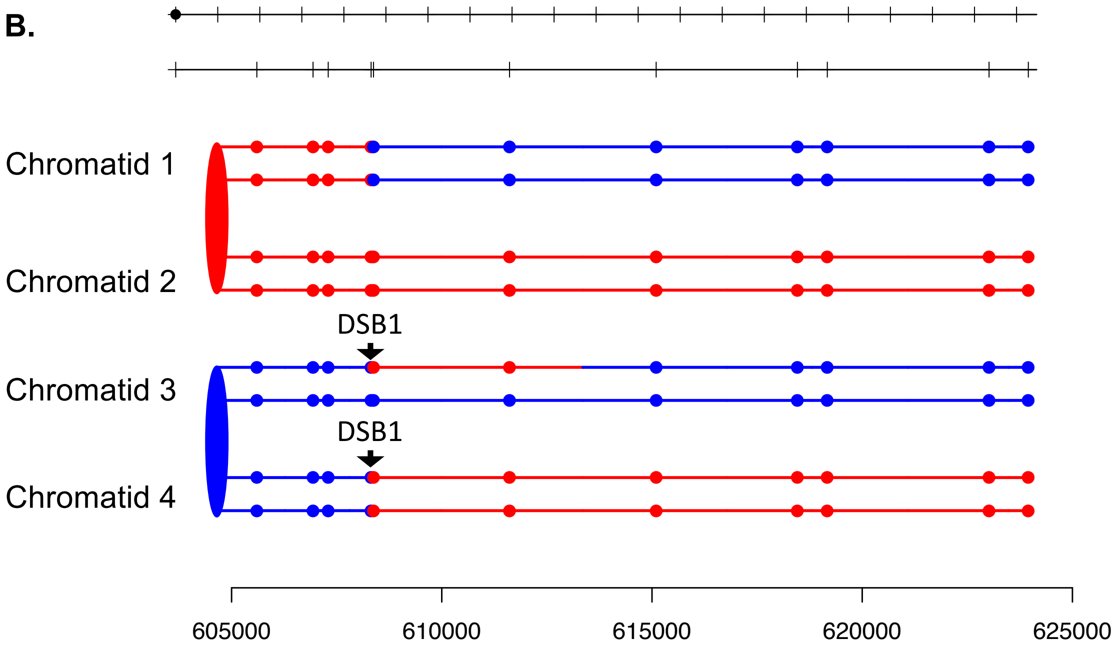
Chromatid 1
Chromatid 2
DSB1
Chromatid 3
DSB1
Chromatid 4
605000
610000
615000
620000
625000

S59

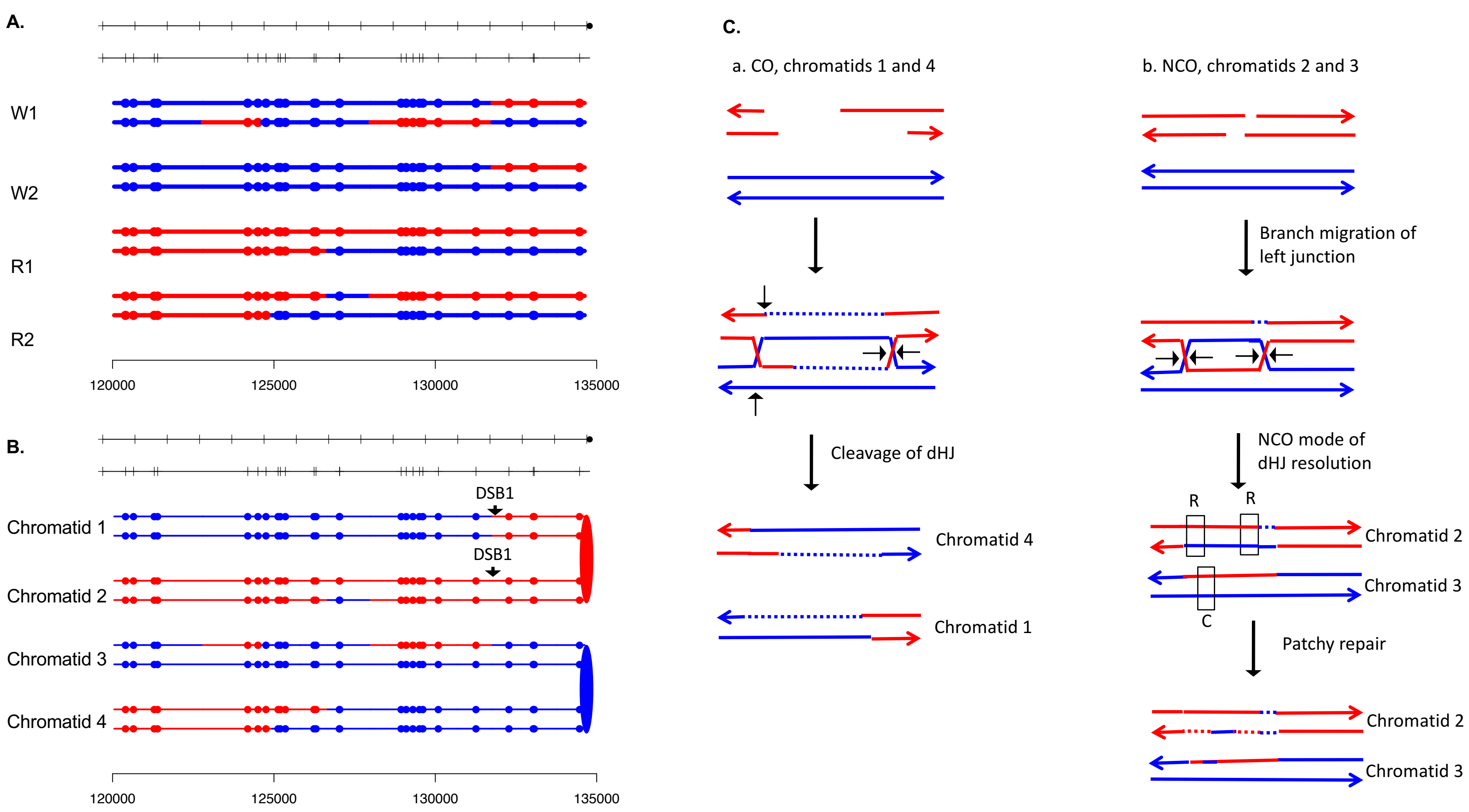

S59	CO	Long conversion tract between heteroduplex and putative DSB site on chromatid 4	Repair of double-stranded DNA gap
	NCO	Regions of conversion/restoration and heteroduplex on same side of putative DSB site in chromatids 3 and 4	Branch migration; NCO resolution of dHJ; Mlh1-independent patchy repair

S60

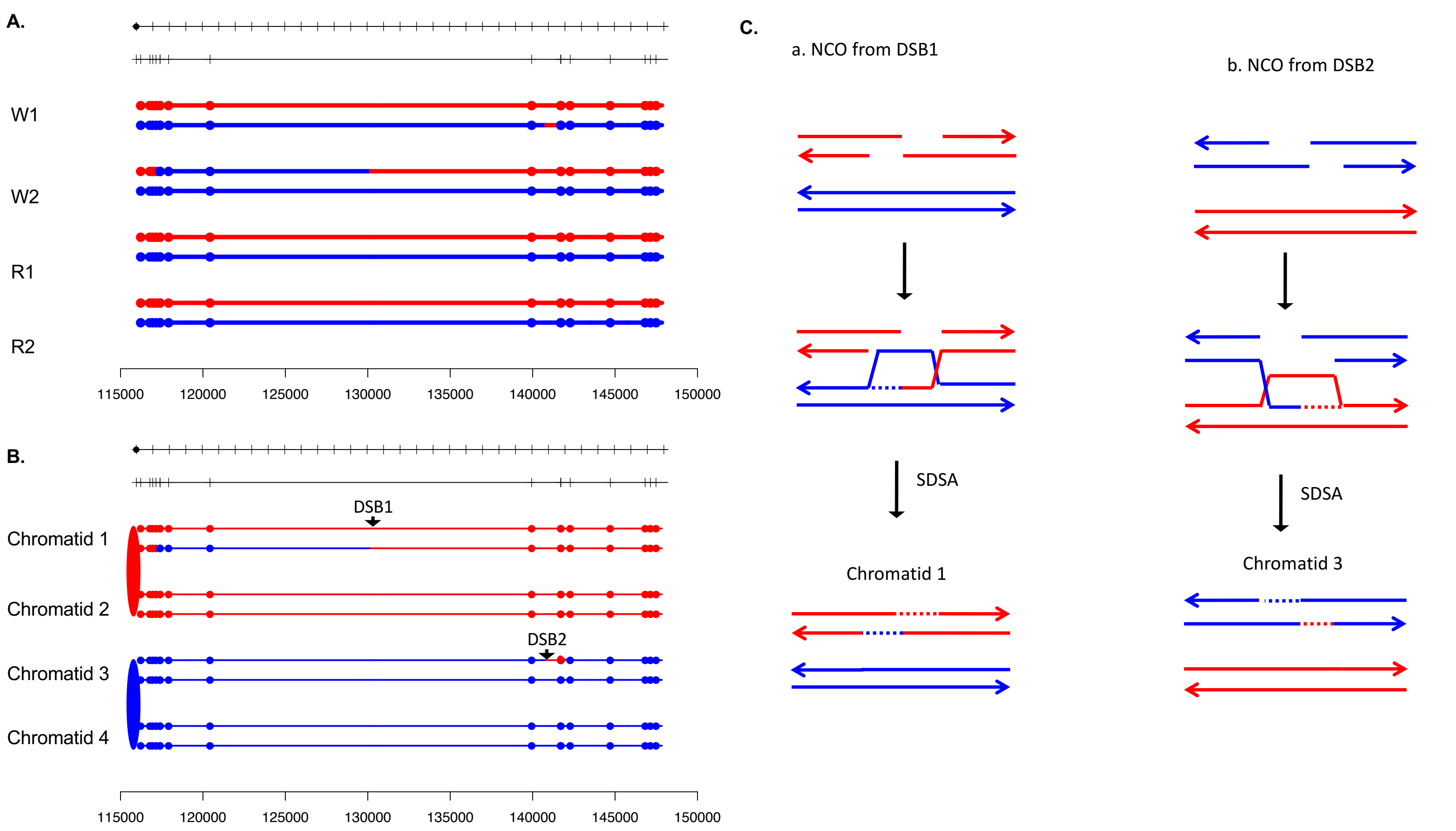

S60	NCO1	Simple heteroduplex. DSB on red chromatid.	SDSA
	NCO2	Simple heteroduplex. DSB on blue chromatid.	SDSA

S61

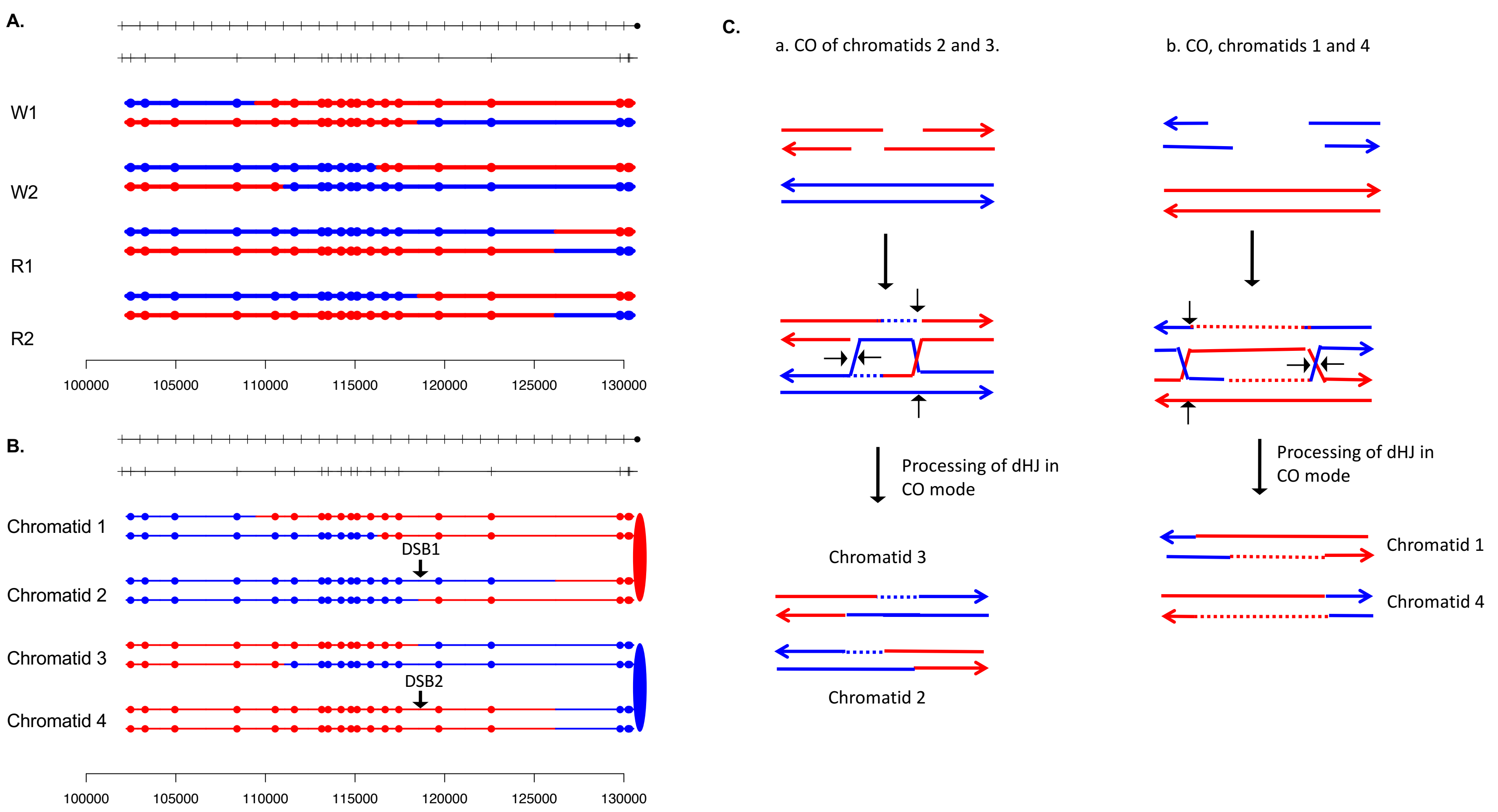

S61	CO1	Uni-directional heteroduplexes on chromatids 2 and 3 propagated in opposite directions	Crossover by standard DSBR model
	CO2	DSB on blue chromatid, large conversion tract	Repair of double-stranded DNA gap; followed by cleavage of dHJ to yield a CO

S62

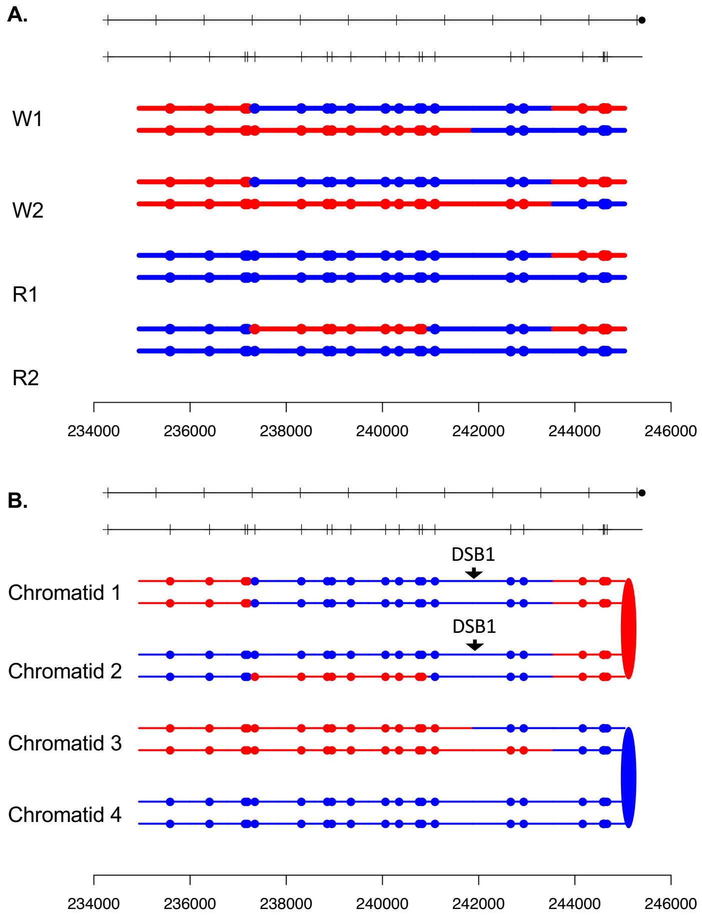

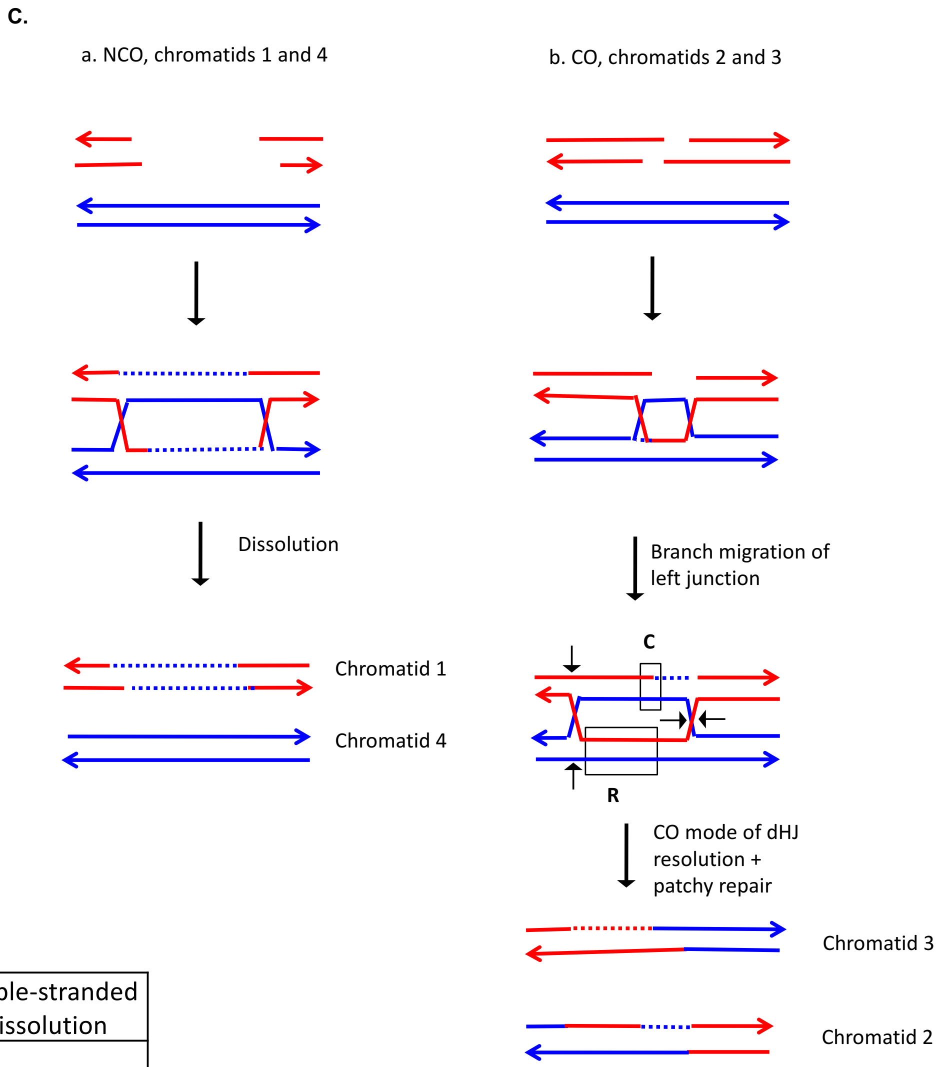

S62	NCO	Conversion tract spanning putative DSB site in chromatid 1	Repair of double-stranded DNA gap; dissolution
	CO	Uni-directional heteroduplexes on chromatids 2 and 3; propagated in opposite directions; interspersed conversion and restoration tracts	Mlh1-independent MMR

S63

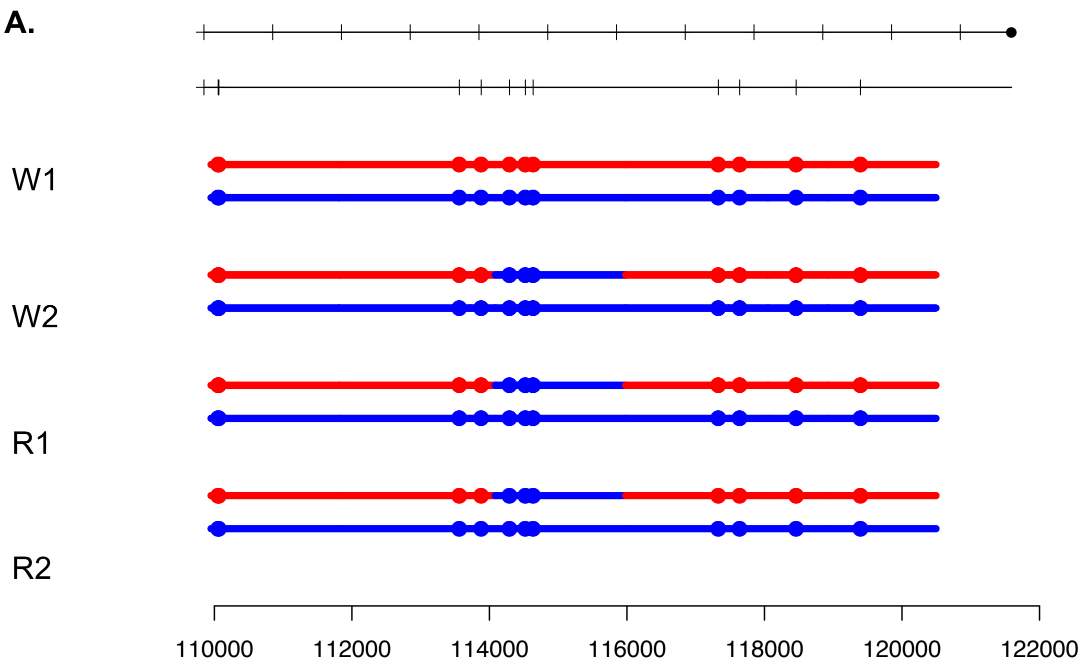

A.
W1
W2
R1
R2
110000
112000
114000
116000
118000
120000
122000

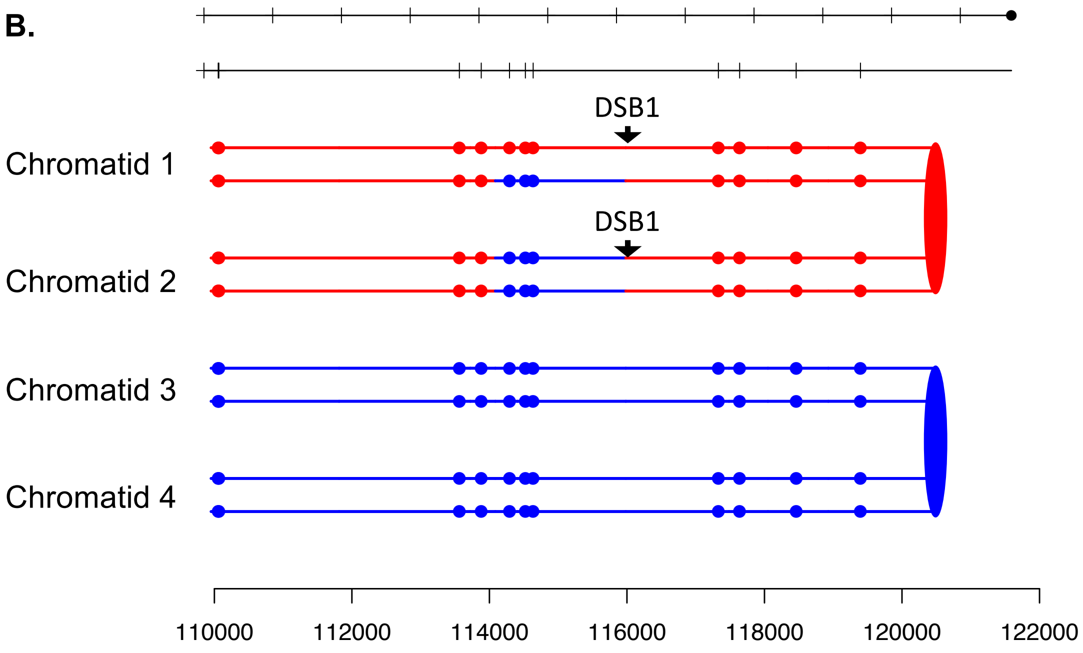

B.
DSB1
Chromatid 1
DSB1
Chromatid 2
Chromatid 3
Chromatid 4
110000
112000
114000
116000
118000
120000
122000

S64

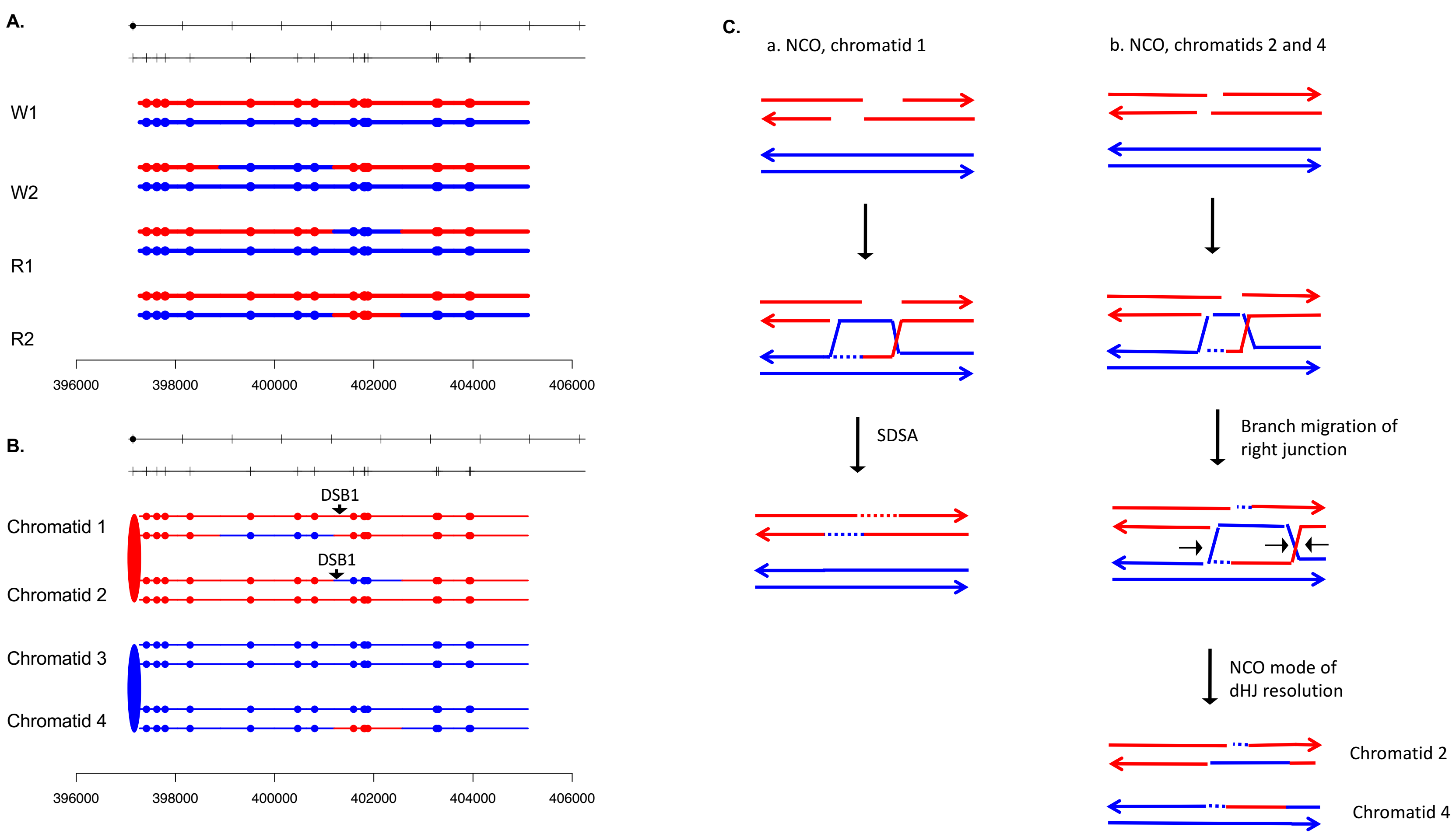

S64	NCO1	Simple heteroduplex (chromatid 1)	SDSA
	NCO2	Heteroduplexes on same side of DSB in chromatids 2 and 4	Branch migration; NCO mode of dHJ resolution

S65

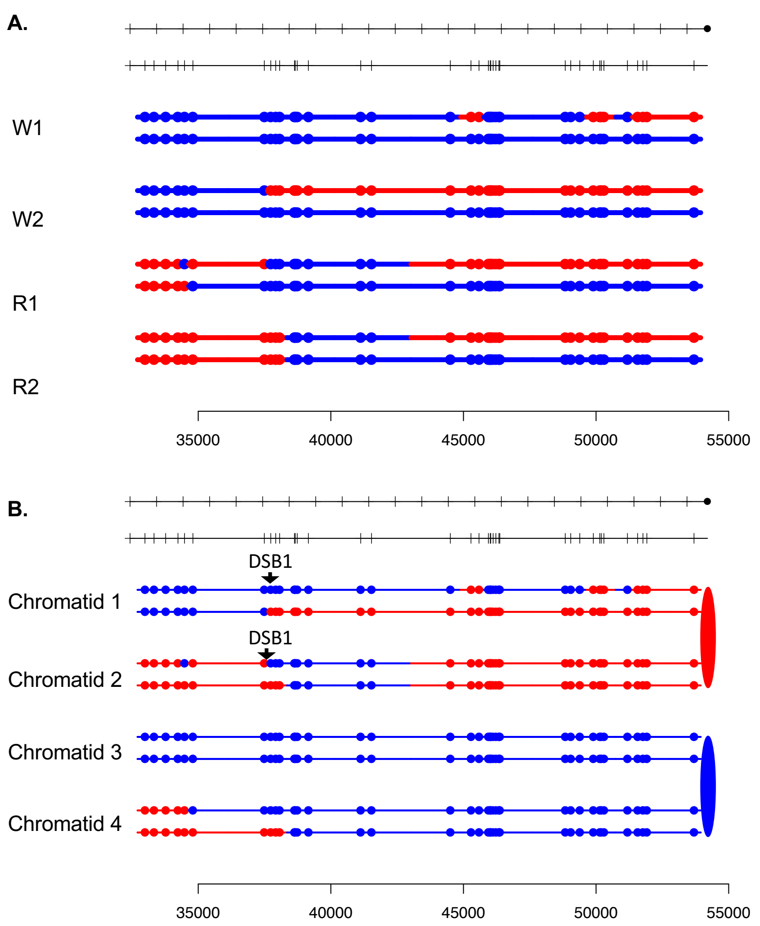

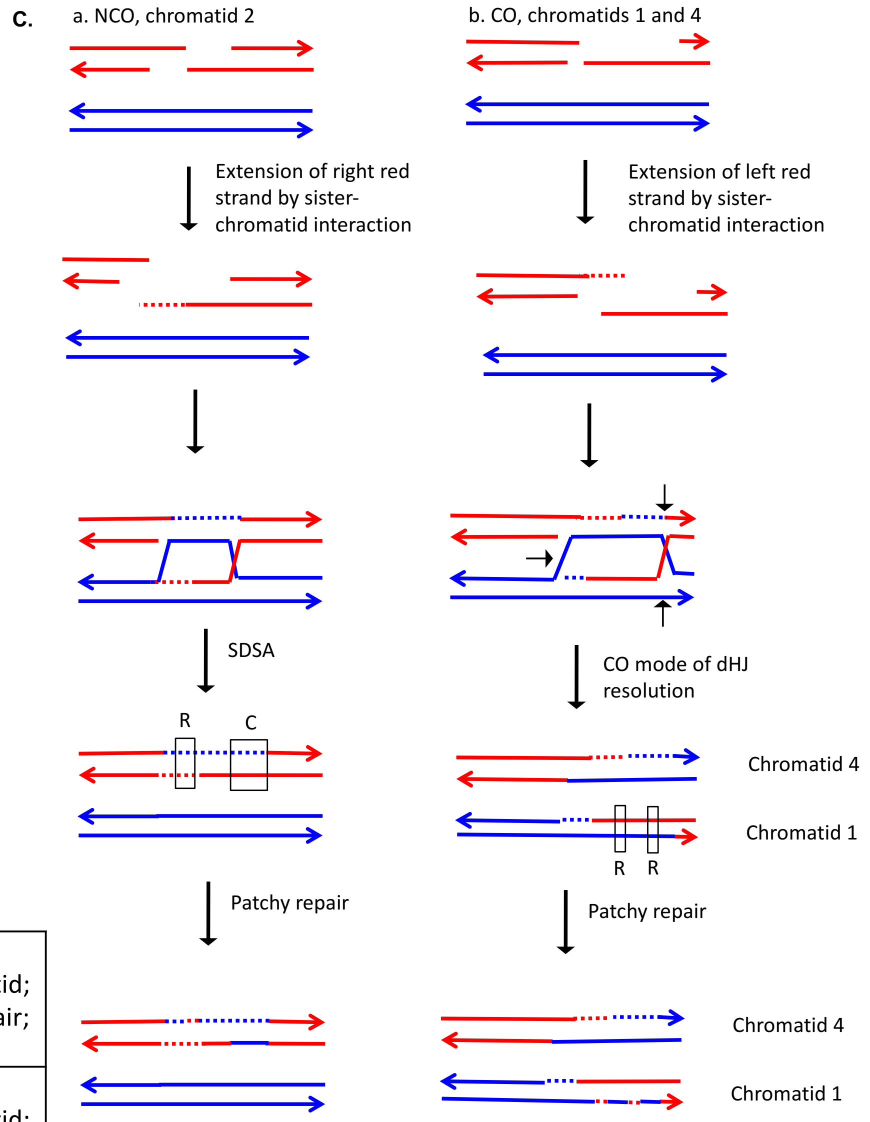

S65	CO	Heteroduplex on same side of DSB site in chromatids 1 and 4; heteroduplex spans DSB site in chromatid 4; tracts of restoration within heteroduplex in chromatid 2	Extension of broken end by interaction with sister chromatid; Mlh1-independent patchy repair; CO resolution of dHJ
	NCO	Heteroduplex spans DSB site in chromatid 2; tracts of conversion and restoration interspersed with heteroduplex regions	Extension of broken end by interaction with sister chromatid; Mlh1-independent patchy repair; SDSA

S66

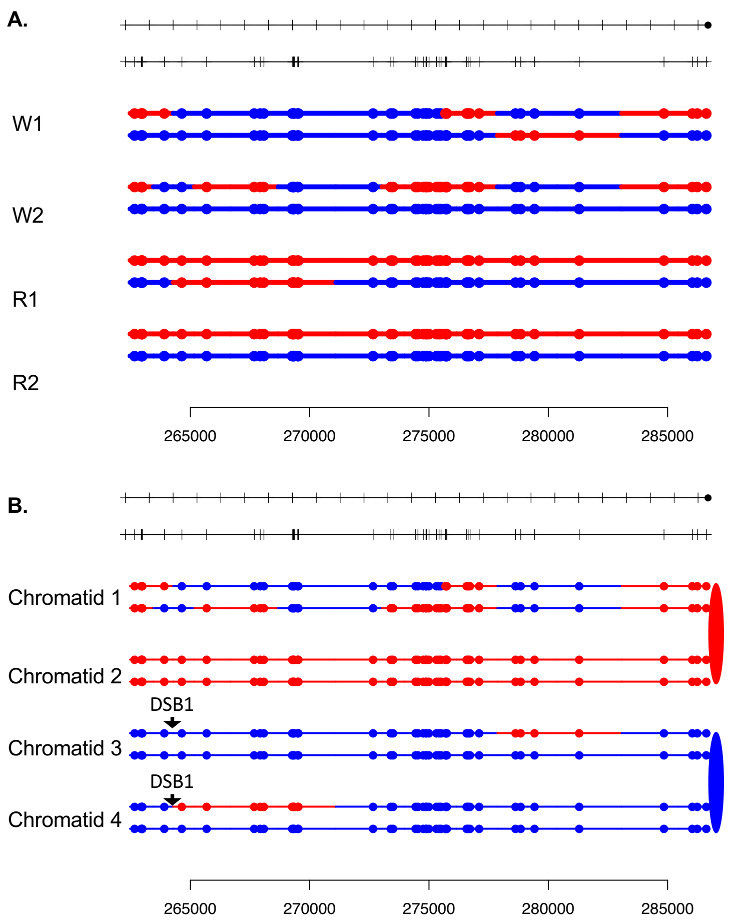

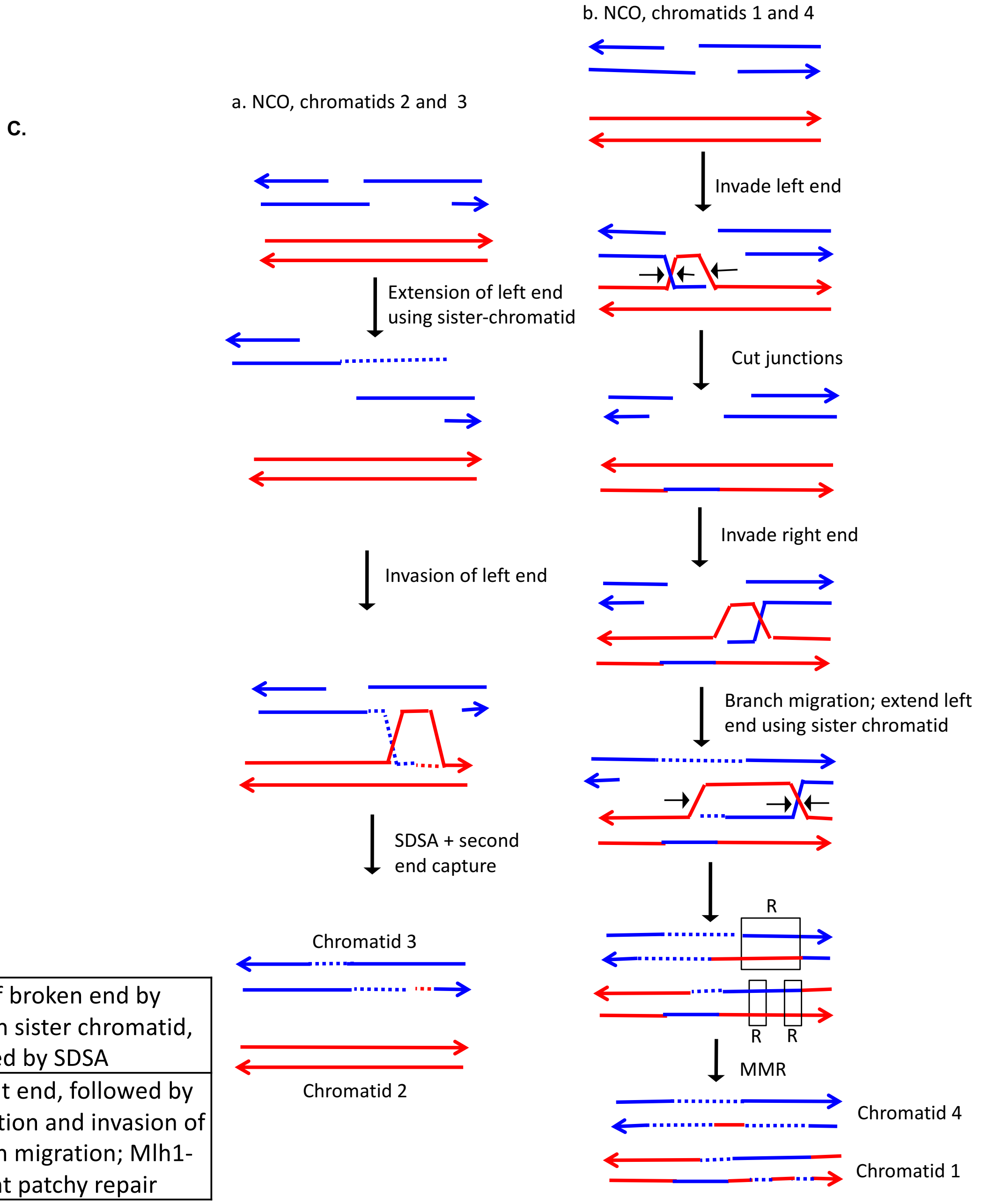

S66	NCO1	Heteroduplex on chromatid 3 separated from putative DSB site by long region of homoduplex	Extension of broken end by interaction with sister chromatid, followed by SDSA
	NCO2	Heteroduplexes on both chromatids 1 and 4; chromatid 1 has trans heteroduplexes flanking the DSB site in addition to regions of homoduplex within the heteroduplex	Invasion of right end, followed by cleavage of junction and invasion of left end; branch migration; Mlh1-independent patchy repair

S67

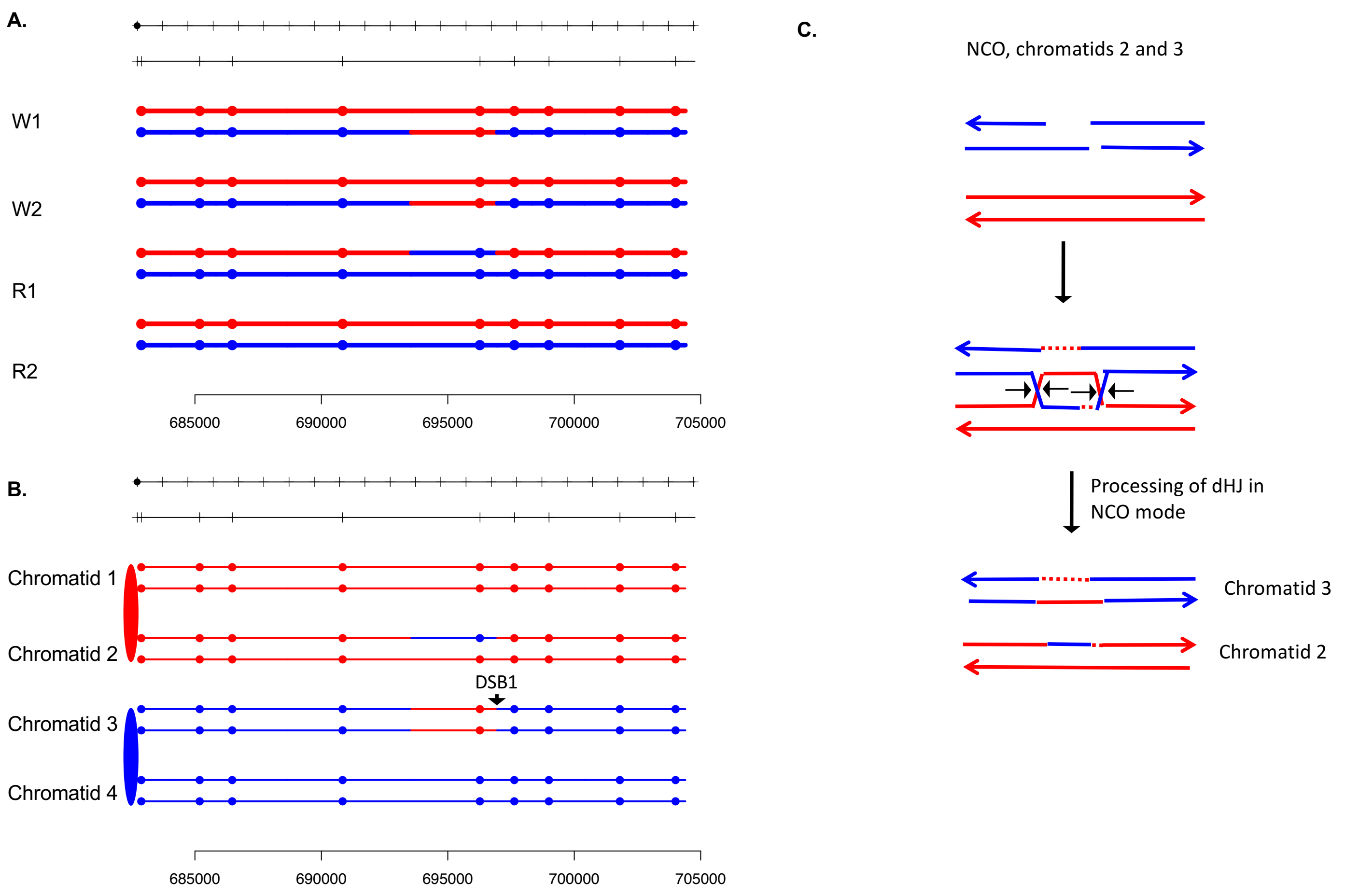

S67	NCO	Uni-directional heteroduplexes on chromatids 2 and 3 propagated in opposite directions	NCO mode of dHJ resolution with limited synthesis

S68

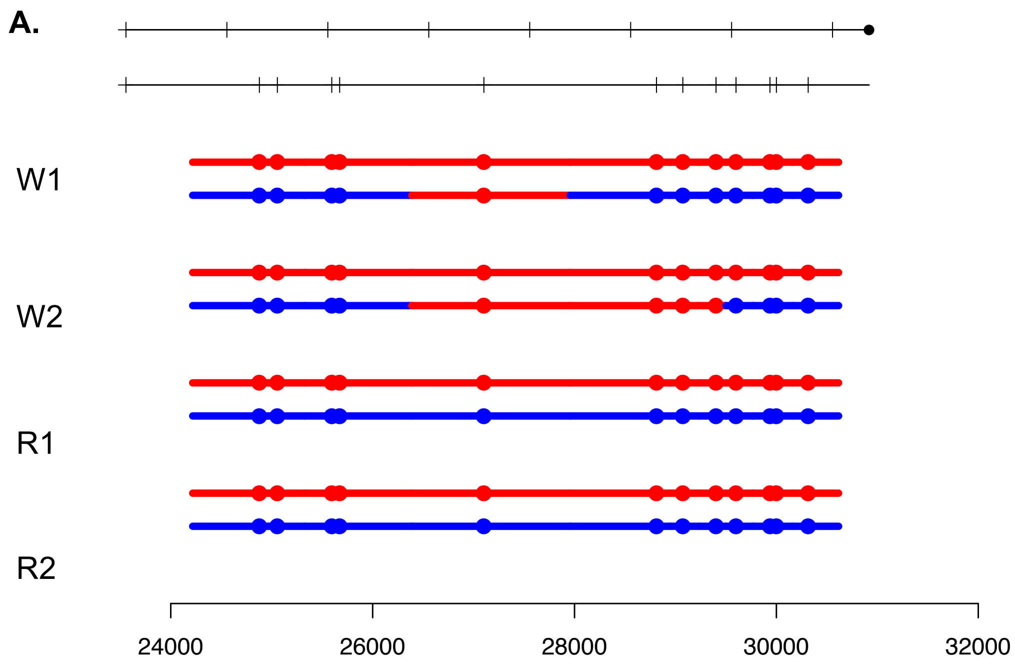
A.
W1
W2
R1
R2
24000
26000
28000
30000
32000

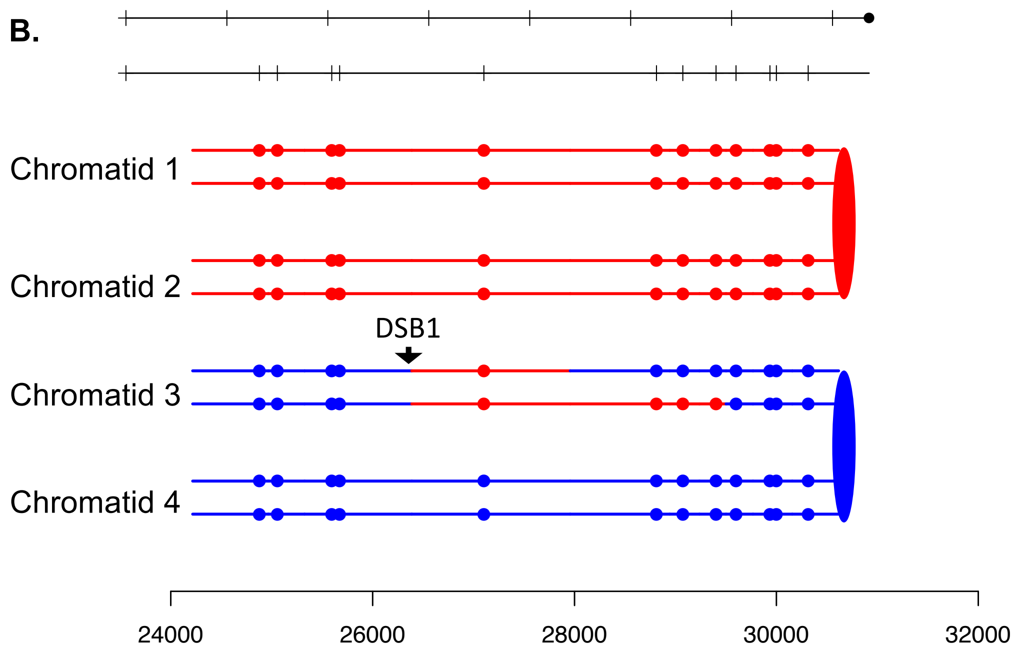
B.
Chromatid 1
Chromatid 2
DSB1
Chromatid 3
Chromatid 4
24000
26000
28000
30000
32000

S69

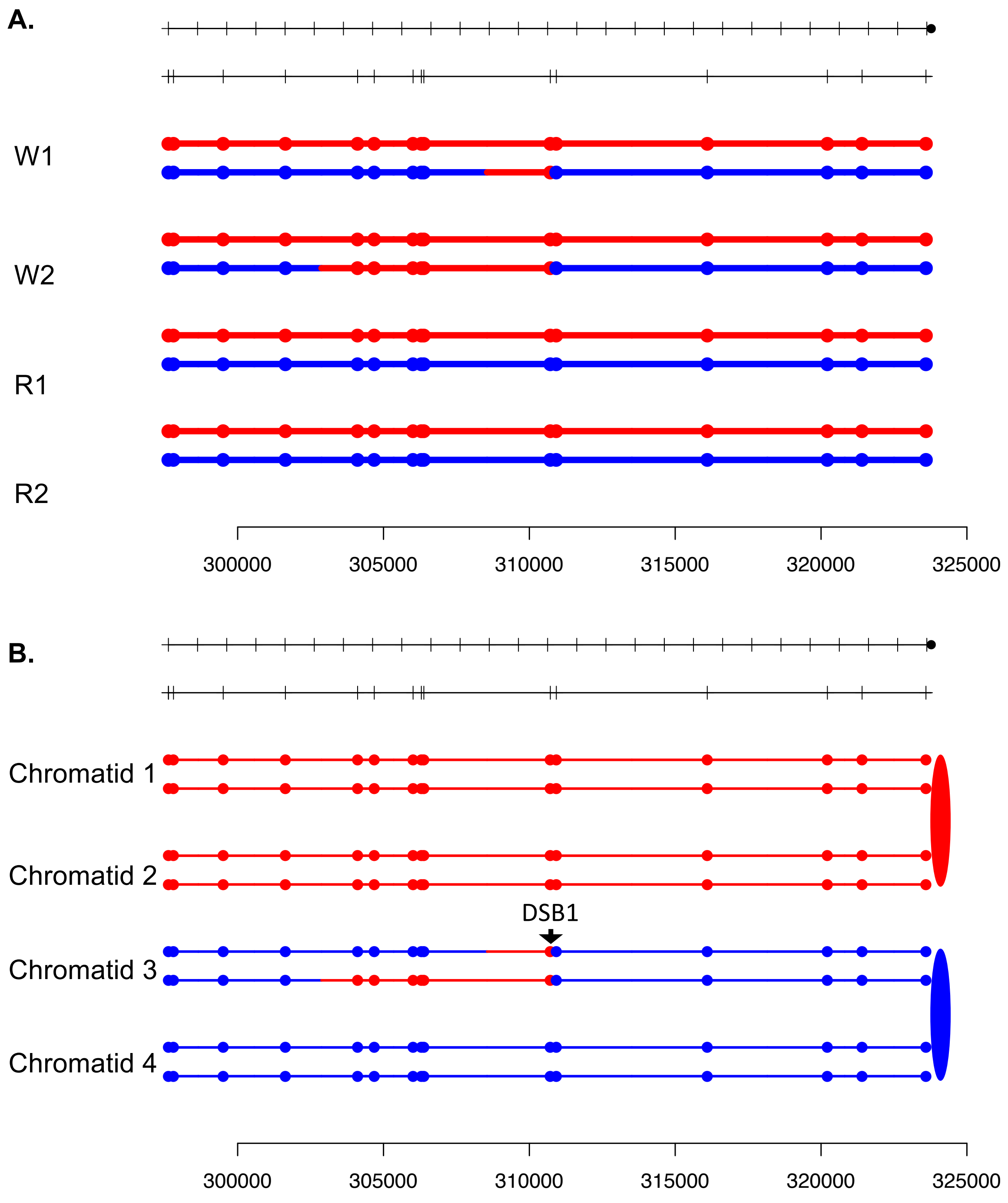
A.
W1
W2
R1
R2
300000
305000
310000
315000
320000
325000
B.
Chromatid 1
Chromatid 2
DSB1
Chromatid 3
Chromatid 4
300000
305000
310000
315000
320000
325000

S70

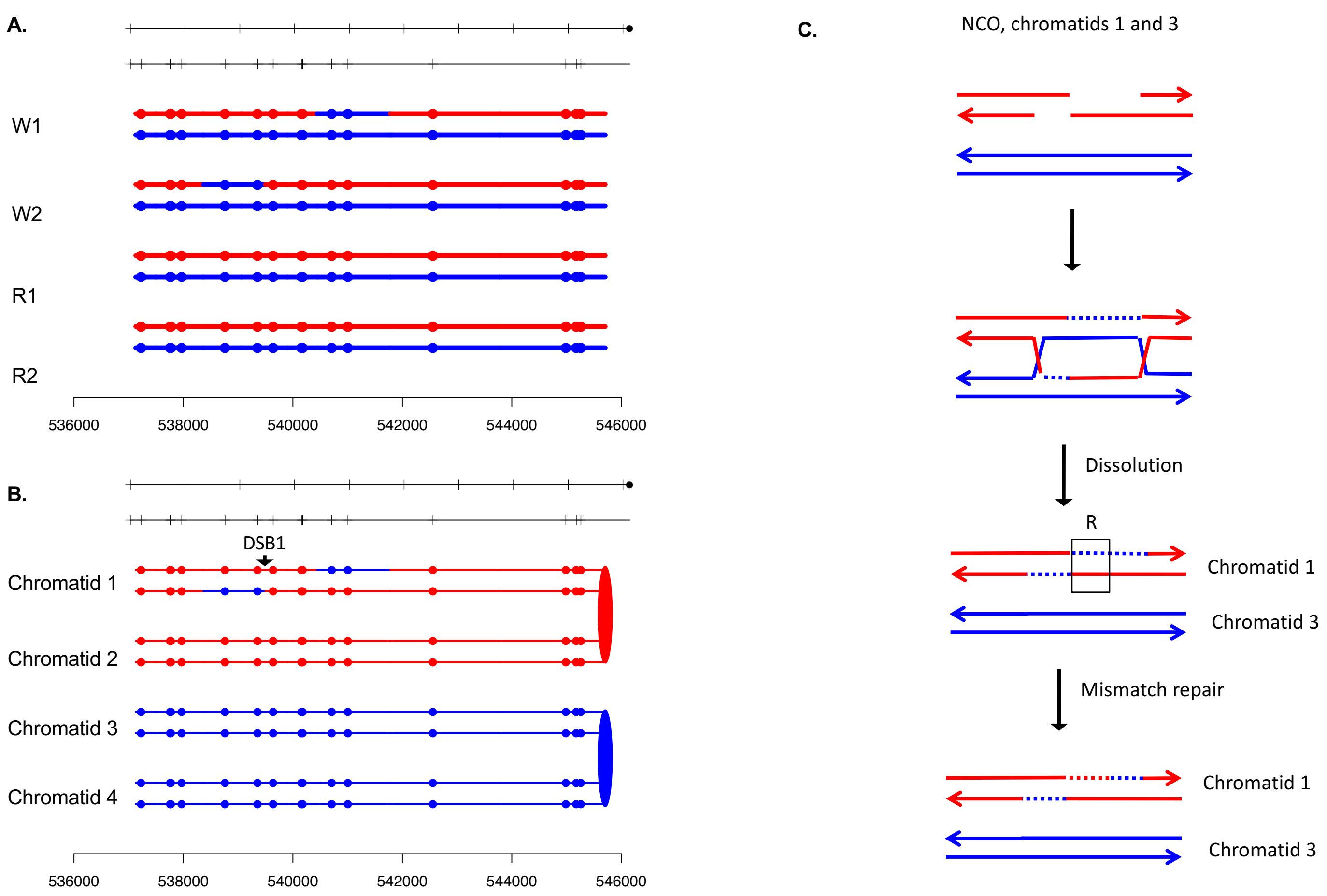

S70	NCO	*Trans* heteroduplexes separated by restoration tract on chromatid 1	Formation of dHJ event; dissolution followed by Mlh1-independent repair

S71

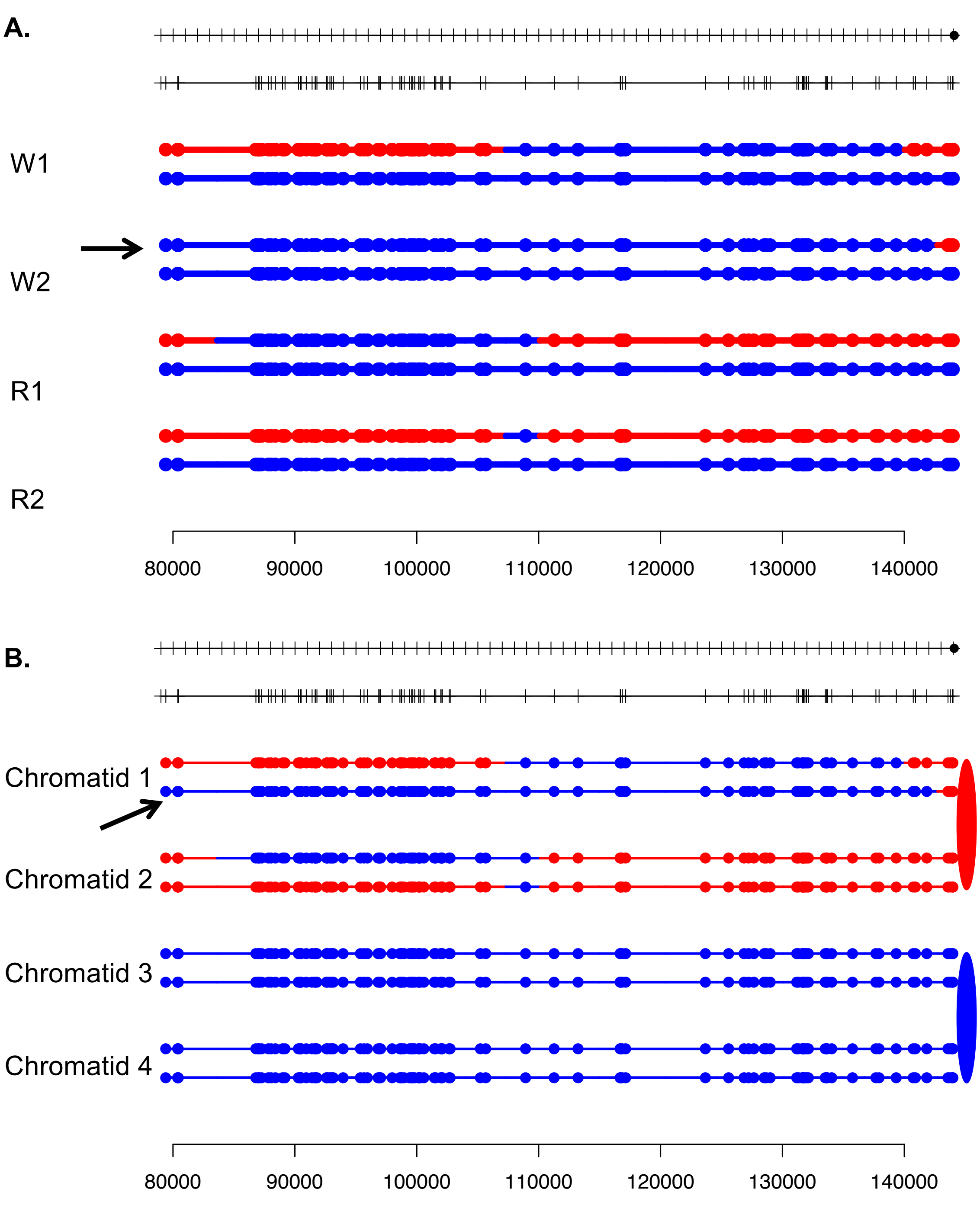
A.
W1
W2
R1
R2
80000
90000
100000
110000
120000
130000
140000
B.
Chromatid 1
Chromatid 2
Chromatid 3
Chromatid 4
80000
90000
100000
110000
120000
130000
140000

S72

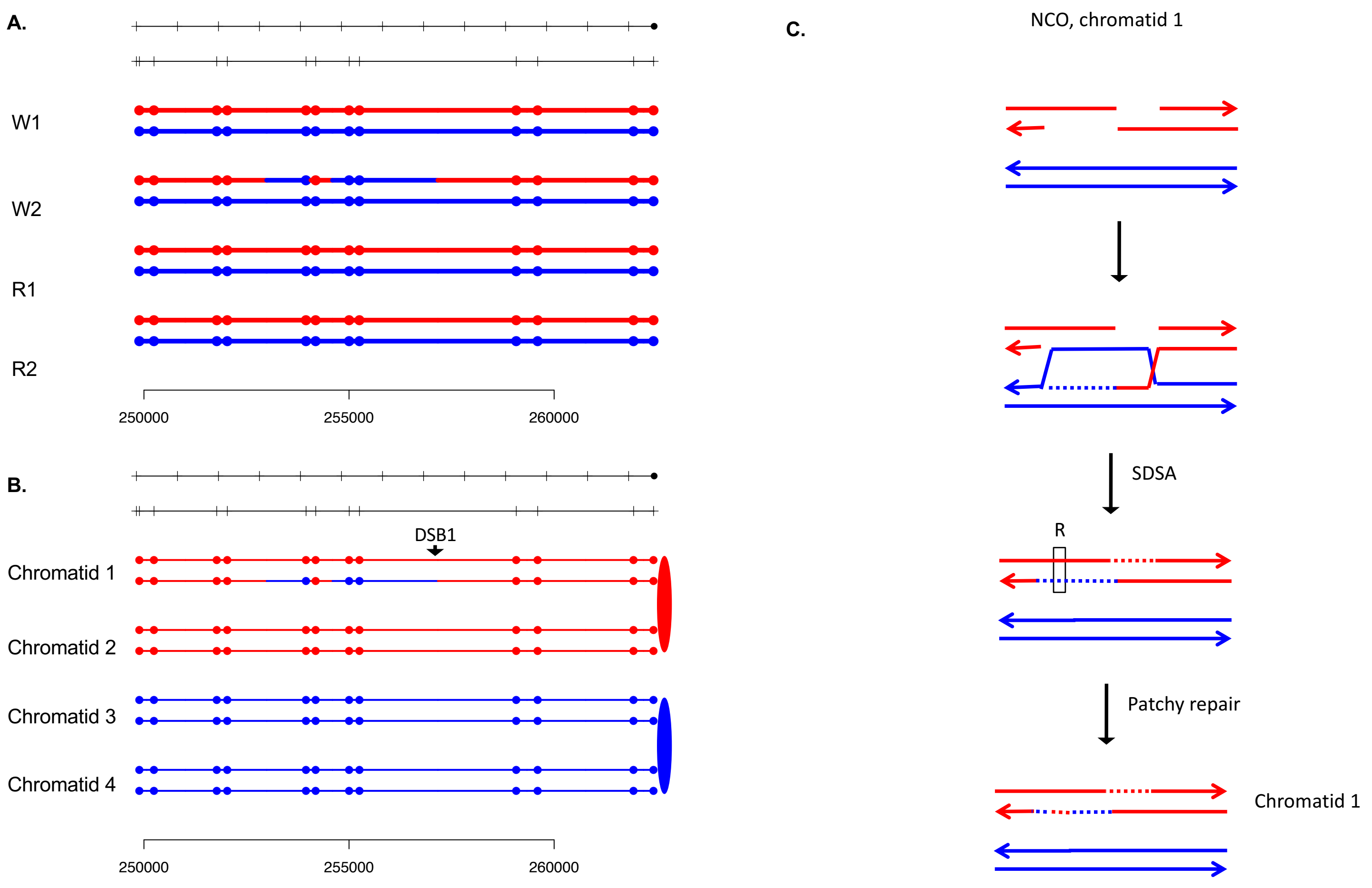

S72	NCO	Heteroduplex tract with an internal restoration tract	Mlh1-independent MMR, SDSA

S73

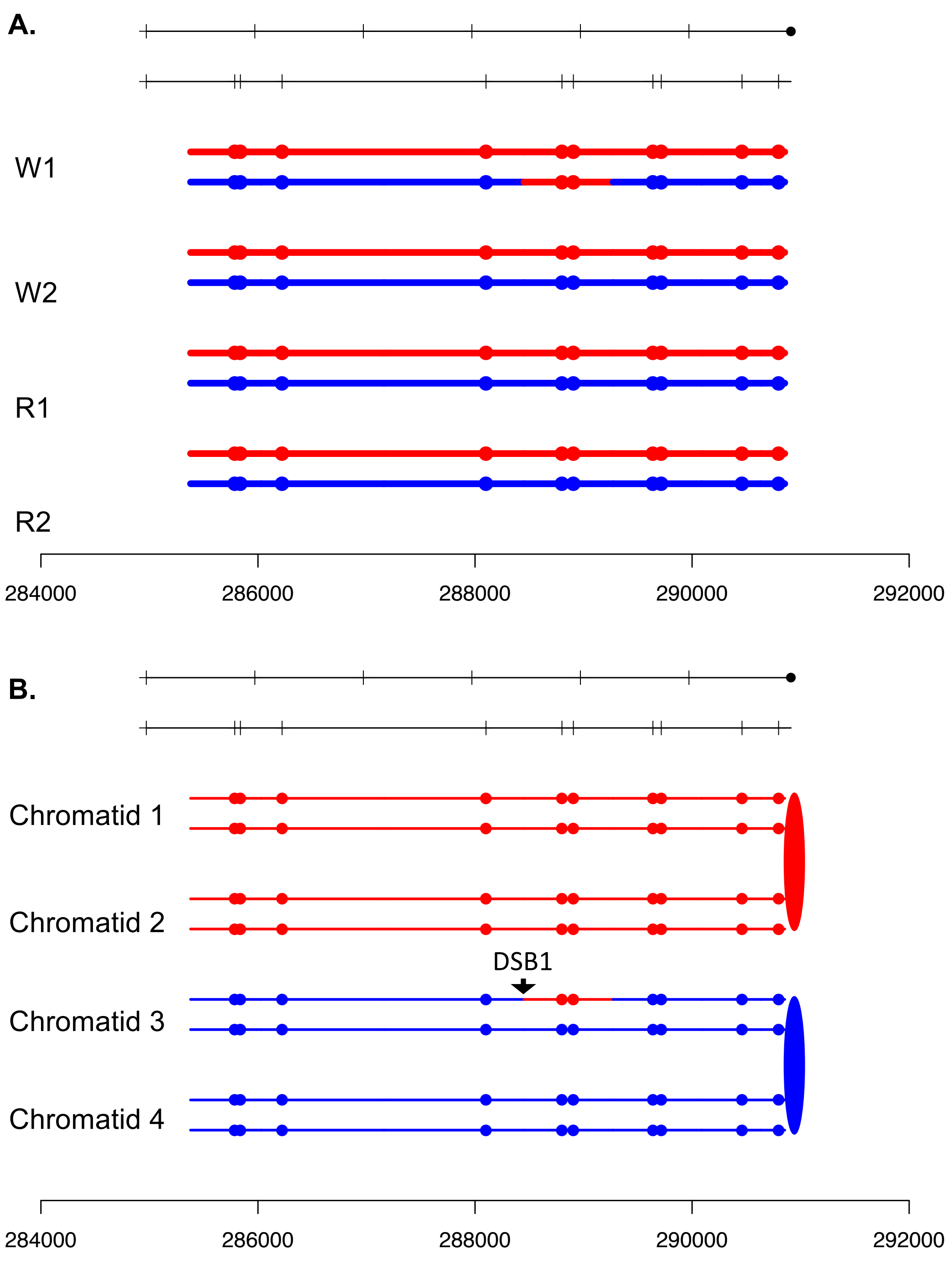
A.
W1
W2
R1
R2
284000
286000
288000
290000
292000
B.
Chromatid 1
Chromatid 2
DSB1
Chromatid 3
Chromatid 4
284000
286000
288000
290000
292000

S74

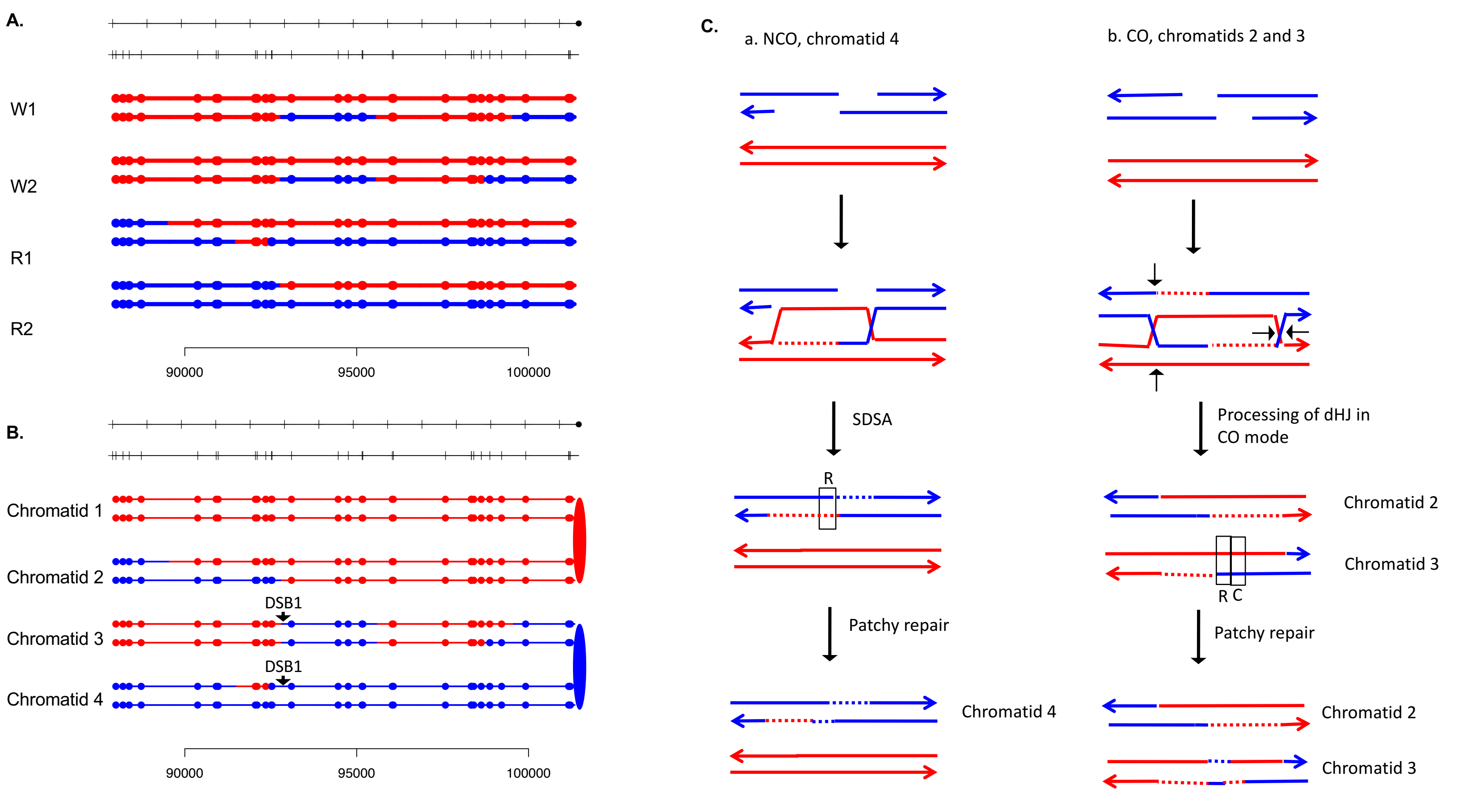

S74	NCO	Region of restoration repair separating DSB site from heteroduplex on chromatid 4	Mlh1-independent MMR, SDSA
	CO	Long regions of restoration and conversion repair separating DSB site from heteroduplex tract	Mlh1-independent MMR or template switching

S75

A.

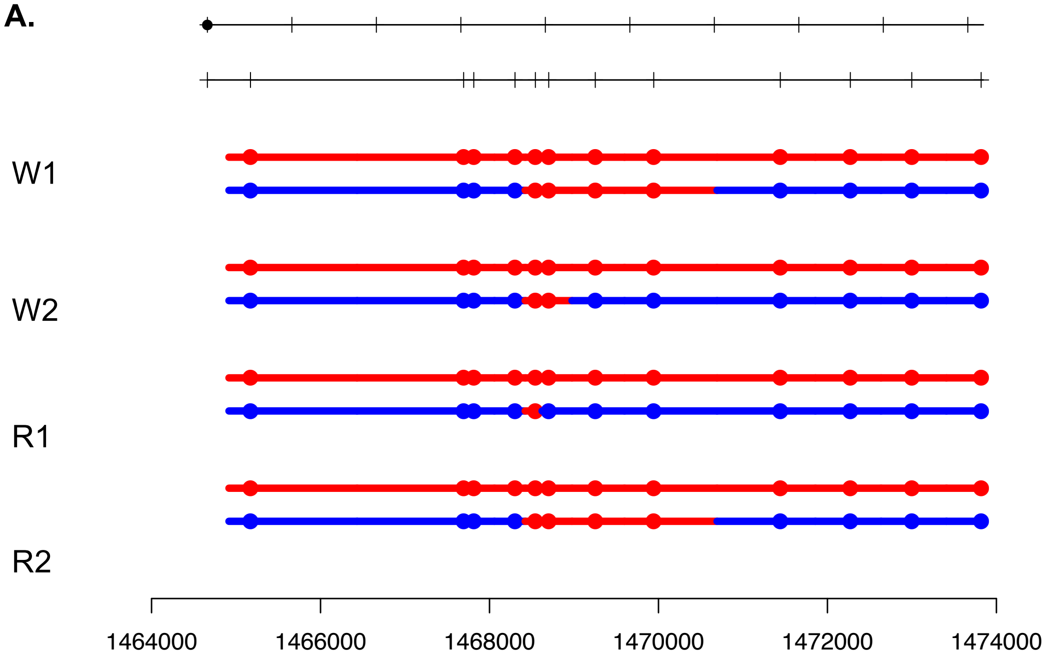
W1
W2
R1
R2
1464000
1466000
1468000
1470000
1472000
1474000

B.

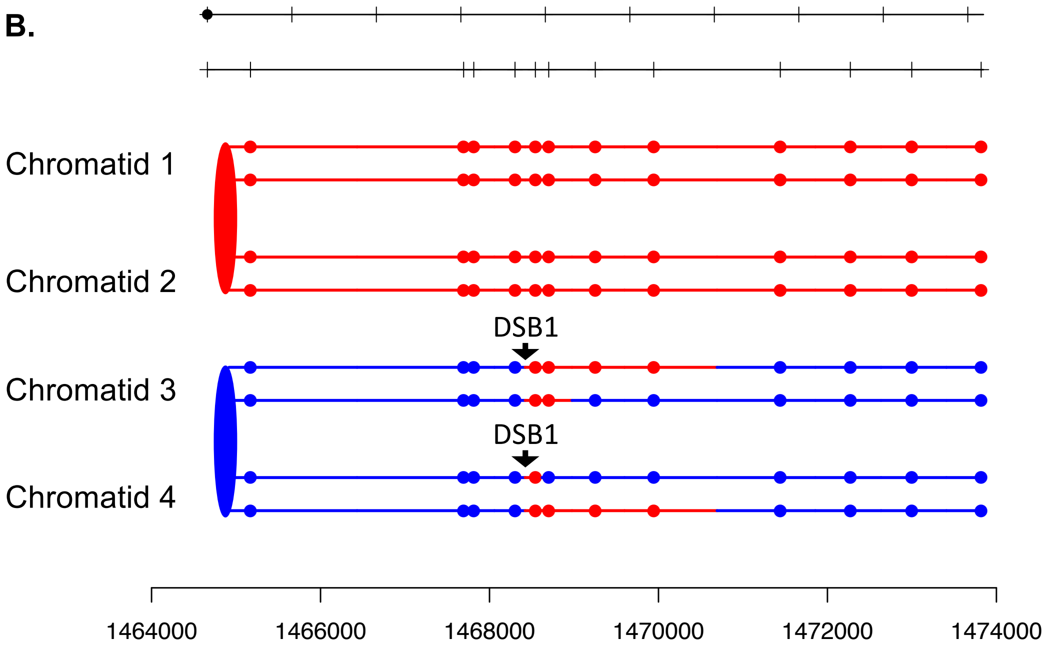
Chromatid 1
Chromatid 2
DSB1
Chromatid 3
DSB1
Chromatid 4
1464000
1466000
1468000
1470000
1472000
1474000

S76

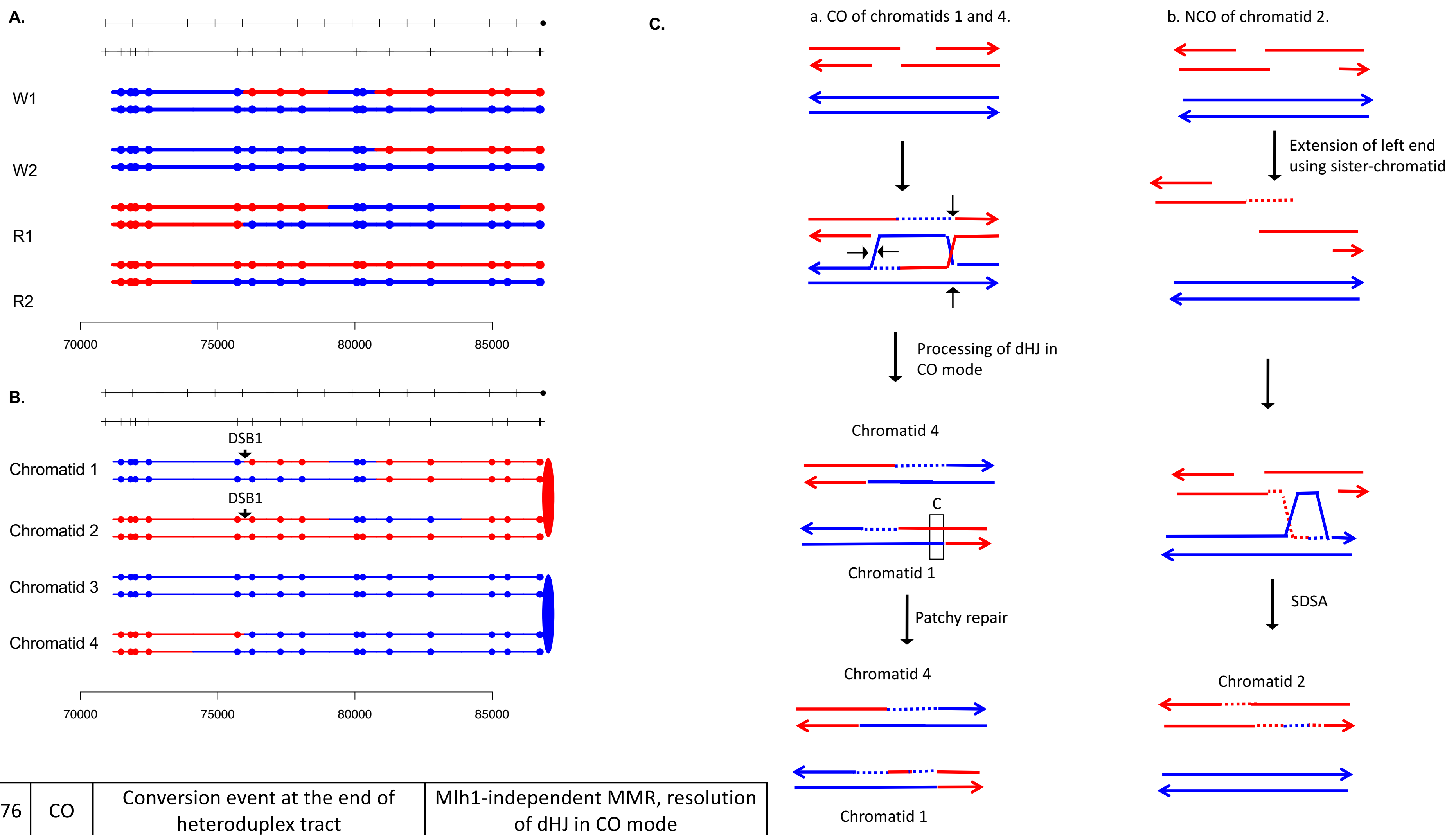

S76	CO	Conversion event at the end of heteroduplex tract	Mlh1-independent MMR, resolution of dHJ in CO mode
	NCO	Displacement of heteroduplex tract from DSB site	Interaction of broken end with sister chromatid or long restoration tract by Mlh1-independent MMR

S77

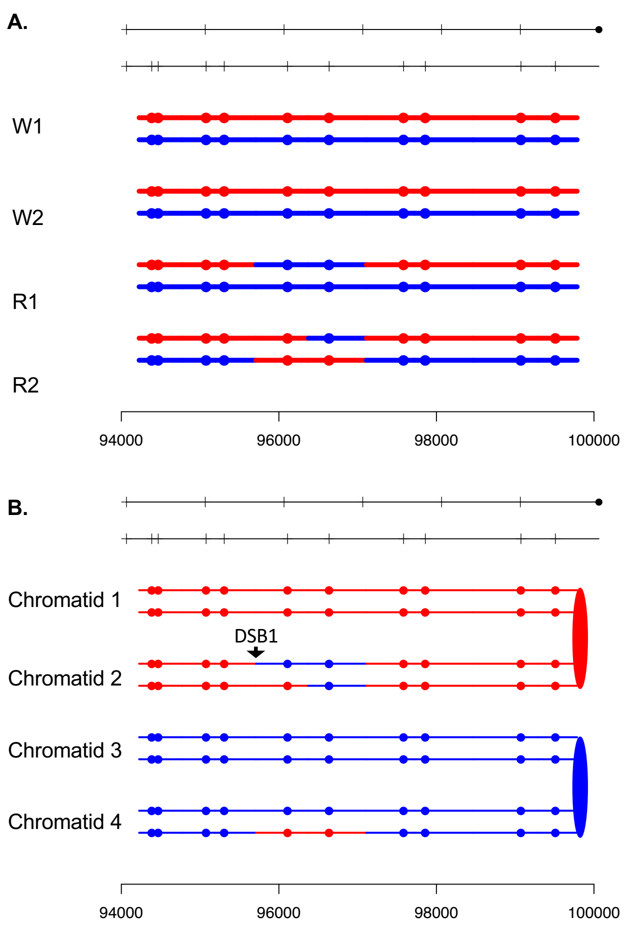

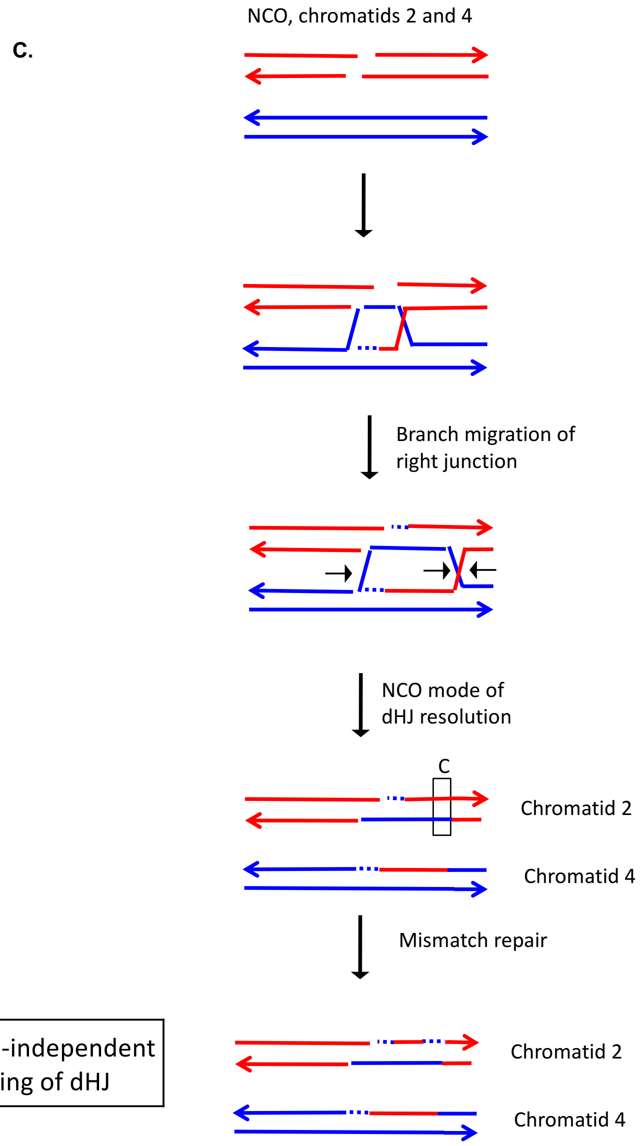

S77	NCO	Symmetric heteroduplex; conversion tract at end of heteroduplex tract	Branch migration; Mlh1-independent MMR; NCO processing of dHJ

S78

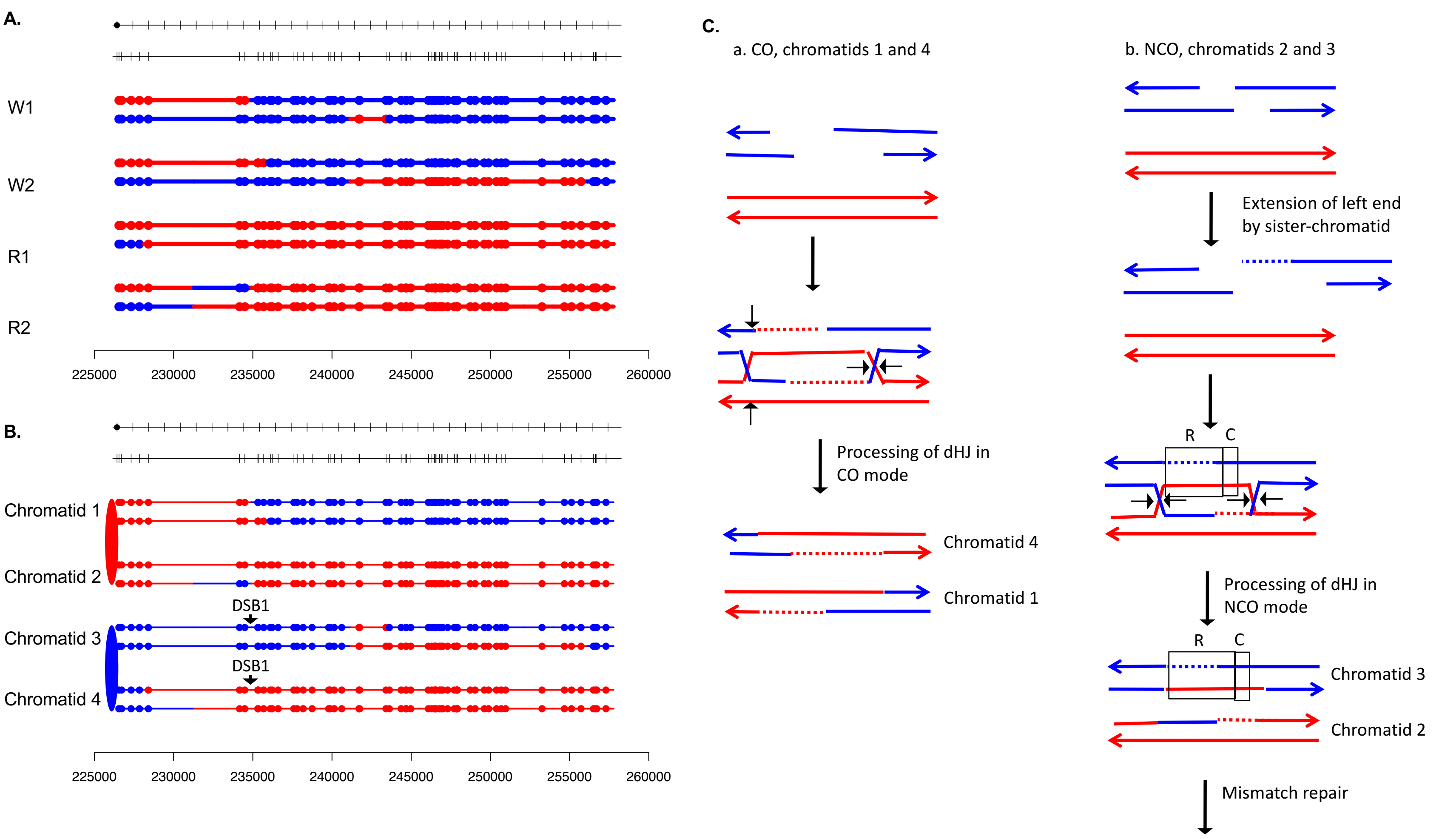

S78	NCO	Regions of homoduplex separating heteroduplex region from DSB site	Mlh1-independent MMR or template switching; resolution of dHJ in NCO mode
	CO	Region of conversion separating heteroduplex region from DSB site on chromatid 4	Mlh1-independent MMR or gap repair; resolution of dHJ in CO mode

S79

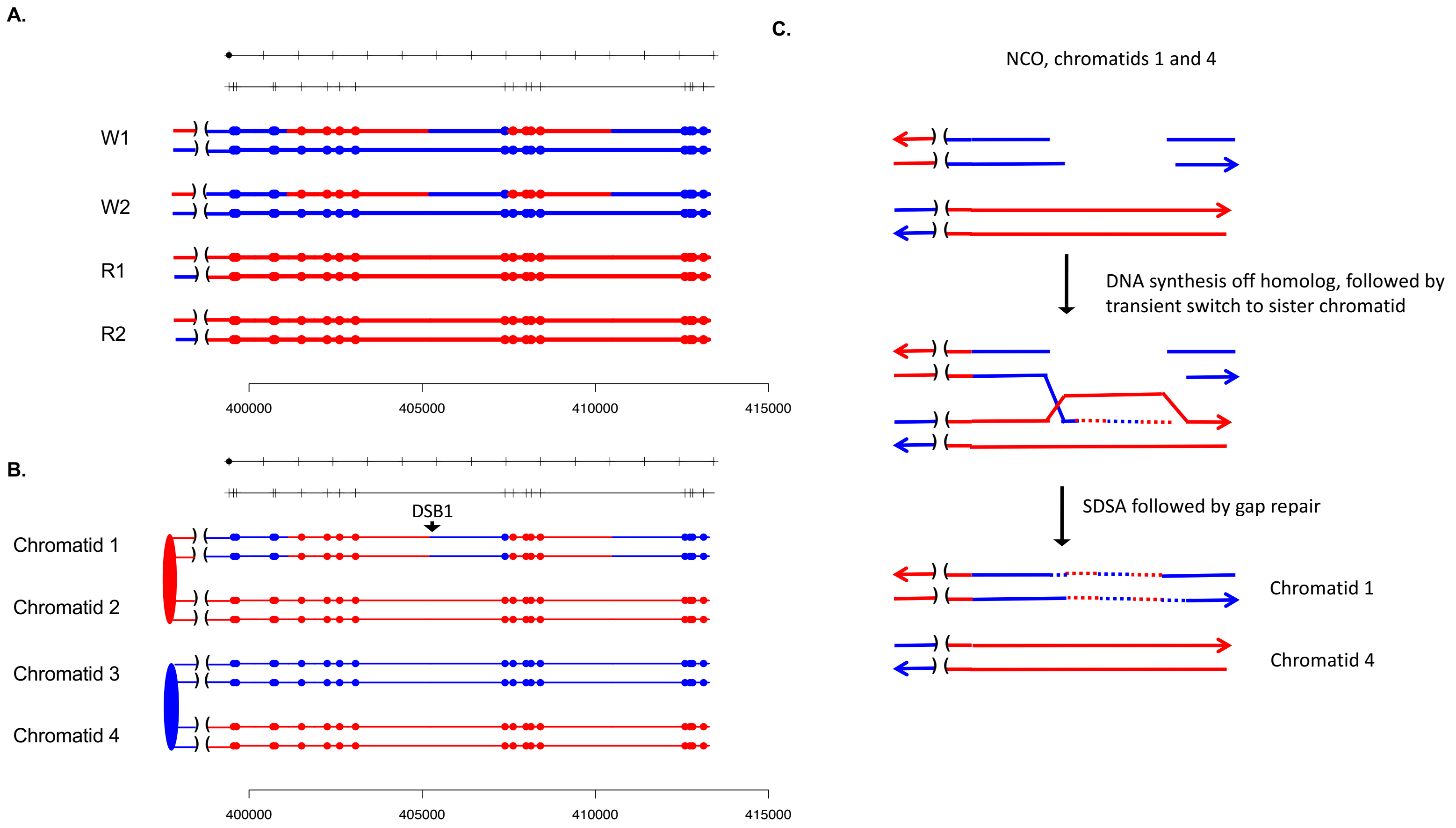

S79	NCO	In regions centromere-proximal to event, both homologs in white sector derived from one parental homolog and both in red sector derived from the other; regions of conversion and restoration homoduplexes on chromatid 1	Crossover on same chromosomes located centromere-proximal to the event in S79 (described in S78) produced centromere-proximal regions. Homoduplex regions produced by template switching or by Mlh1-independent MMR

S80

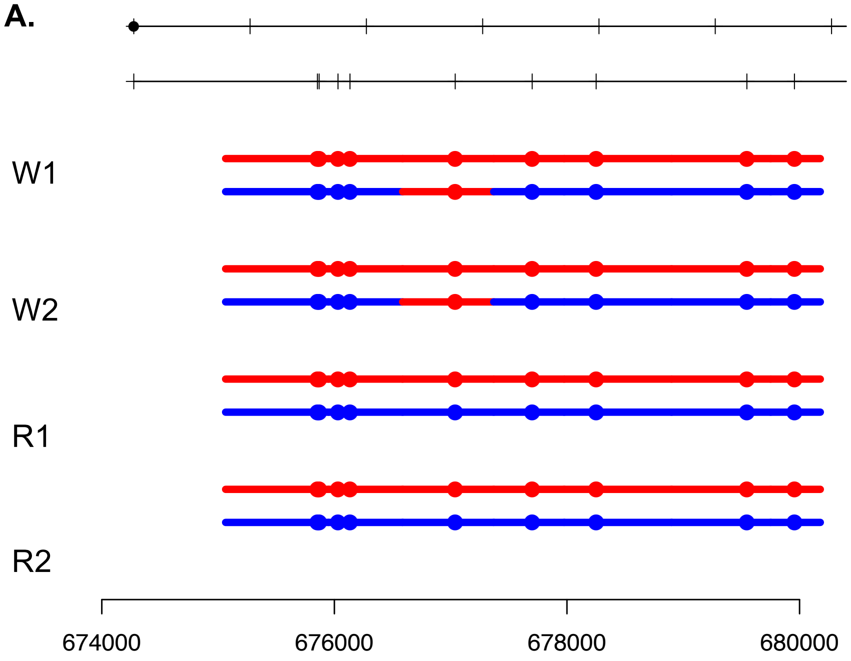
A.
W1
W2
R1
R2
674000
676000
678000
680000

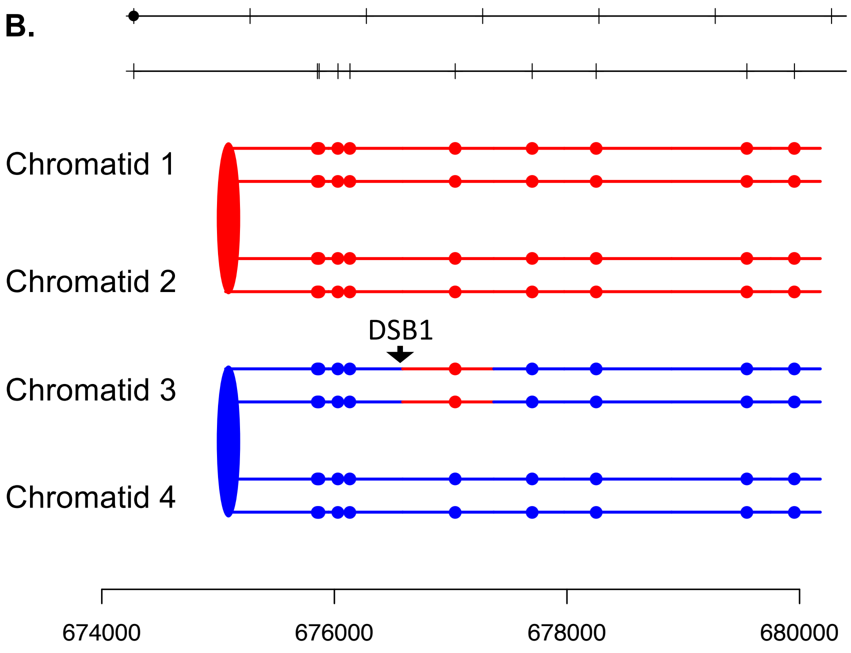
B.
Chromatid 1
Chromatid 2
DSB1
Chromatid 3
Chromatid 4
674000
676000
678000
680000

S81

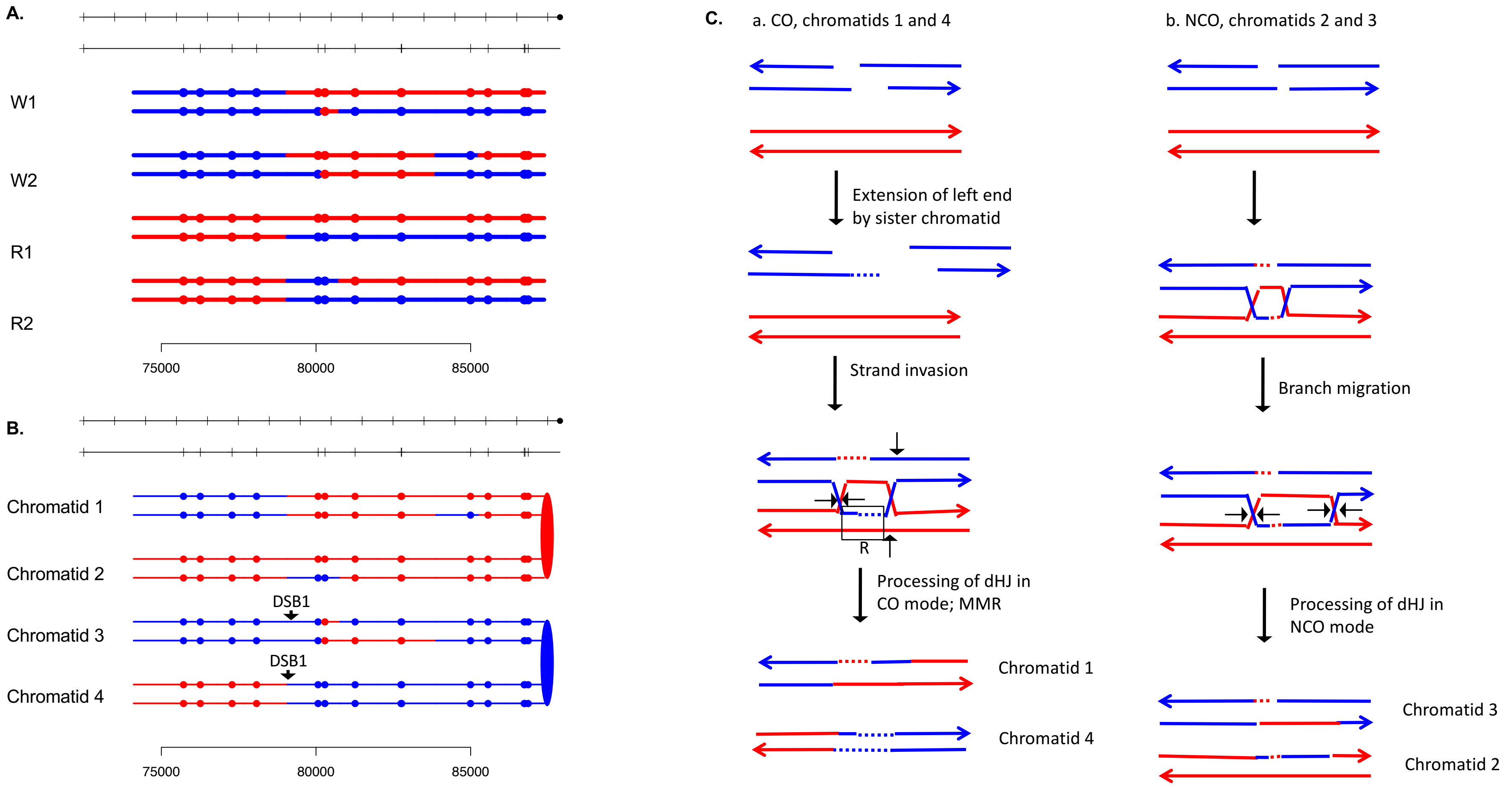

S81	CO	Long tracts of homoduplex on chromatids 1 and 4.	Extension of broken end by interaction with sister chromatid; Mlh1-independent restoration repair; resolution of dHJ in CO mode
	NCO	Heteroduplex DNA on same side of DSB site in NCO chromatids	Branch migration followed by processing of dHJ in CO mode

S82

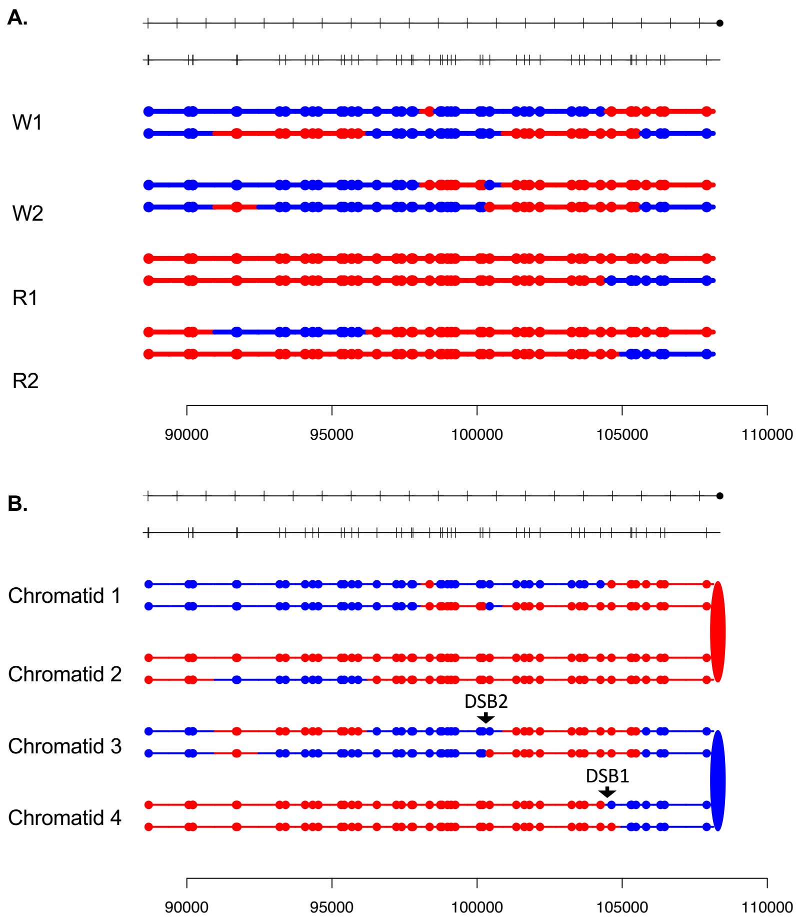

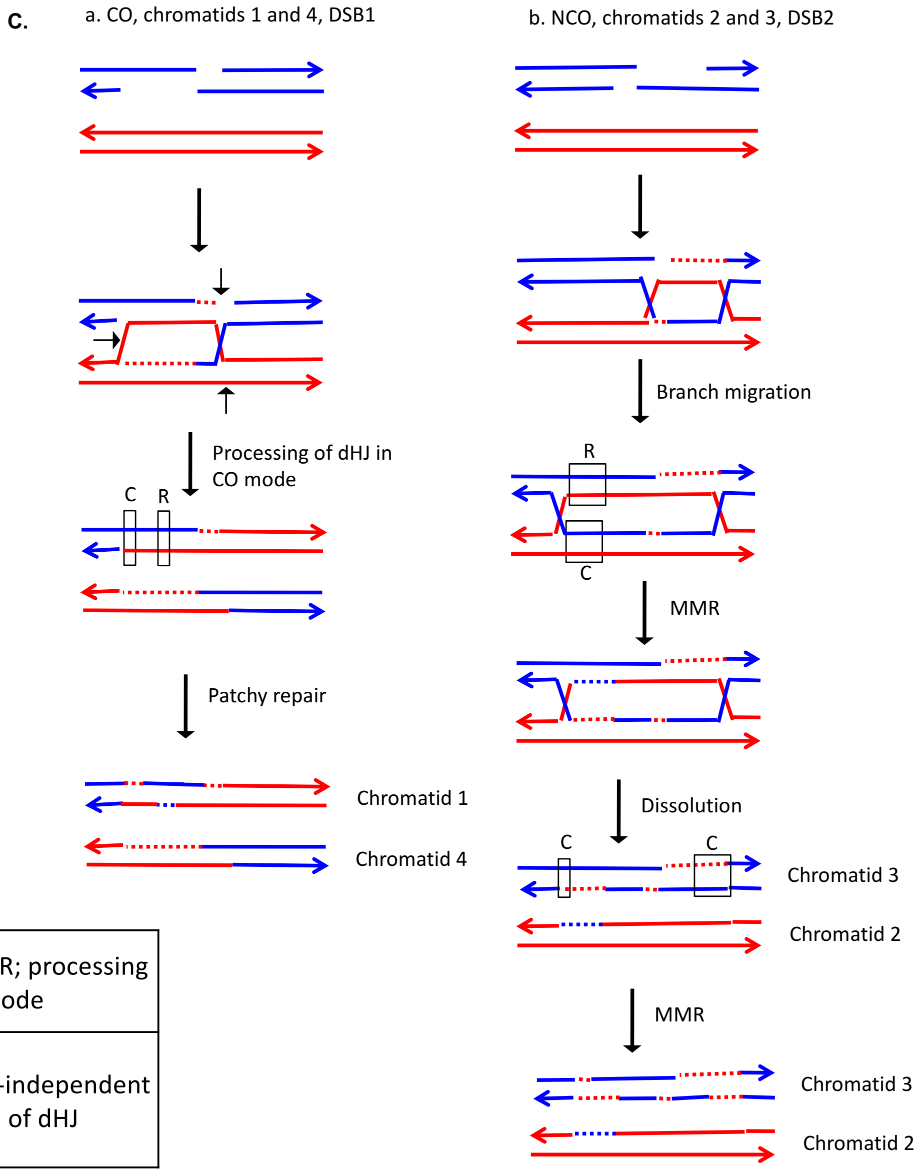

S82	CO	Heteroduplex with interspersed conversion and restoration tracts (chromatid 1); initiated at DSB1	Mlh1-independent MMR; processing of dHJ in CO mode
	NCO	Symmetric heteroduplex tracts; heteroduplex tract interspersed with homoduplex regions; strand switch on chromatid 3; initiated at DSB2	Branch migration; Mlh1-independent MMR; dissolution of dHJ

S83

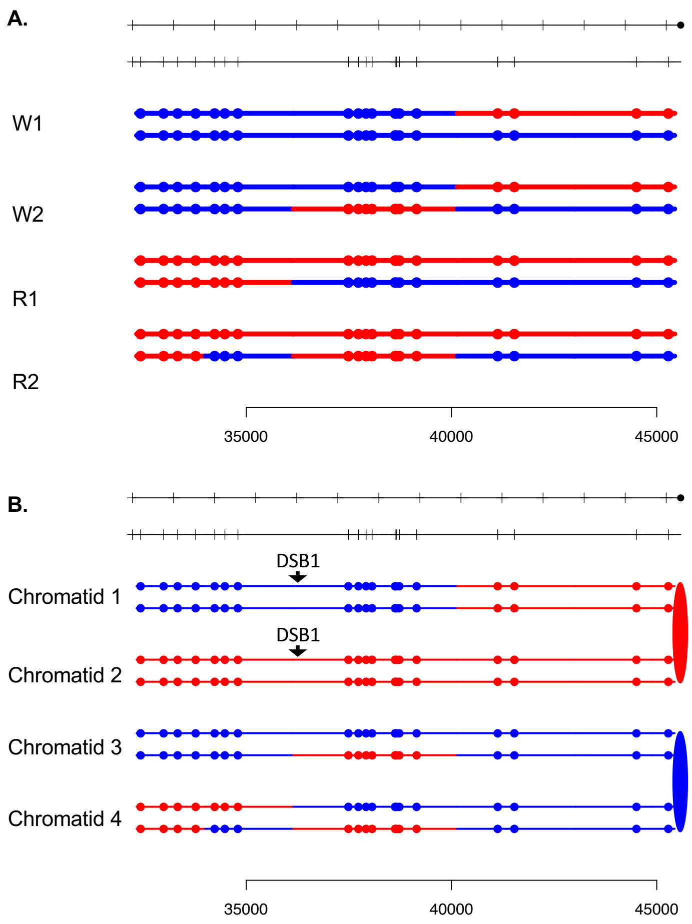

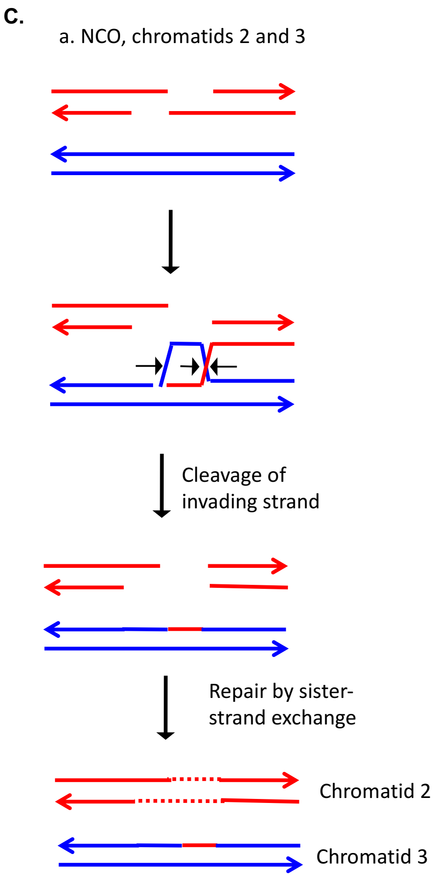

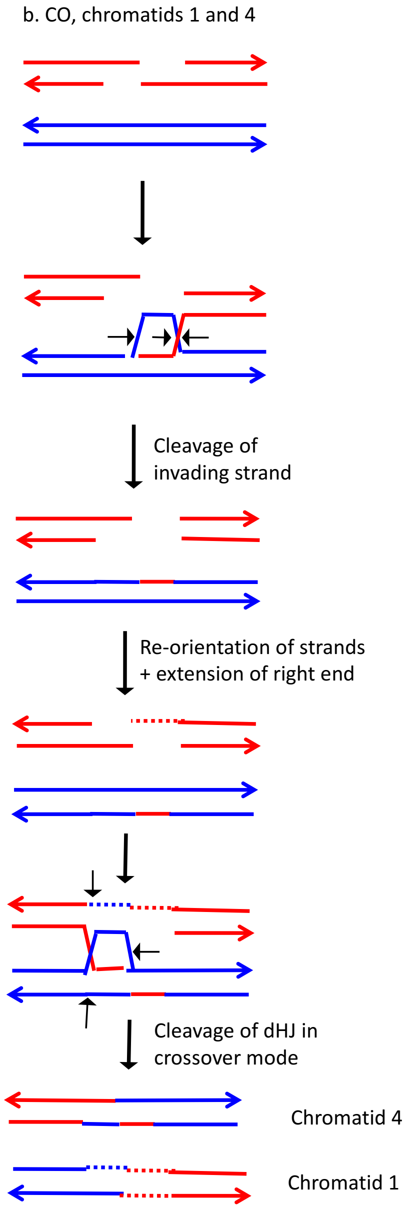

S83	CO	Strand switch of heteroduplex in CO chromatid 4	Invasion of the right broken end, followed by processing of junction; extension of right end by sister-chromatid interaction; invasion of the left end and processing dHJ in crossover mode
	NCO	DSB on red chromatid, but red chromatid acts as donor	Following strand invasion, junction cleaved before initiating DNA synthesis

S84

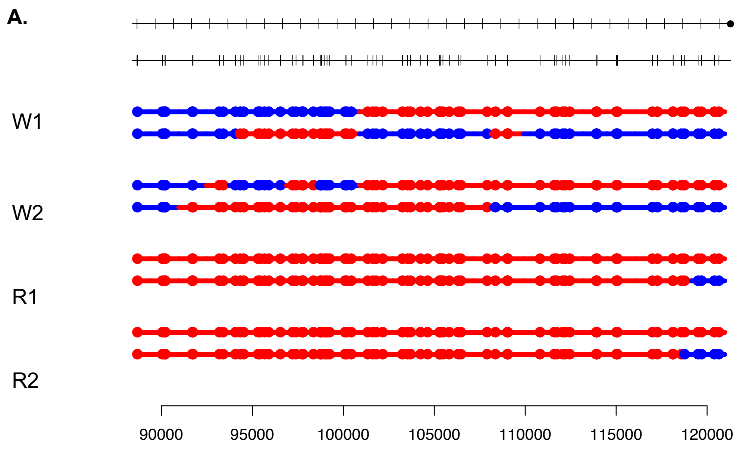

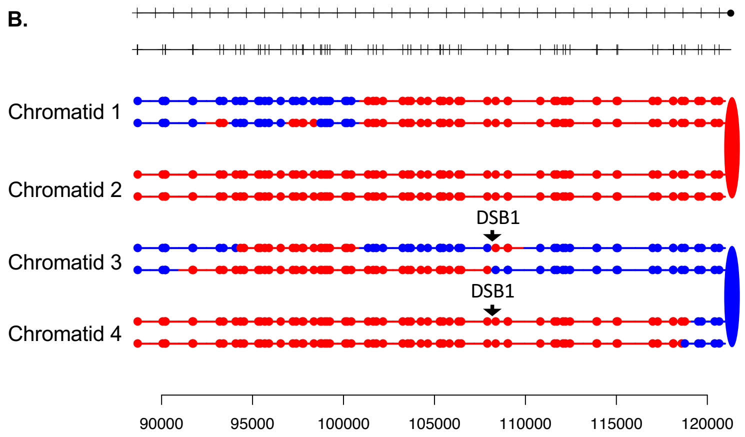

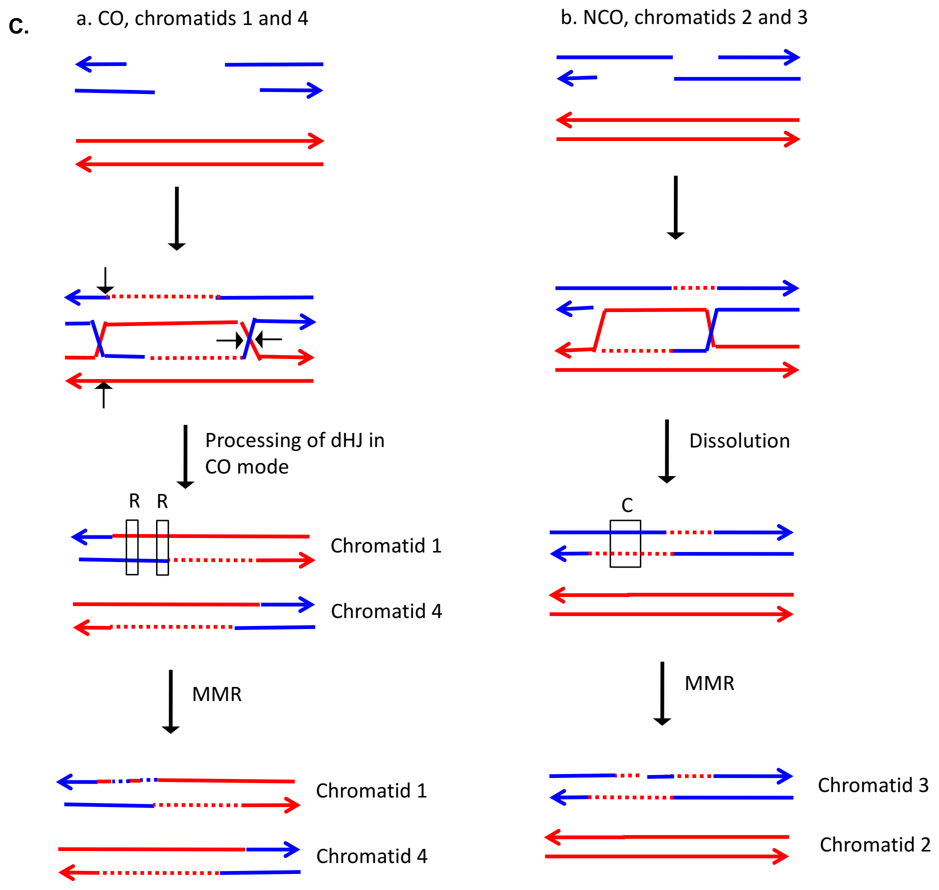

S84	CO	Long conversion tract spanning DSBs in both CO chromatids 1 and 4; heteroduplex in chromatid 1 interrupted by restoration tracts	Gap repair; Mlh1-independent MMR; resolution of dHJ in CO mode
	NCO	NCO chromatid 3 has strand switch of heteroduplexes; conversion event within heteroduplex	Resolution of dHJ by dissolution; Mlh1-independent MMR

S85

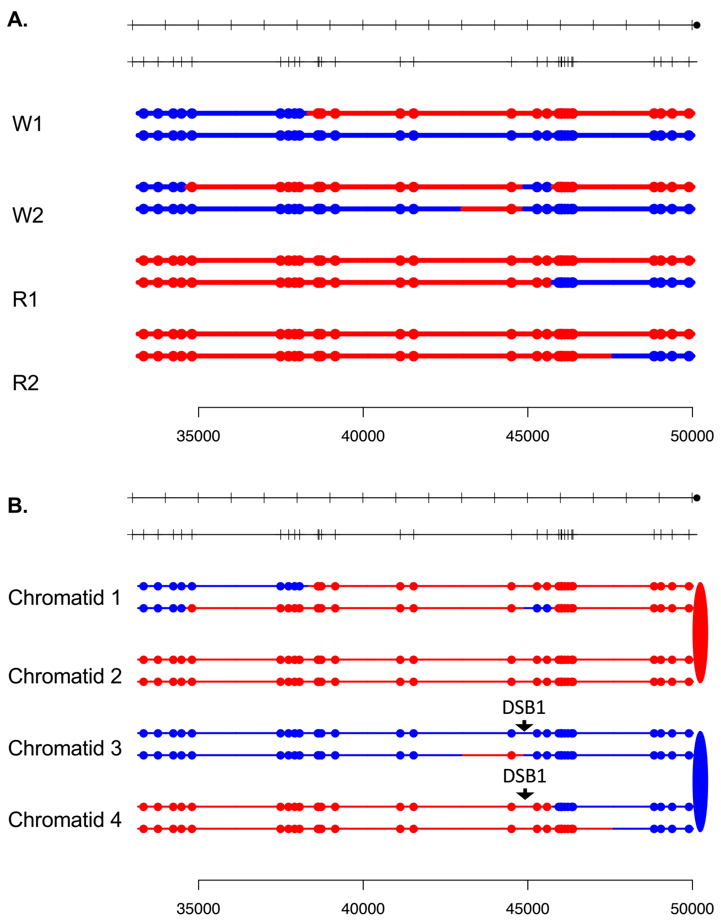

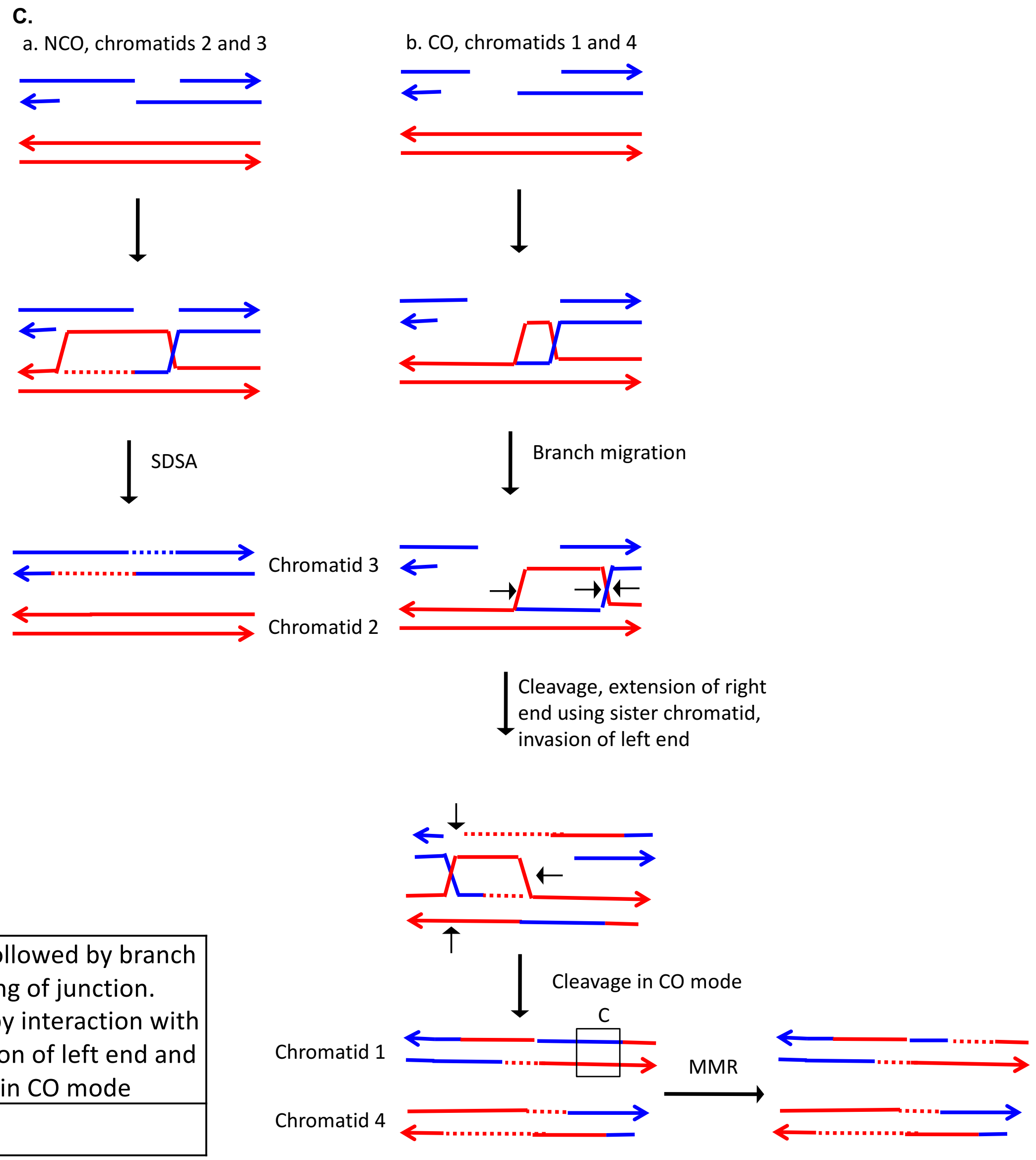

S85	CO	Strand switch in CO chromatid 1; heteroduplexes in trans on chromatids 1 and 4; long conversion tract adjacent to heteroduplex region in chromatid 1	Invasion of right end, followed by branch migration and cutting of junction. Extension of right end by interaction with sister chromatid; invasion of left end and resolution of dHJ in CO mode
	NCO	Simple heteroduplex	SDSA

S86

A.

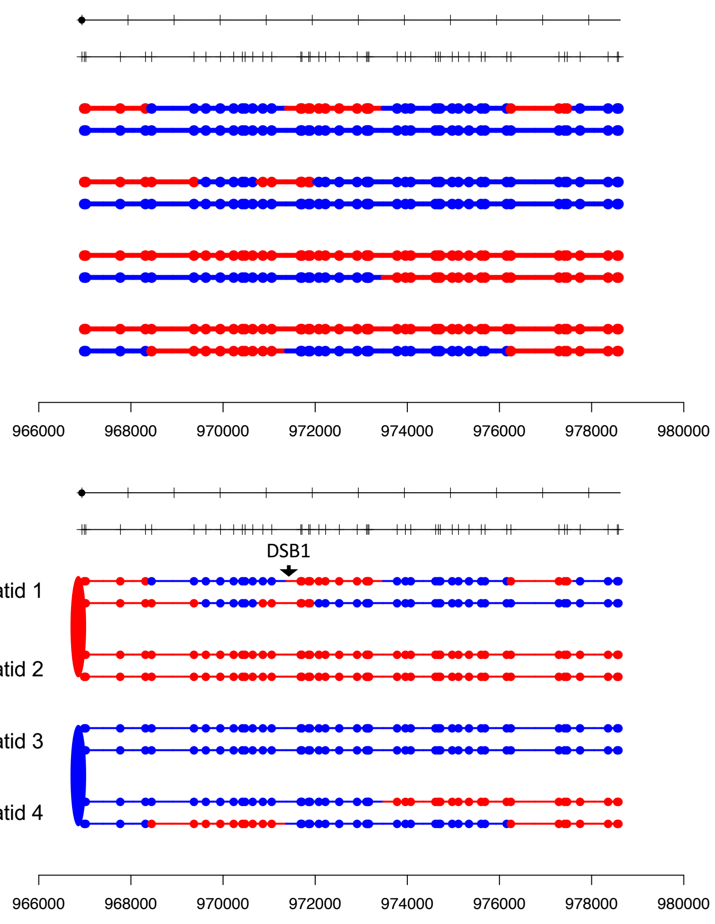

B.

C.

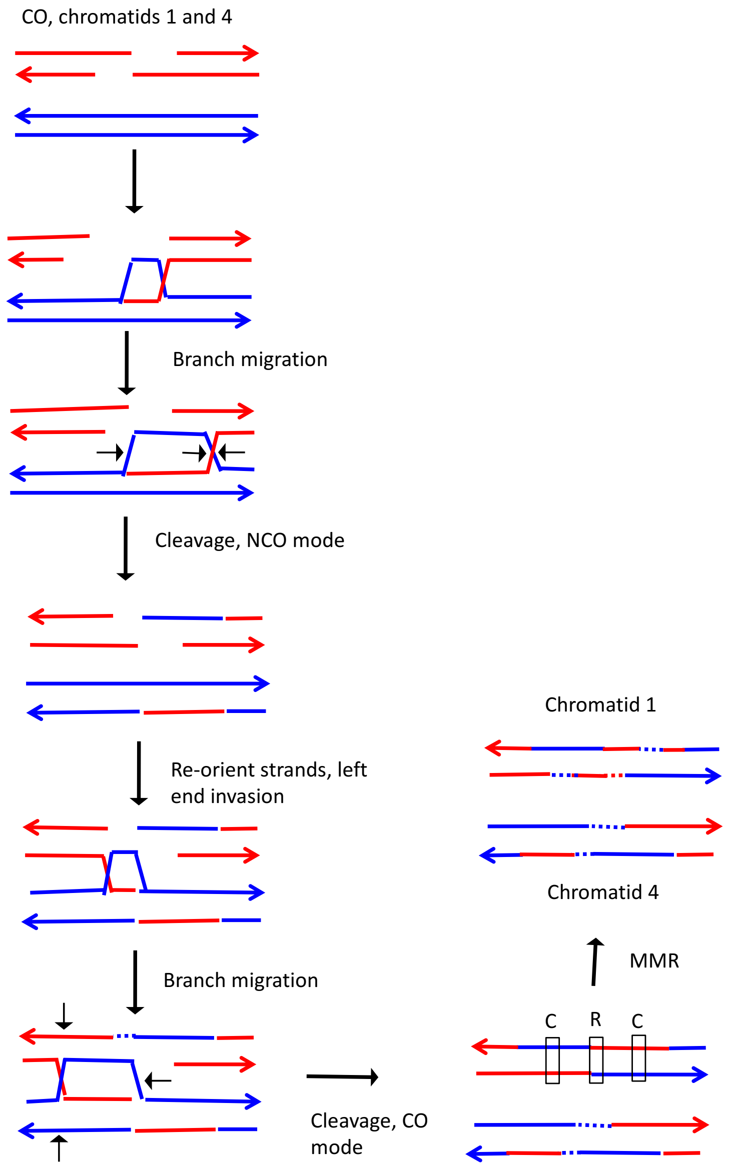

S86	CO	Strand switches in heteroduplexes in both CO chromatids 1 and 4; both chromatids have homoduplex regions interspersed with heteroduplex	Invasion of right broken end, followed by branch migration; resolution of junctions in NCO mode, followed by left end invasion and branch migration; cleavage in CO mode and Mlh1-independent MMR

S87

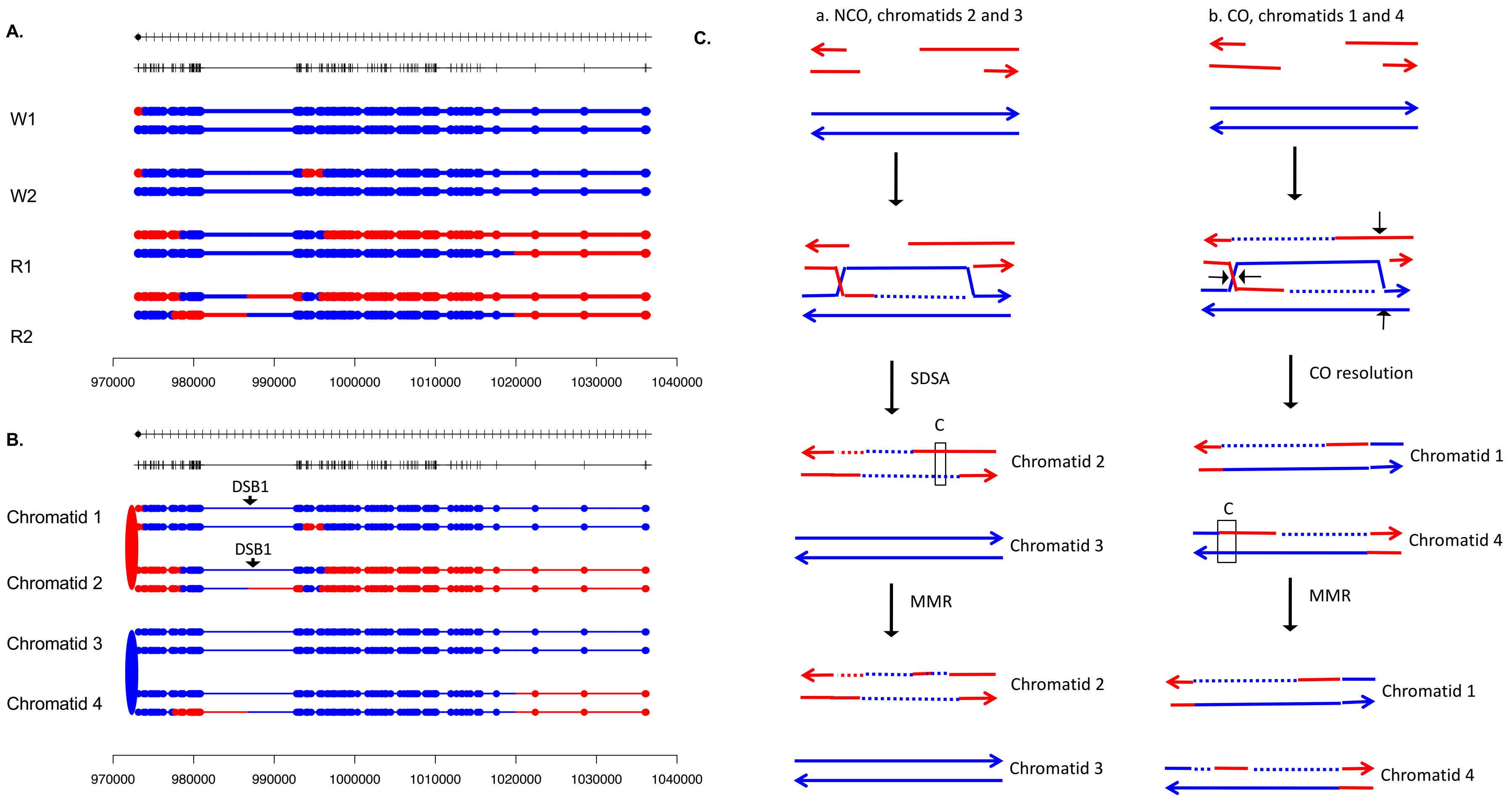

S87	CO	Large region of conversion spanning putative DSB site; uni-directional heteroduplexes on chromatids 1 and 4 propagated in opposite directions;; conversion tract distal to heterodupex tract on chromatid 4	Repair of large gap associated with HS4 hotspot; processing dHJ in CO mode; Mlh1-independent MMR
	NCO	Region of conversion spanning putative DSB site; conversion tract adjacent to heteroduplex	Repair of large gap; SDSA; Mlh1-independent MMR

S88

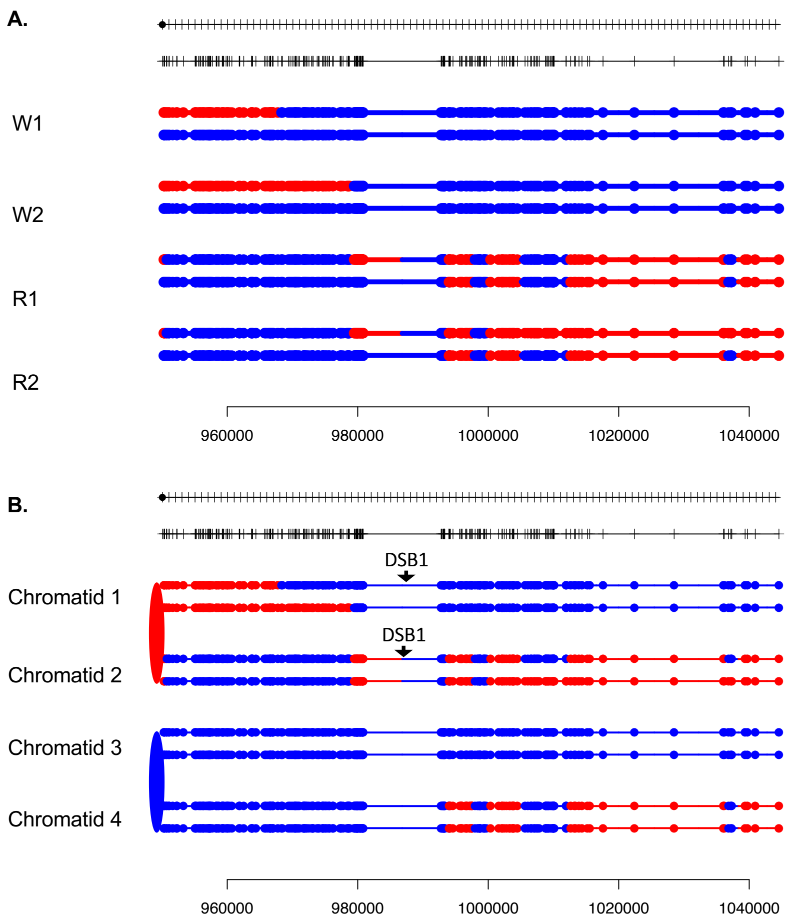

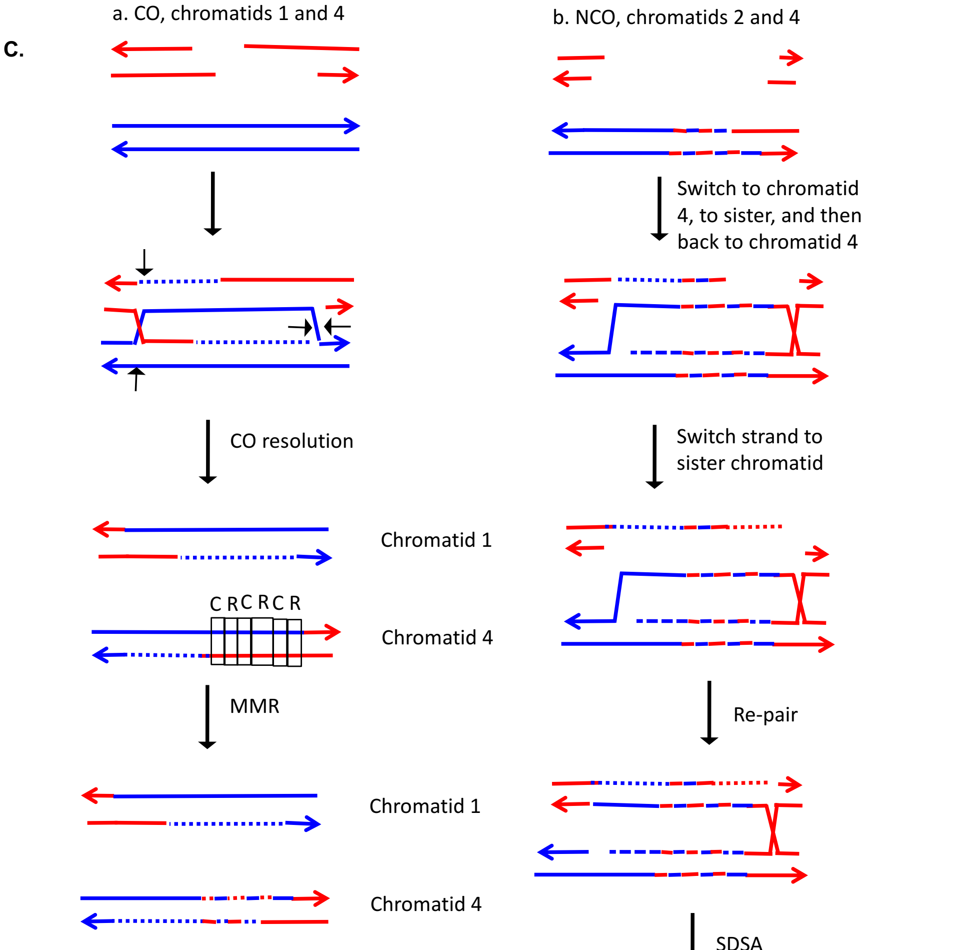

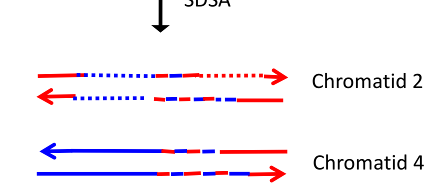

S88	CO	Region of conversion spanning putative DSB site; uni-directional heteroduplex tracts in on chromatids 1 and 4 propagated in different directions; multiple homoduplex tracts within heteroduplex tract on chromatid 4	Repair of large gap; patchy Mlh1-independent MMR; resolution of dHJ in CO mode
	NCO	Large conversion tract spanning putative DSB site; chromatid 3 has a pattern of heteroduplex and homoduplex tracts that is very similar to that of chromatid 4	Repair of large gap; chromatid 4 CO chromatid used as template for part of repair; also, switch of repair templates to sister chromatid

S89

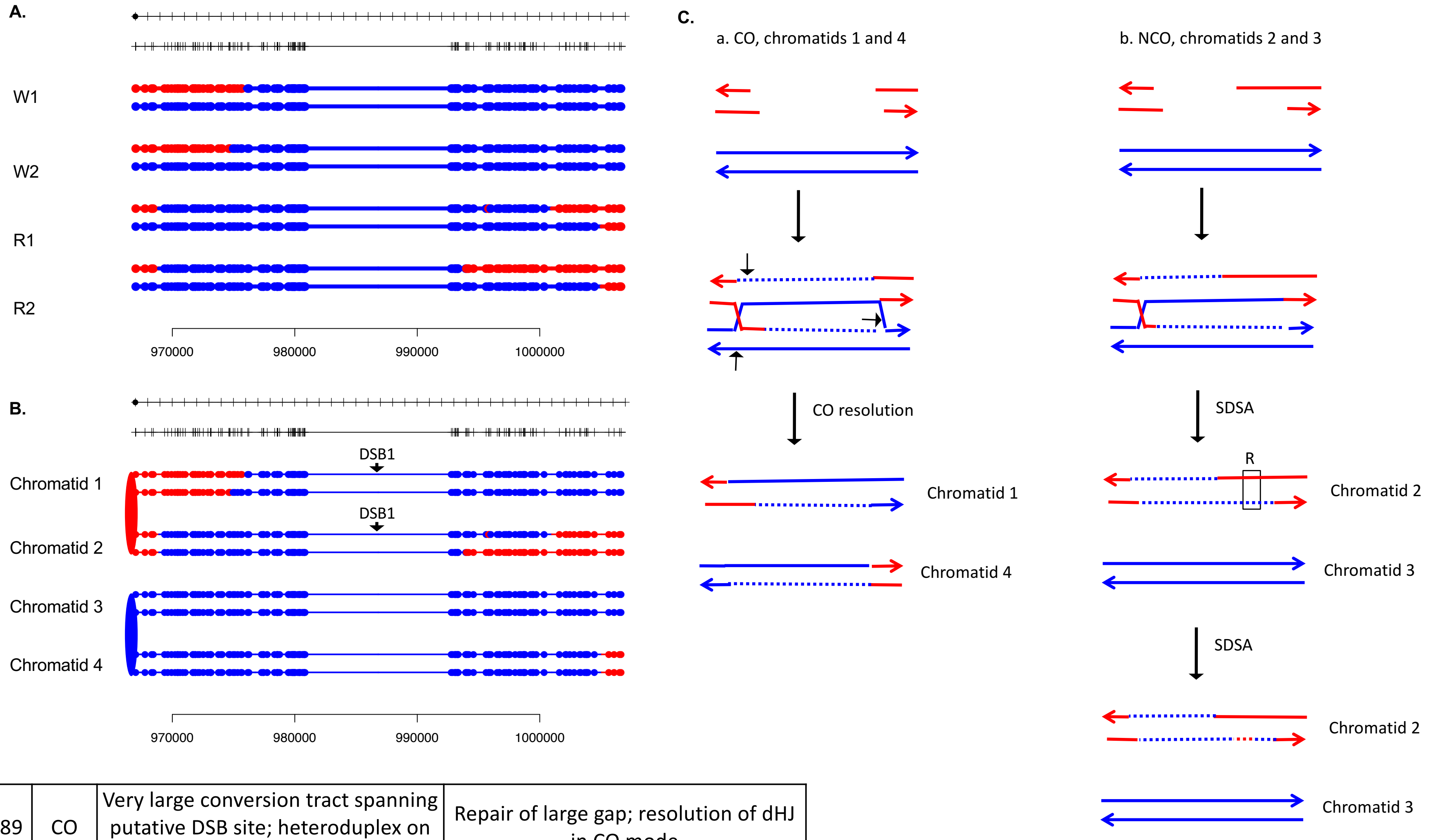

S89	CO	Very large conversion tract spanning putative DSB site; heteroduplex on chromatid 1 but not 4	Repair of large gap; resolution of dHJ in CO mode
	NCO	Very large conversion tract spanning putative DSB site; restoration tract in middle of heteroduplex	Repair of large gap; resolution of dHJ in NCO mode; Mlh1-independent MMR

S90

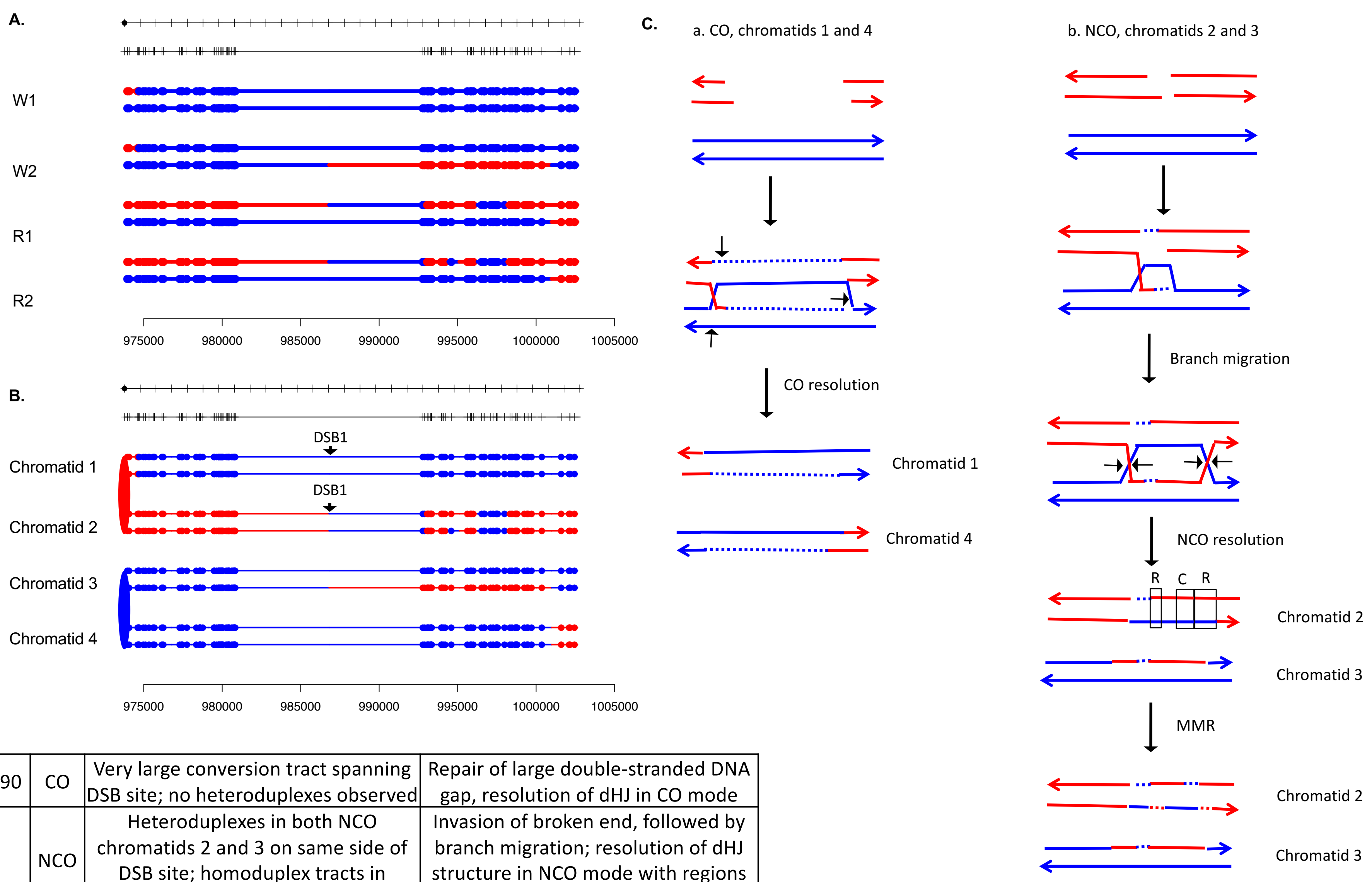

S90	CO	Very large conversion tract spanning DSB site; no heteroduplexes observed	Repair of large double-stranded DNA gap, resolution of dHJ in CO mode
	NCO	Heteroduplexes in both NCO chromatids 2 and 3 on same side of DSB site; homoduplex tracts in heteroduplex	Invasion of broken end, followed by branch migration; resolution of dHJ structure in NCO mode with regions of Mlh1-independent MMR

S91

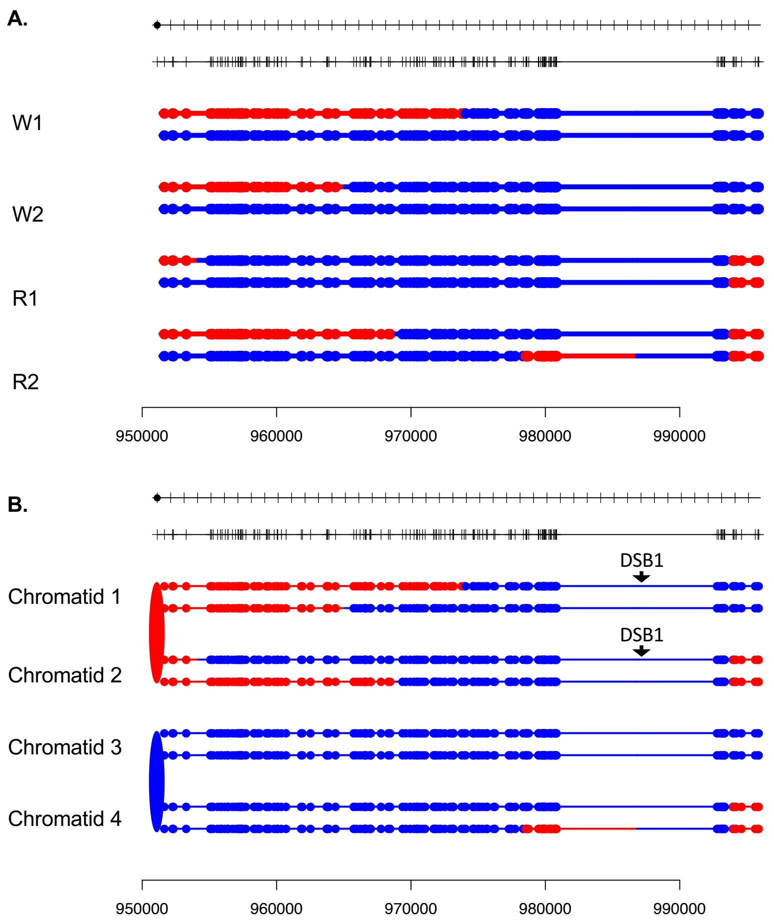

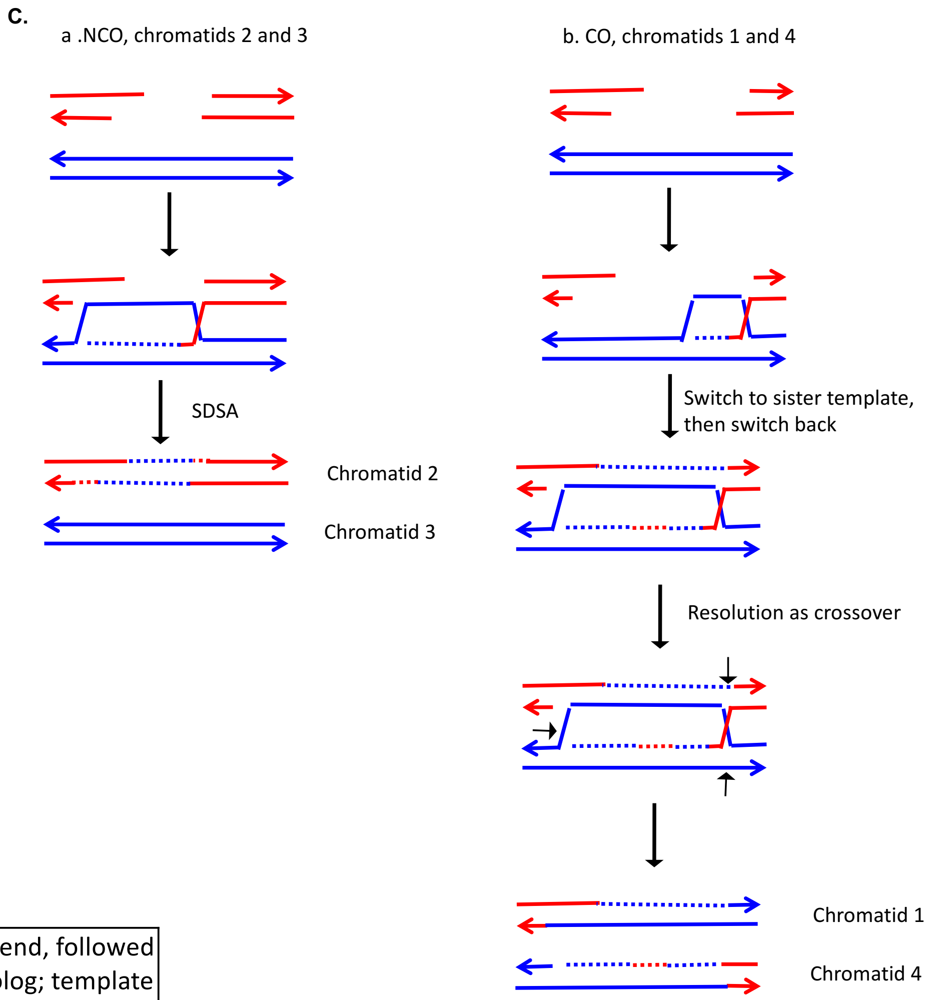

S91	CO	Heteroduplexes on both chromatids 1 and 4 on same side of putative DSB site; large conversion tract flanking DSB site	Invasion of right broken end, followed by synthesis off of homolog; template switch to sister chromatid, and then re-invasion of sister chromatid; resolution of dHJ in CO mode
	NCO	Large conversion tract spanning putative DSB site	Repair of large gap; SDSA
